# Wavelet Decomposition-Based Genomic Analysis of the Human Electrocardiogram

**DOI:** 10.64898/2026.05.20.26353725

**Published:** 2026-05-27

**Authors:** Salma Zainana, Larissa Lauer, Tuomo Kiiskinen, Robert Tibshirani, Trevor Hastie, Euan A. Ashley, Jack W. O’Sullivan, Manuel A. Rivas

## Abstract

The electrocardiogram (ECG) encodes the electrical activity of the heart across multiple timescales, yet standard clinical analysis collapses this rich signal into a handful of scalar measurements that discard most of the waveform’s structure. Whether the frequency signals lost in this reduction carry heritable biological information relevant to cardiovascular disease risk remains unclear. Here we decompose resting 12-lead ECGs from 47,052 White British UK Biobank participants into 84 frequency-specific energy features using Daubechies-6 wavelet analysis across 12 leads and 7 decomposition levels, and perform independent genome-wide association analyses on each feature. We identify 67 independent loci (p < 5 × 10⁻⁸) and refine these to 101 high-confidence causal variants (posterior inclusion probability > 0.80) through Bayesian fine-mapping; associated loci converge on genes governing cardiac conduction and myocardial integrity, including SCN5A, TTN, KCNQ1, and DSP, alongside less-characterized cardiomyopathy candidates. SNP-based heritability estimates range from 0.03 to 0.26, with the strongest signals in mid-frequency bands (D6–D4, ∼4–32 Hz) of Lead I and aVR, and strong inter-lead genetic correlations indicate a coordinated genetic architecture underlying the waveform. Integrating these features with FinnGen R12 cardiovascular phenotypes reveals genetic correlations reaching 0.56 with heart failure, driven predominantly by energy in the highest-frequency band (D1, 125–250 Hz) — a spectral range routinely filtered from clinical ECGs and previously regarded as acquisition noise. These results reframe the electrocardiogram as a multi-frequency genetic phenotype, expand the set of cardiac loci discoverable from ECG data, and implicate high-frequency cardiac electrical activity as an underexplored dimension of cardiovascular disease risk.

## Full Text Availability

The license terms selected by the author(s) for this preprint version do not permit archiving in PMC. The full text is available from the preprint server.

