# Supplementary material for: Wavelet Decomposition-Based Genomic Analysis of the Human Electrocardiogram
Source: medRxiv. 2026 Jun 7:2026.05.20.26353725. Preprint. [Version 3] doi: 10.64898/2026.05.20.26353725 (PMC13228739; doi:10.64898/2026.05.20.26353725)
Supplement: Supplementary file 1 [file NIHPP2026.05.20.26353725v3-supplement-1.pdf]

medRxiv preprint doi: https://doi.org/10.64898/2026.05.20.26353725; this version posted June 7, 2026. The copyright holder for this preprint (which was not certified by peer review) is the author/funder, who has granted medRxiv a license to display the preprint in perpetuity.
It is made available under a CC-BY 4.0 International license .

Supplementary Material

Supplementary Figures 1

Figures S1 present the complete set of Manhattan plots from the genome-wide association analyses of all 84 wavelet-derived ECG energy features (12 leads × 6 detail levels + 1 approximation level). Each plot displays the chromosomal position of SNPs versus their association significance ($-\log_{10}(p)$), with genome-wide and suggestive thresholds indicated. These figures complement the main Manhattan plot shown in Figure 2 by providing the full visual results for each lead and decomposition level, enabling detailed inspection of frequency- and lead-specific genetic association patterns.

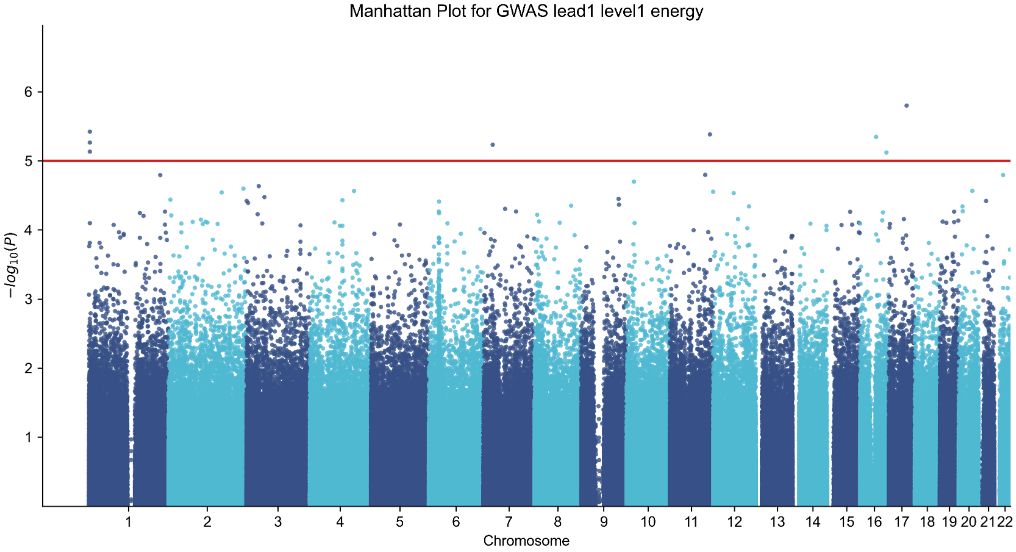

medRxiv preprint doi: https://doi.org/10.64898/2026.05.20.26353725; this version posted June 7, 2026. The copyright holder for this preprint (which was not certified by peer review) is the author/funder, who has granted medRxiv a license to display the preprint in perpetuity.
It is made available under a CC-BY 4.0 International license .

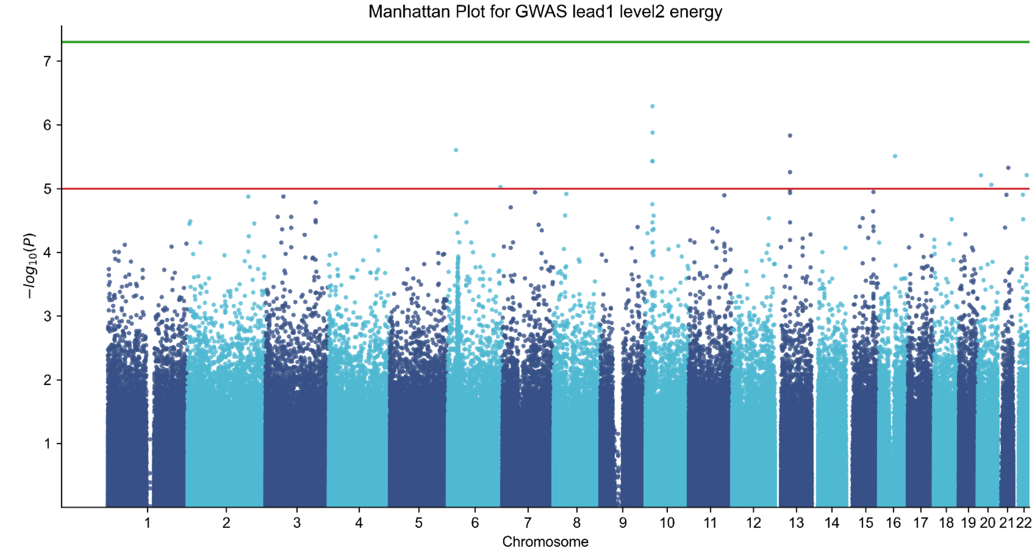

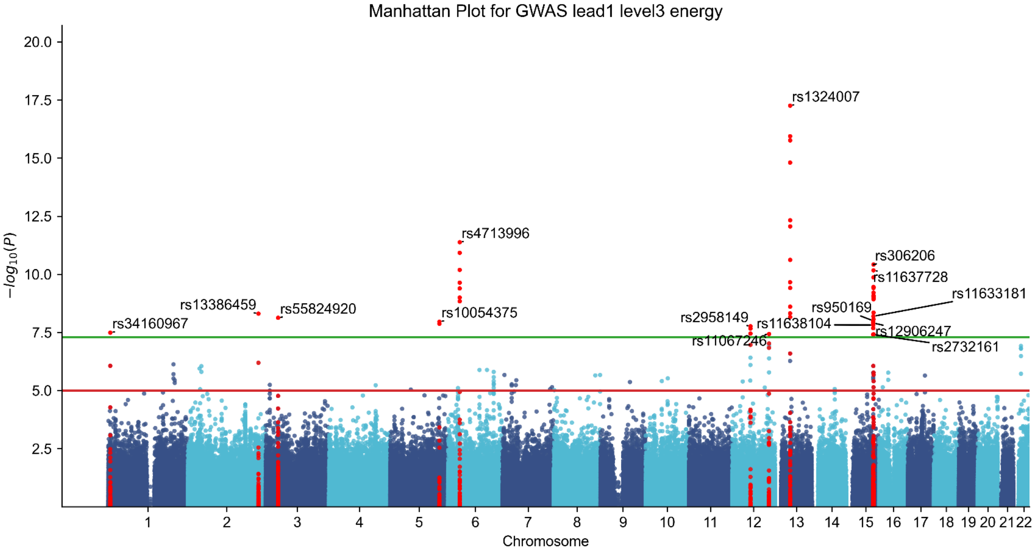

medRxiv preprint doi: https://doi.org/10.64898/2026.05.20.26353725; this version posted June 7, 2026. The copyright holder for this preprint
(which was not certified by peer review) is the author/funder, who has granted medRxiv a license to display the preprint in perpetuity.
It is made available under a CC-BY 4.0 International license .

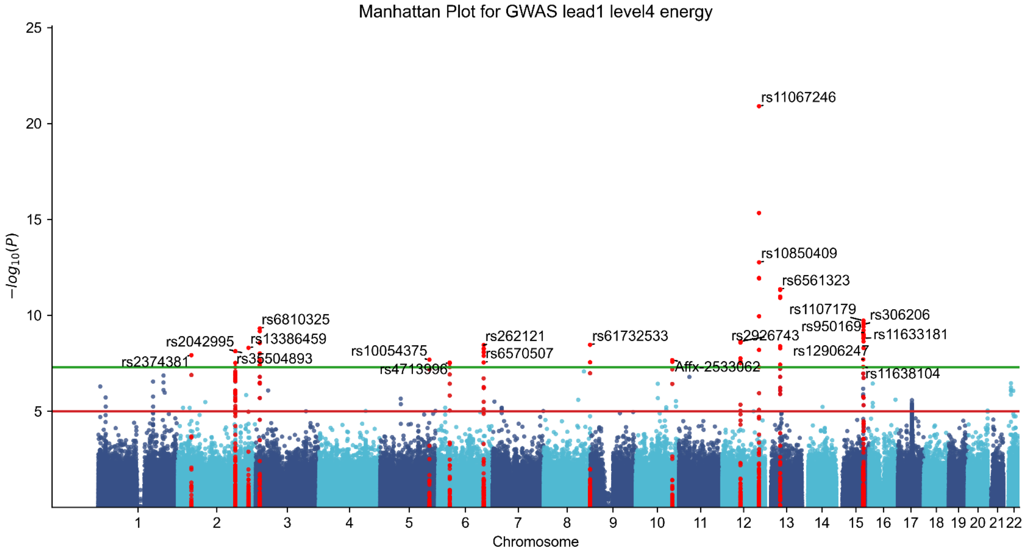

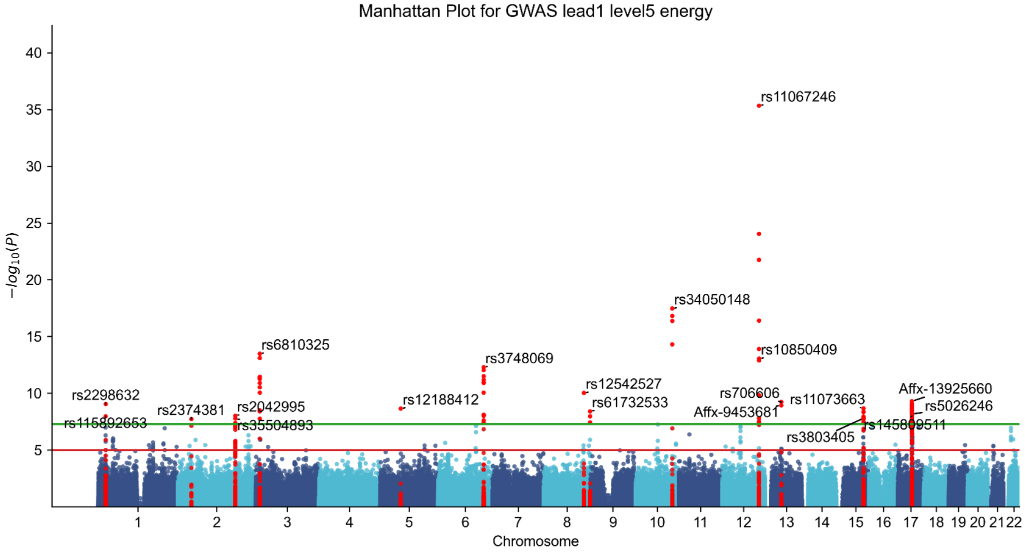

medRxiv preprint doi: https://doi.org/10.64898/2026.05.20.26353725; this version posted June 7, 2026. The copyright holder for this preprint
(which was not certified by peer review) is the author/funder, who has granted medRxiv a license to display the preprint in perpetuity.
It is made available under a CC-BY 4.0 International license .

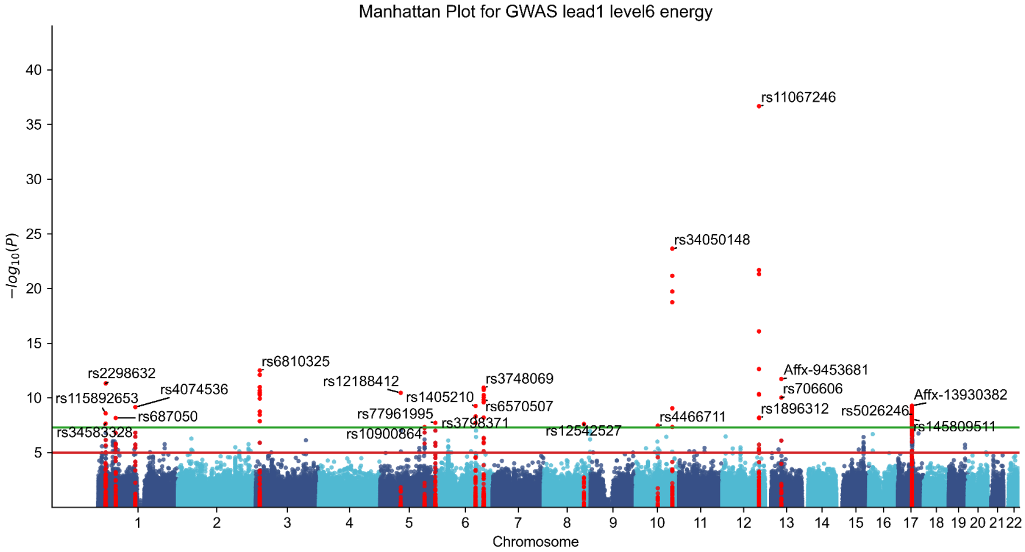

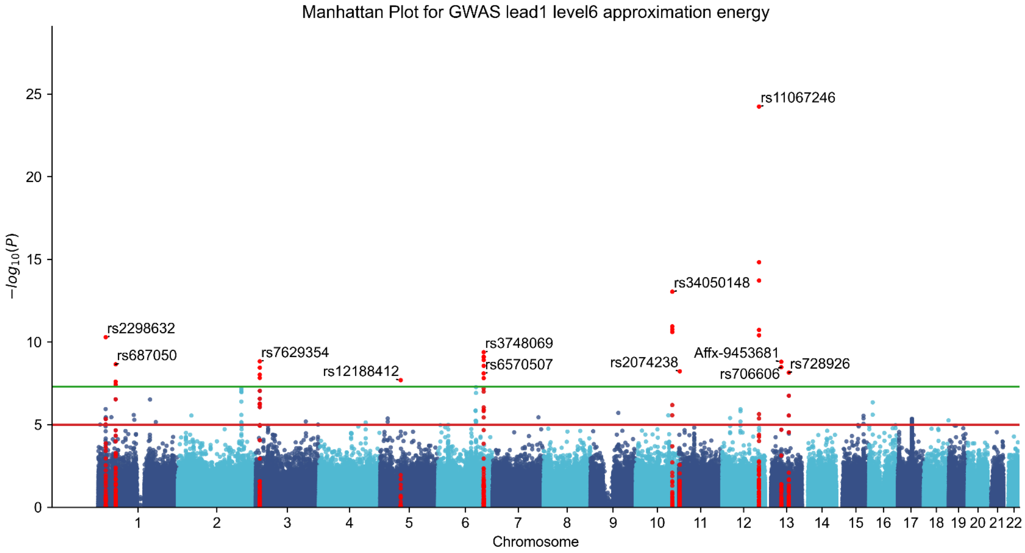

medRxiv preprint doi: https://doi.org/10.64898/2026.05.20.26353725; this version posted June 7, 2026. The copyright holder for this preprint (which was not certified by peer review) is the author/funder, who has granted medRxiv a license to display the preprint in perpetuity.
It is made available under a CC-BY 4.0 International license .

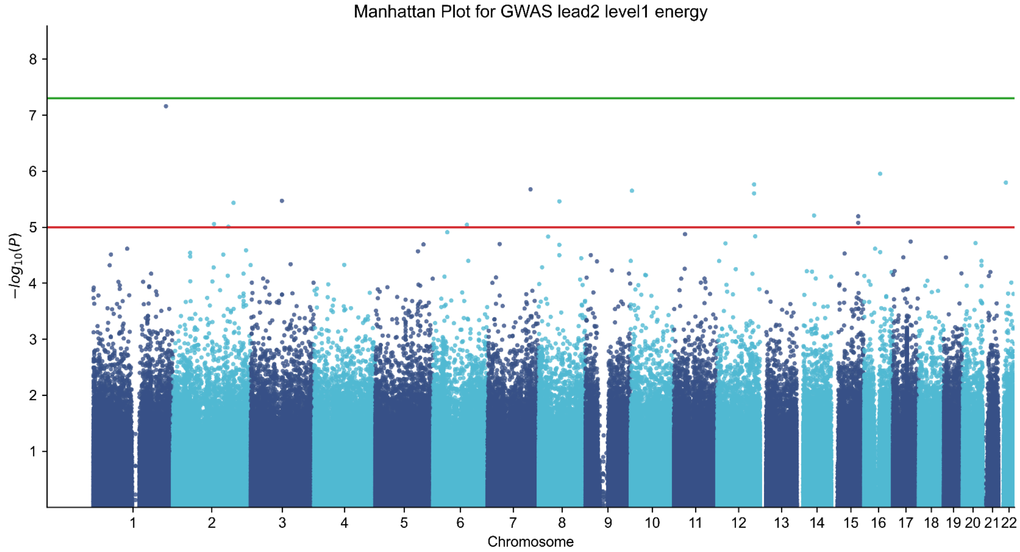

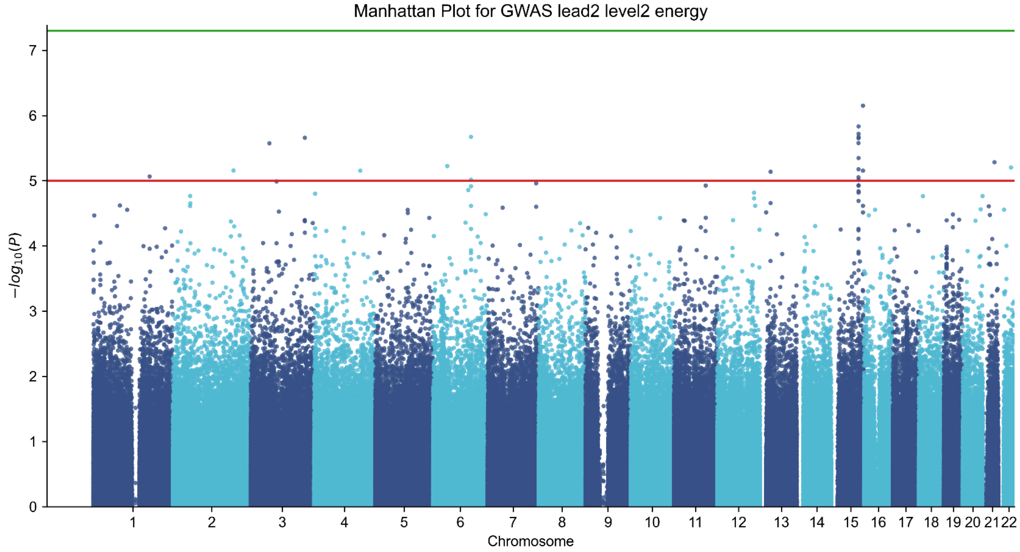

medRxiv preprint doi: https://doi.org/10.64898/2026.05.20.26353725; this version posted June 7, 2026. The copyright holder for this preprint (which was not certified by peer review) is the author/funder, who has granted medRxiv a license to display the preprint in perpetuity.
It is made available under a CC-BY 4.0 International license .

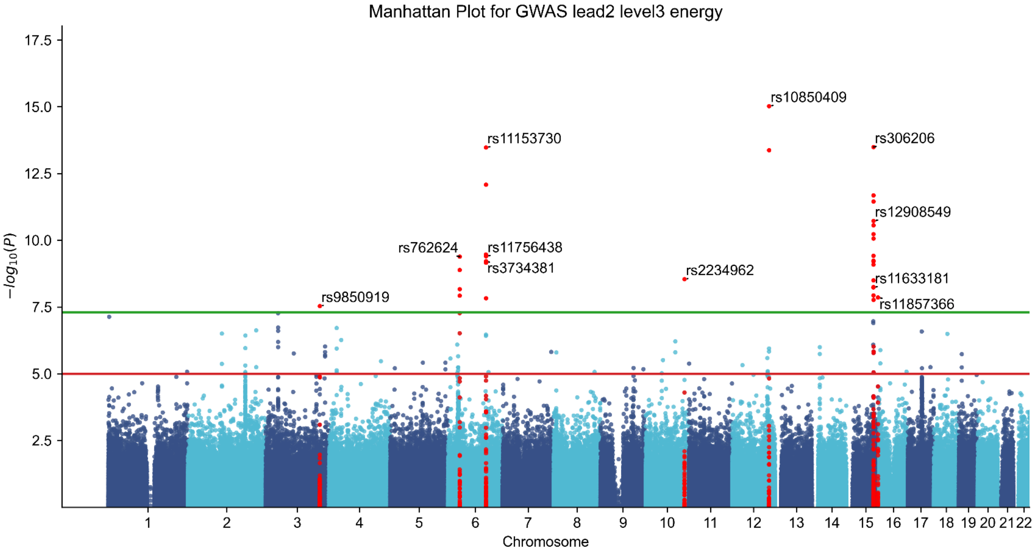

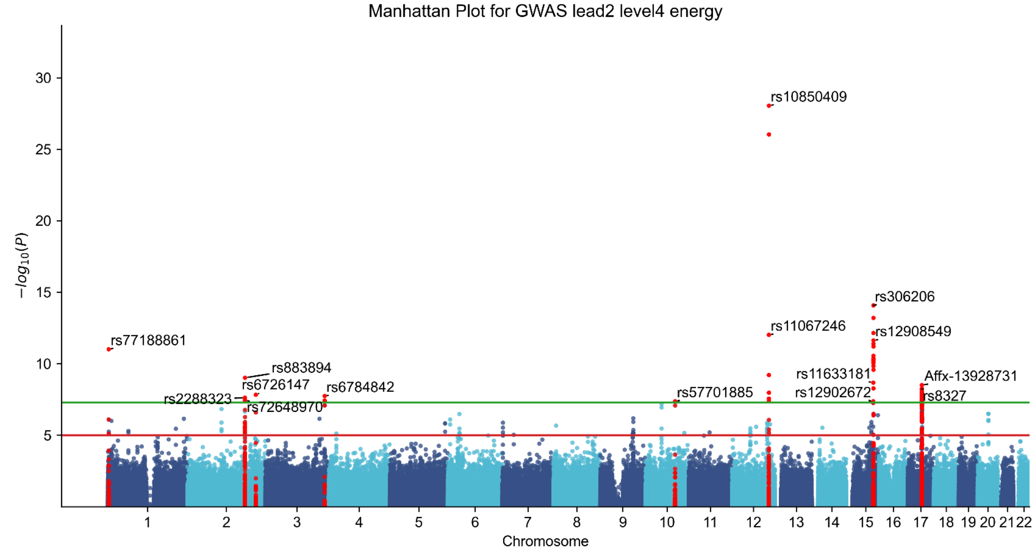

medRxiv preprint doi: https://doi.org/10.64898/2026.05.20.26353725; this version posted June 7, 2026. The copyright holder for this preprint (which was not certified by peer review) is the author/funder, who has granted medRxiv a license to display the preprint in perpetuity.
It is made available under a CC-BY 4.0 International license .

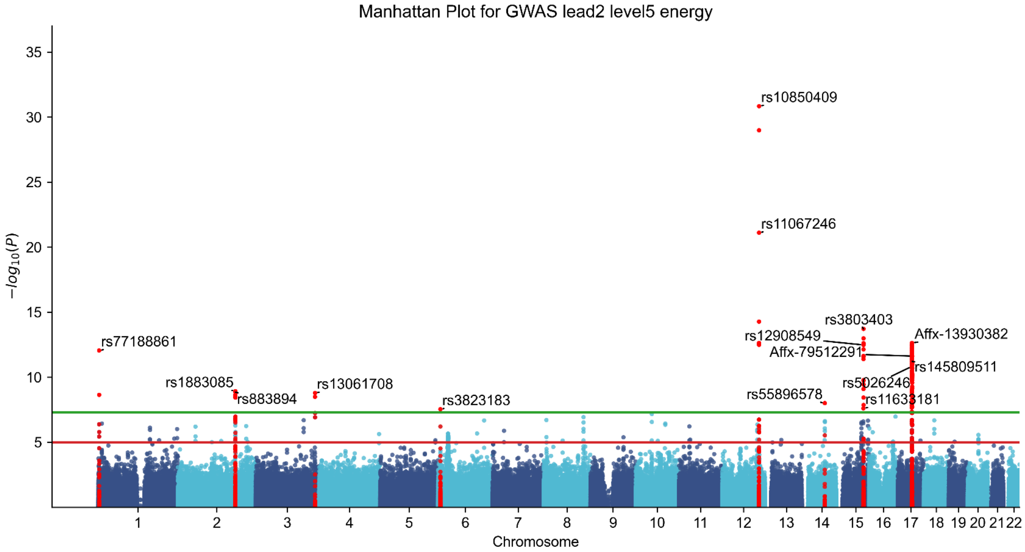

medRxiv preprint doi: https://doi.org/10.64898/2026.05.20.26353725; this version posted June 7, 2026. The copyright holder for this preprint (which was not certified by peer review) is the author/funder, who has granted medRxiv a license to display the preprint in perpetuity.
It is made available under a CC-BY 4.0 International license .

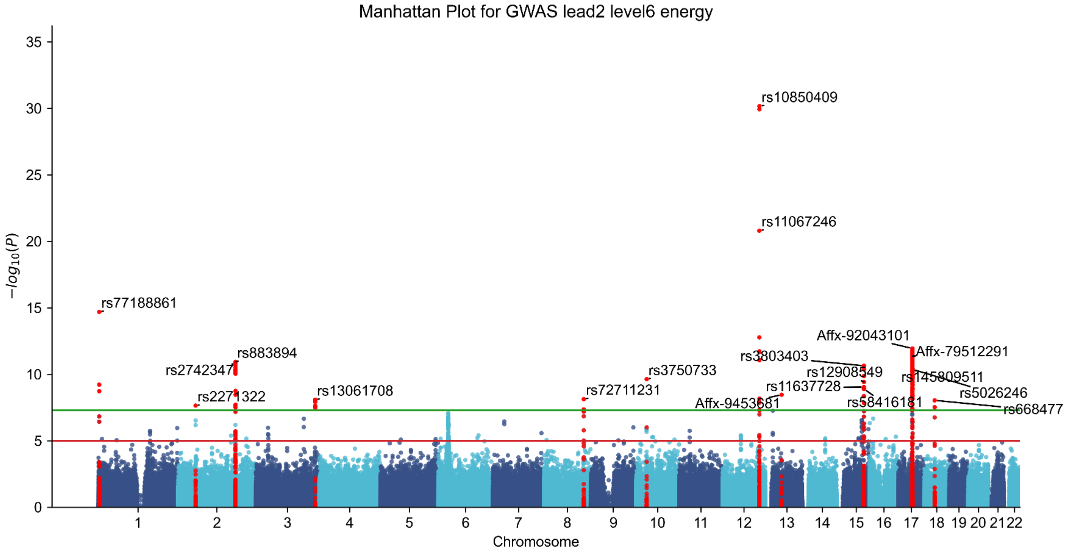

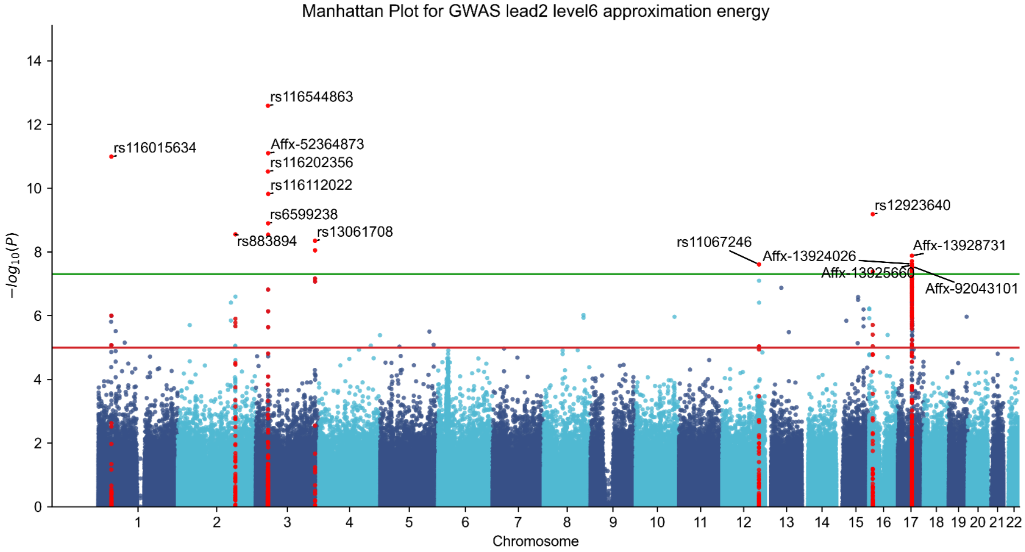

medRxiv preprint doi: https://doi.org/10.64898/2026.05.20.26353725; this version posted June 7, 2026. The copyright holder for this preprint (which was not certified by peer review) is the author/funder, who has granted medRxiv a license to display the preprint in perpetuity.
It is made available under a CC-BY 4.0 International license .

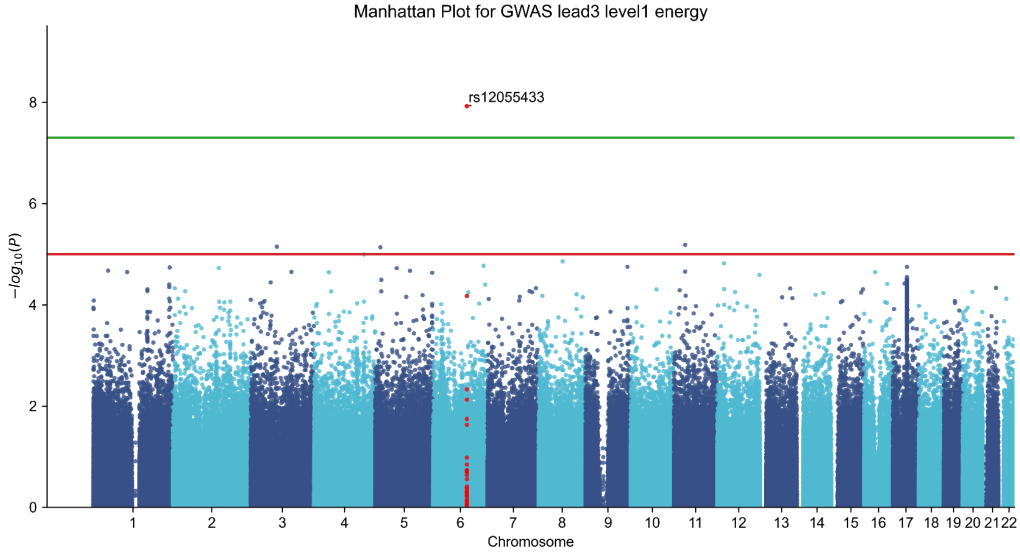

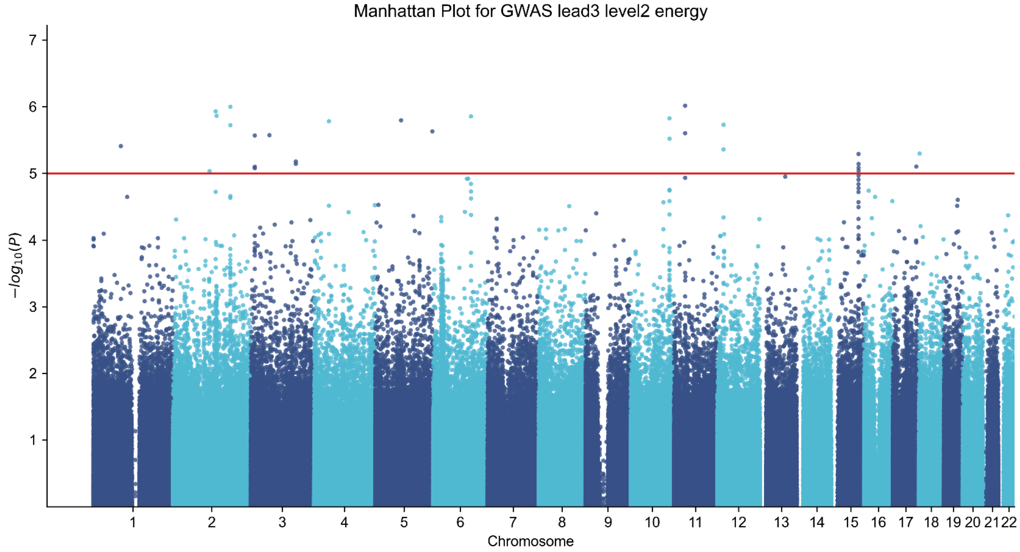

medRxiv preprint doi: https://doi.org/10.64898/2026.05.20.26353725; this version posted June 7, 2026. The copyright holder for this preprint (which was not certified by peer review) is the author/funder, who has granted medRxiv a license to display the preprint in perpetuity.
It is made available under a CC-BY 4.0 International license .

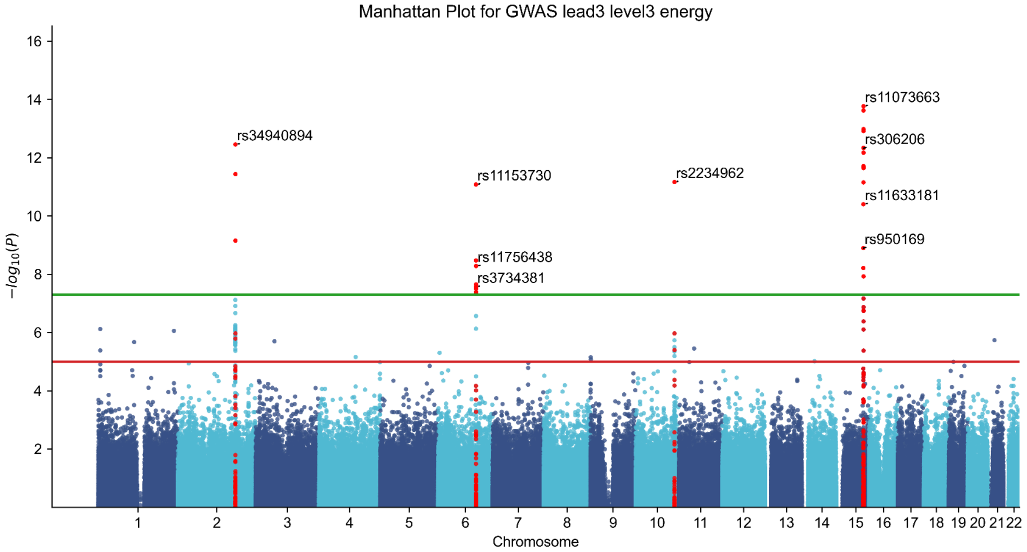

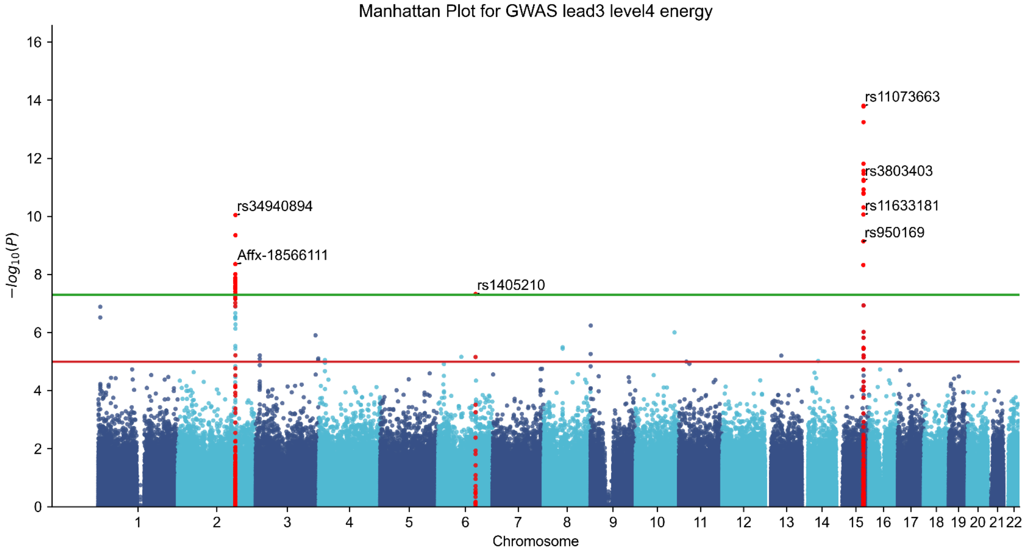

medRxiv preprint doi: https://doi.org/10.64898/2026.05.20.26353725; this version posted June 7, 2026. The copyright holder for this preprint (which was not certified by peer review) is the author/funder, who has granted medRxiv a license to display the preprint in perpetuity.
It is made available under a CC-BY 4.0 International license .

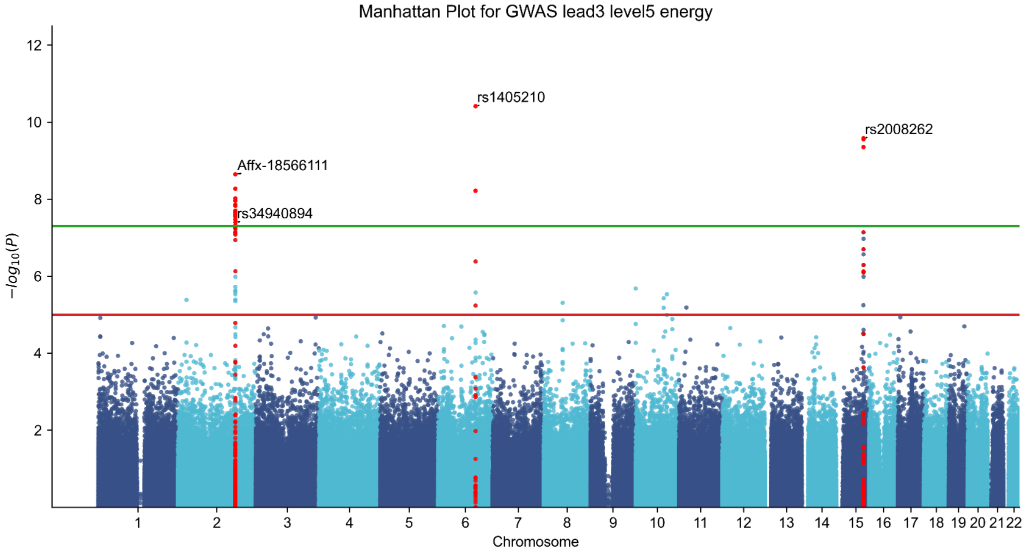

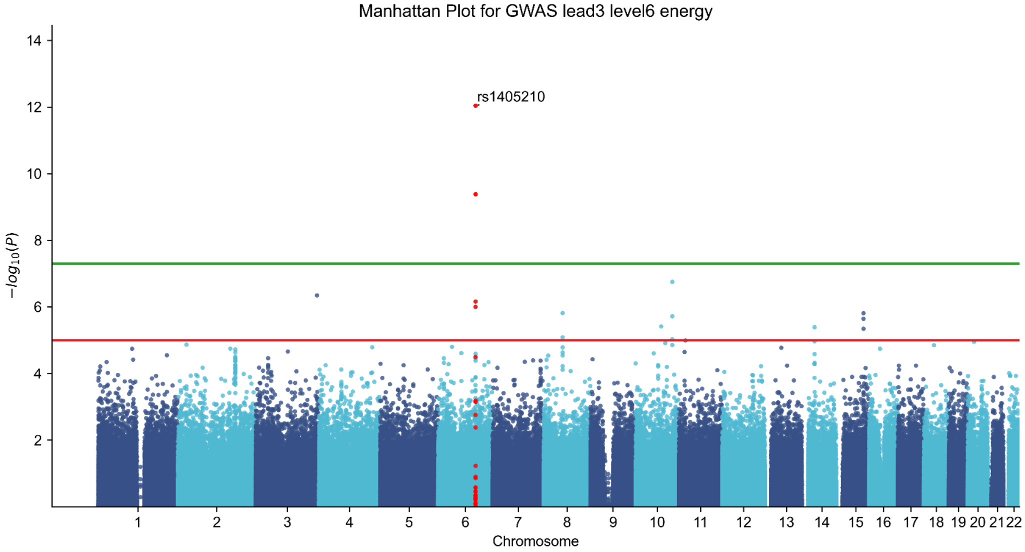

medRxiv preprint doi: https://doi.org/10.64898/2026.05.20.26353725; this version posted June 7, 2026. The copyright holder for this preprint (which was not certified by peer review) is the author/funder, who has granted medRxiv a license to display the preprint in perpetuity.
It is made available under a CC-BY 4.0 International license .

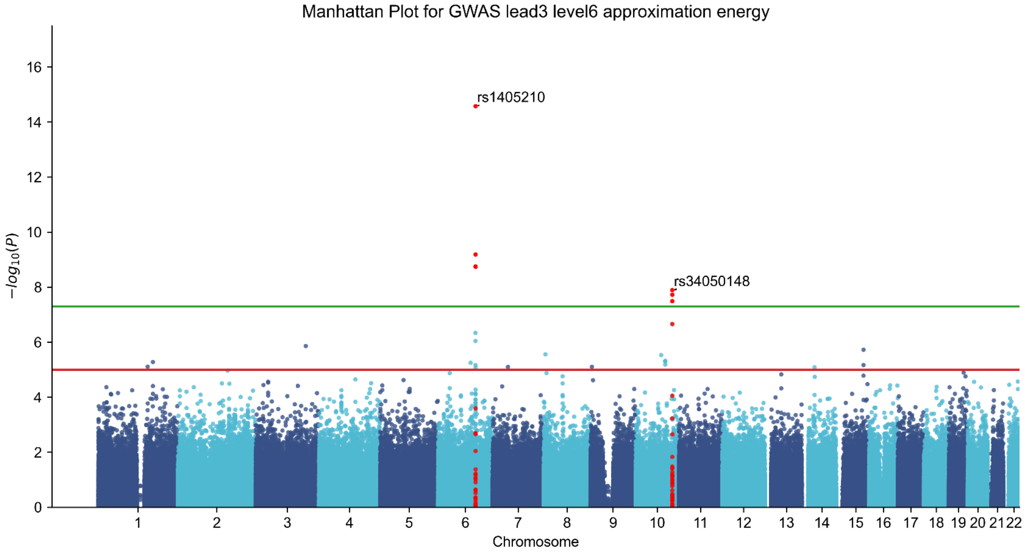

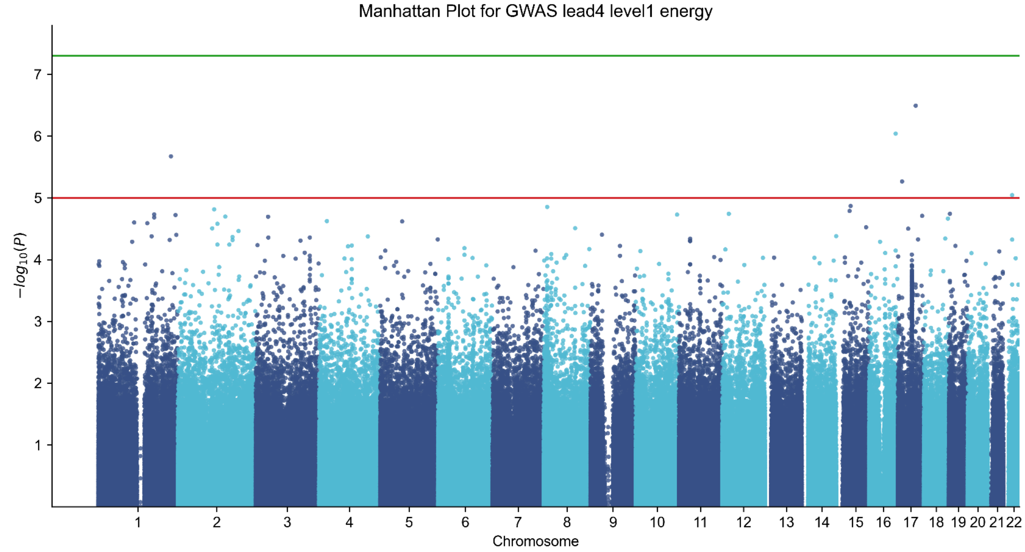

medRxiv preprint doi: https://doi.org/10.64898/2026.05.20.26353725; this version posted June 7, 2026. The copyright holder for this preprint (which was not certified by peer review) is the author/funder, who has granted medRxiv a license to display the preprint in perpetuity.
It is made available under a CC-BY 4.0 International license .

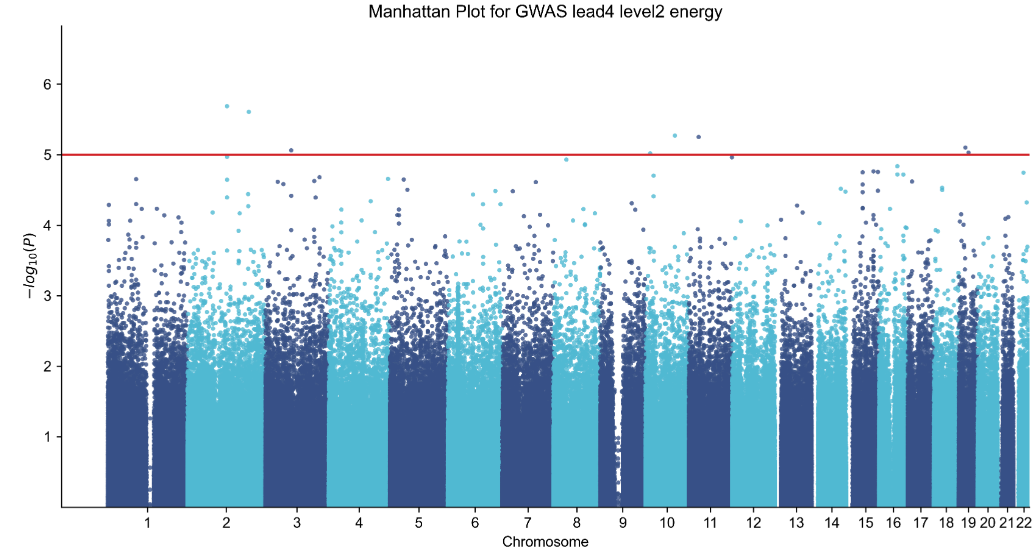

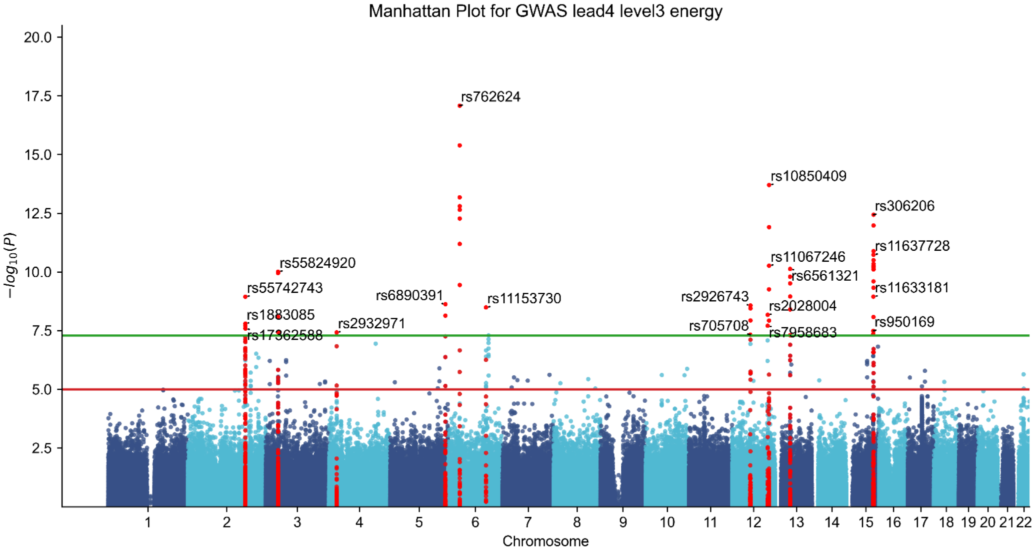

medRxiv preprint doi: https://doi.org/10.64898/2026.05.20.26353725; this version posted June 7, 2026. The copyright holder for this preprint (which was not certified by peer review) is the author/funder, who has granted medRxiv a license to display the preprint in perpetuity.
It is made available under a CC-BY 4.0 International license .

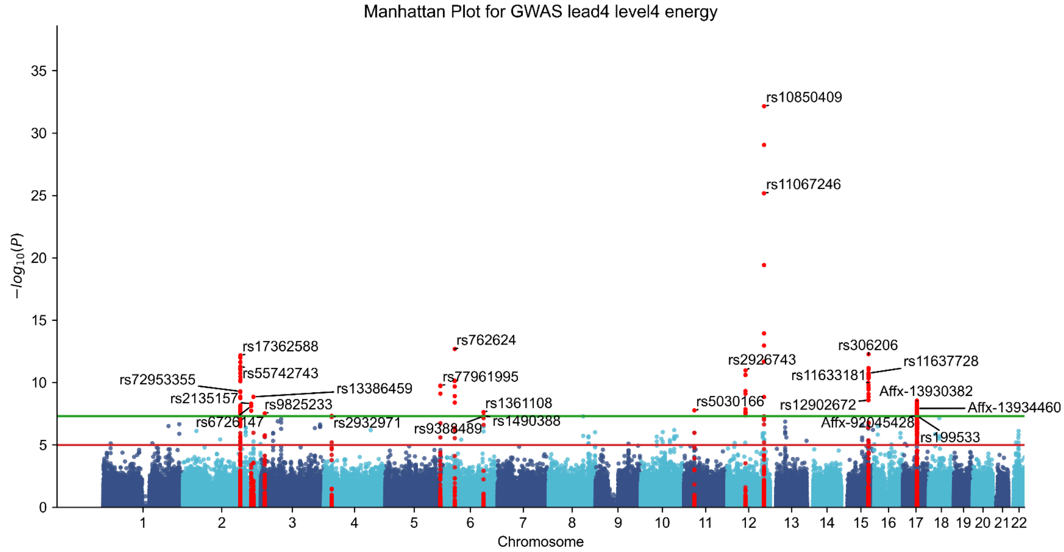

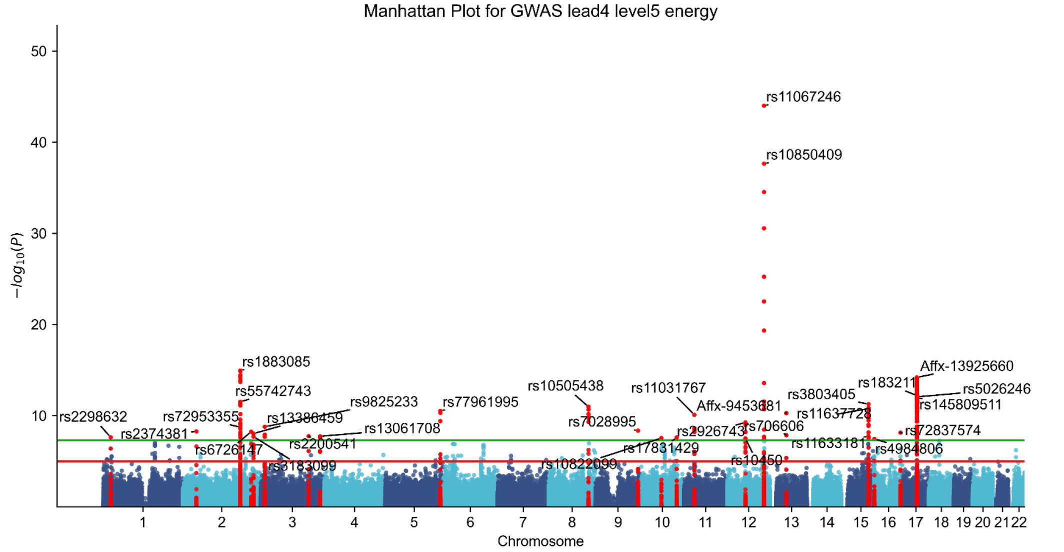

medRxiv preprint doi: https://doi.org/10.64898/2026.05.20.26353725; this version posted June 7, 2026. The copyright holder for this preprint (which was not certified by peer review) is the author/funder, who has granted medRxiv a license to display the preprint in perpetuity.
It is made available under a CC-BY 4.0 International license .

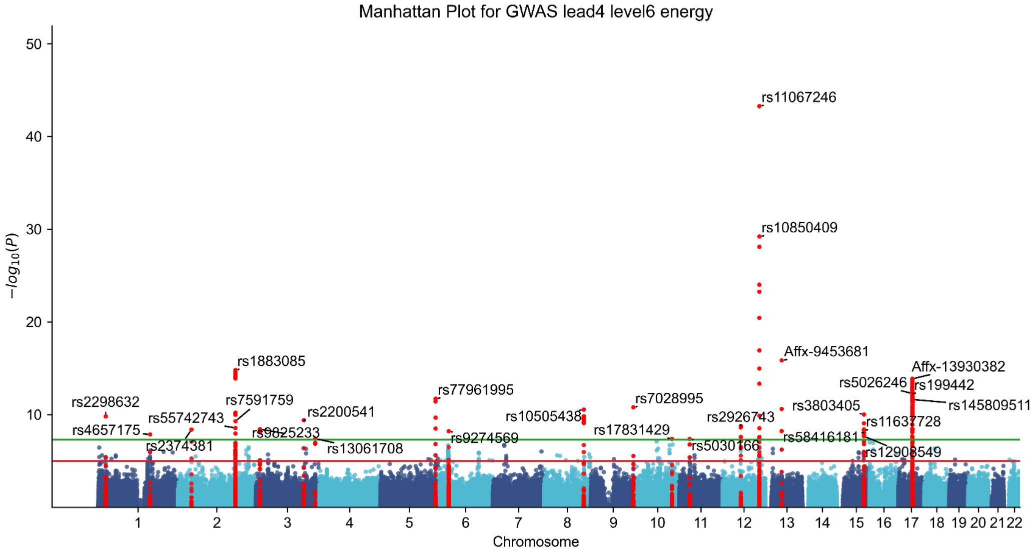

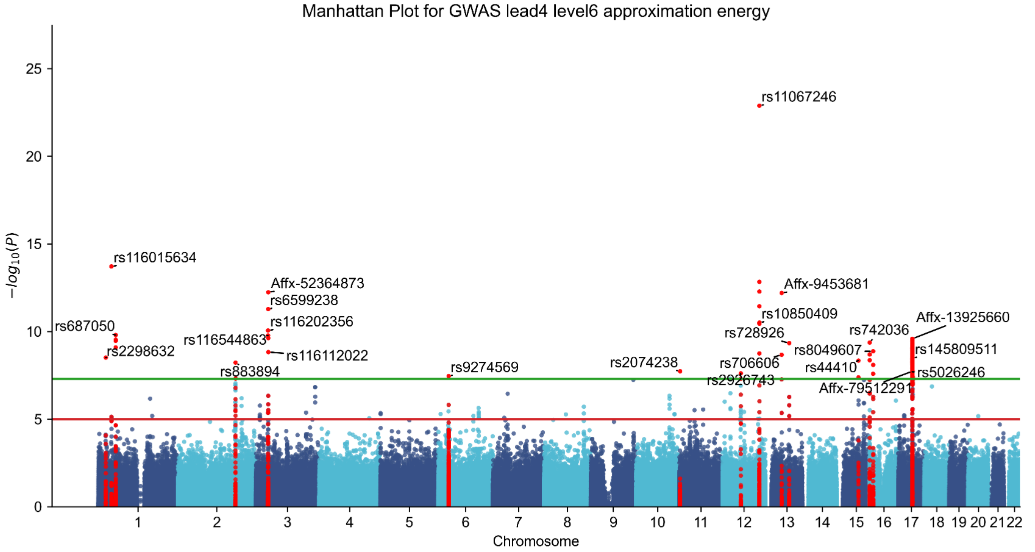

medRxiv preprint doi: https://doi.org/10.64898/2026.05.20.26353725; this version posted June 7, 2026. The copyright holder for this preprint (which was not certified by peer review) is the author/funder, who has granted medRxiv a license to display the preprint in perpetuity.
It is made available under a CC-BY 4.0 International license .

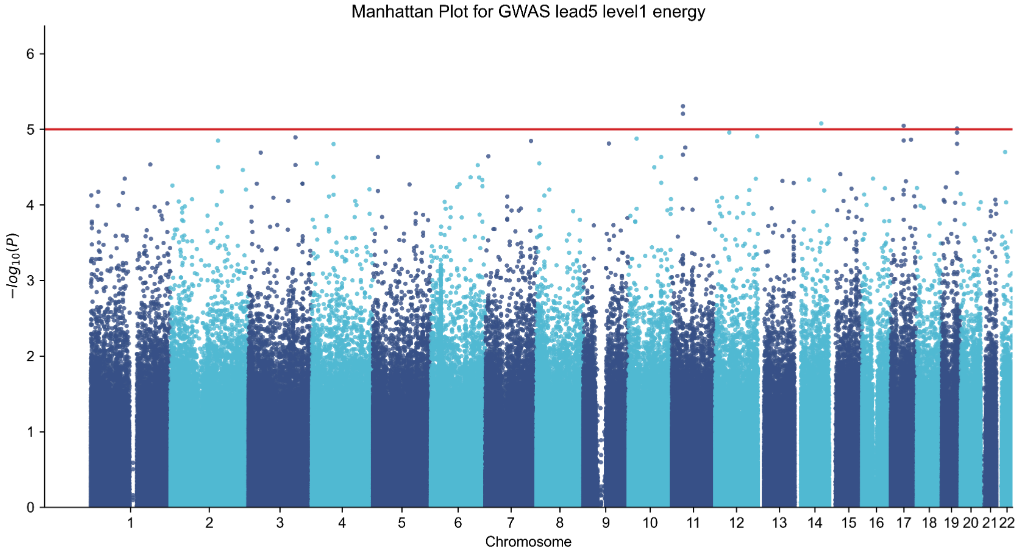

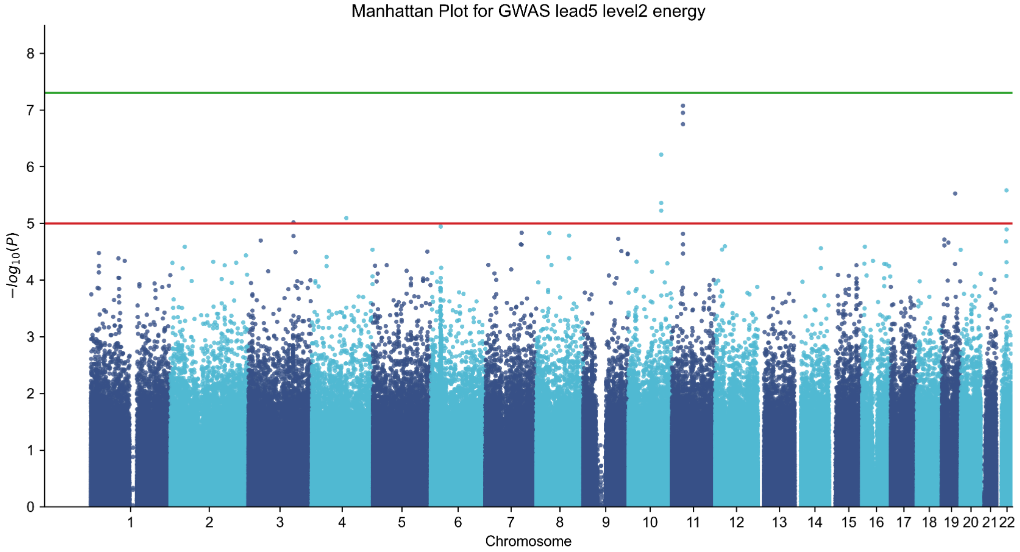

medRxiv preprint doi: https://doi.org/10.64898/2026.05.20.26353725; this version posted June 7, 2026. The copyright holder for this preprint (which was not certified by peer review) is the author/funder, who has granted medRxiv a license to display the preprint in perpetuity.
It is made available under a CC-BY 4.0 International license .

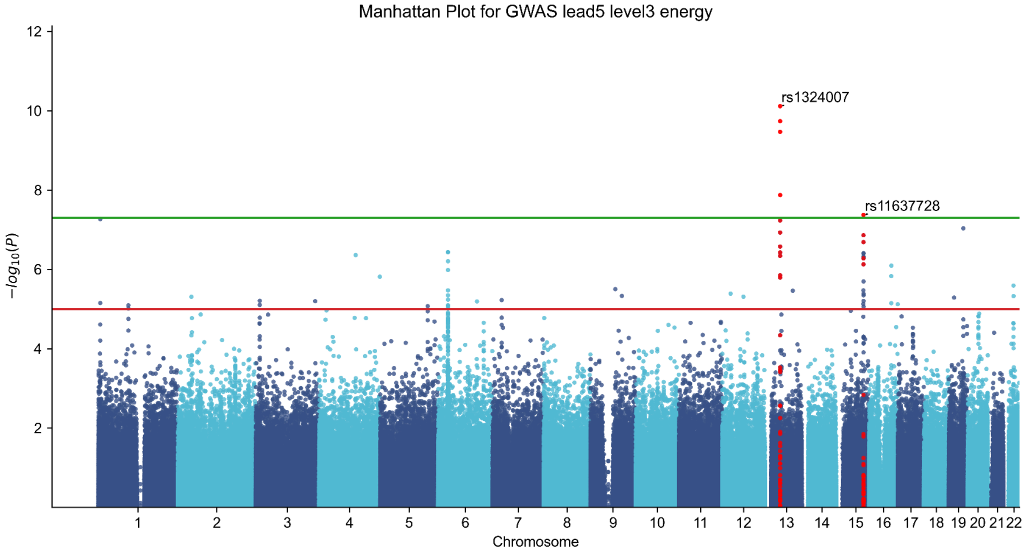

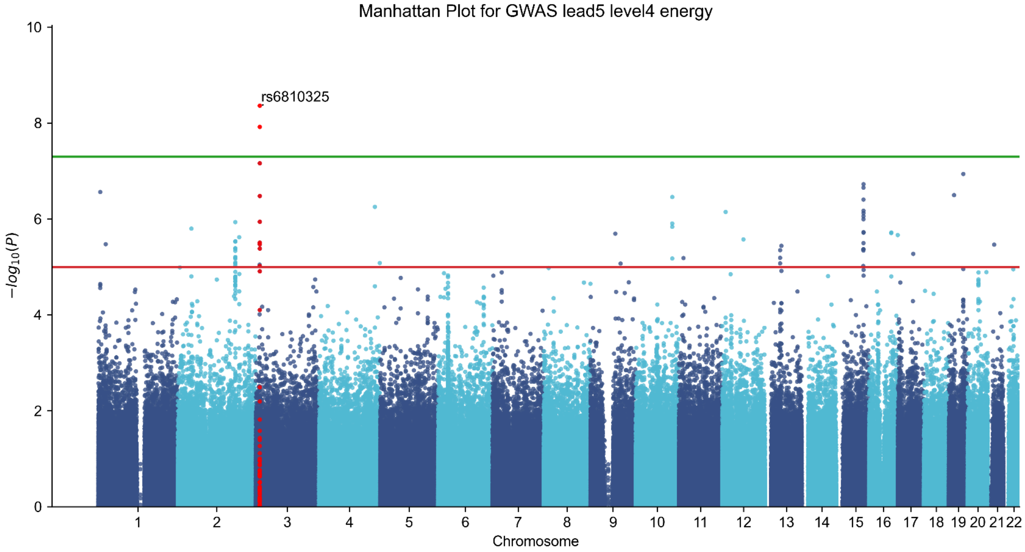

medRxiv preprint doi: https://doi.org/10.64898/2026.05.20.26353725; this version posted June 7, 2026. The copyright holder for this preprint
(which was not certified by peer review) is the author/funder, who has granted medRxiv a license to display the preprint in perpetuity.
It is made available under a CC-BY 4.0 International license .

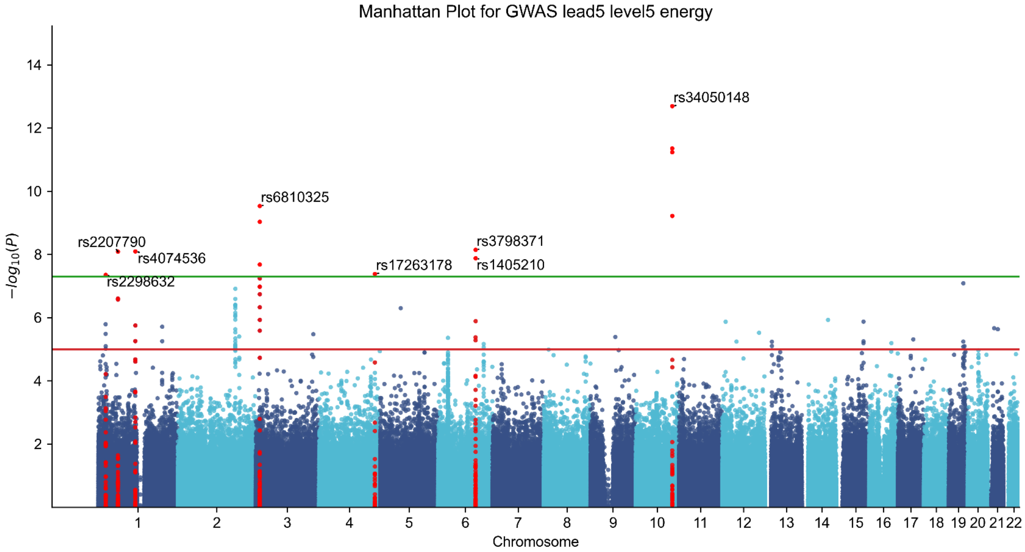

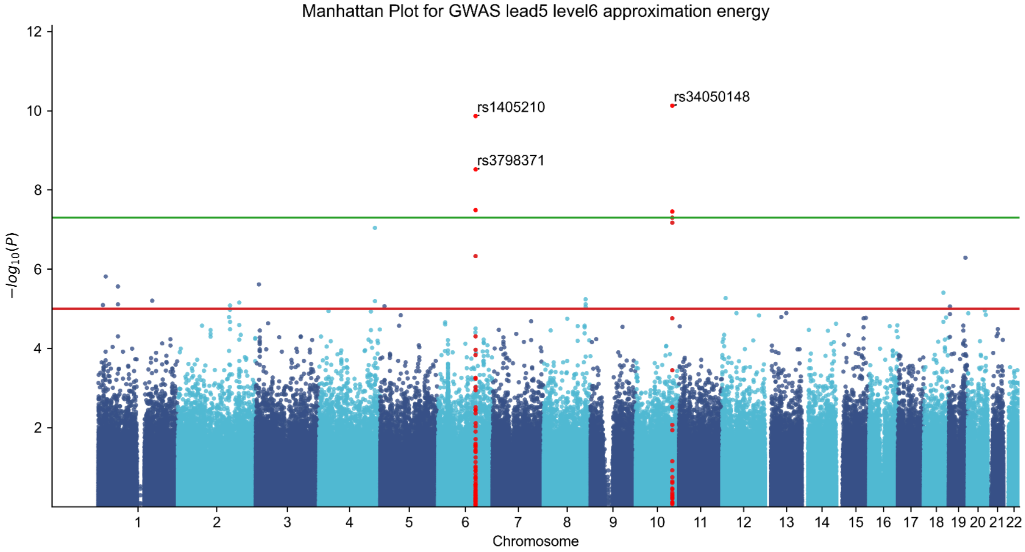

medRxiv preprint doi: https://doi.org/10.64898/2026.05.20.26353725; this version posted June 7, 2026. The copyright holder for this preprint (which was not certified by peer review) is the author/funder, who has granted medRxiv a license to display the preprint in perpetuity.
It is made available under a CC-BY 4.0 International license .

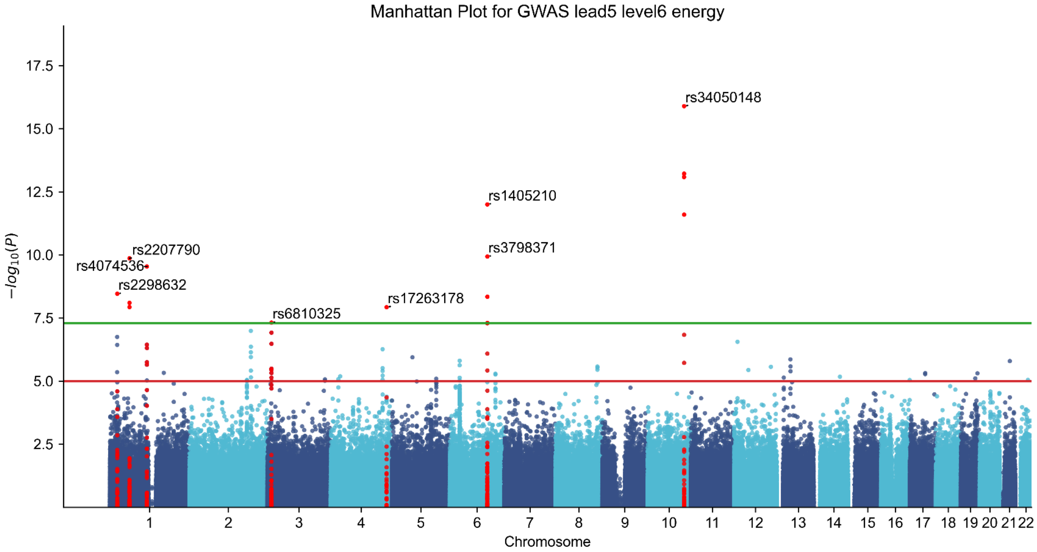

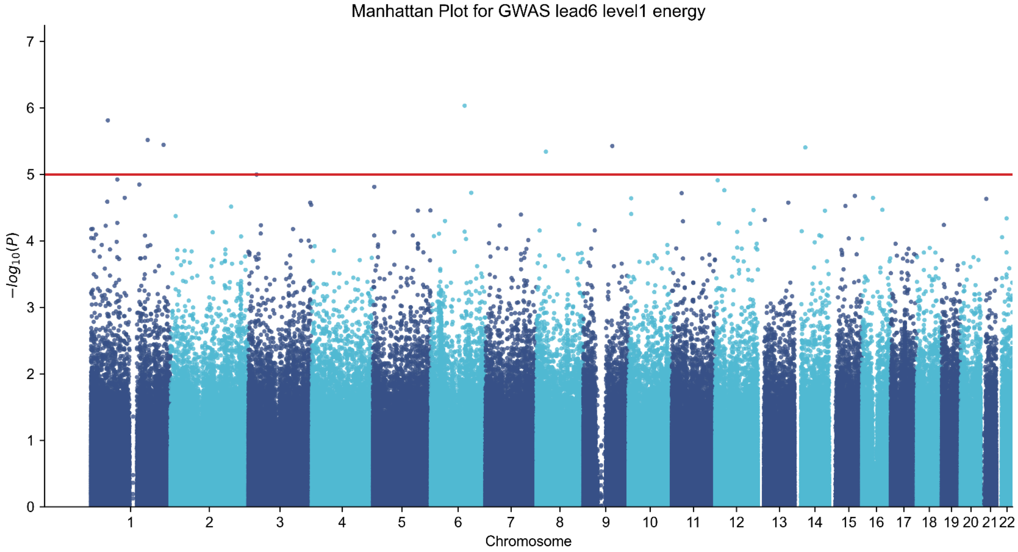

medRxiv preprint doi: https://doi.org/10.64898/2026.05.20.26353725; this version posted June 7, 2026. The copyright holder for this preprint (which was not certified by peer review) is the author/funder, who has granted medRxiv a license to display the preprint in perpetuity.
It is made available under a CC-BY 4.0 International license .

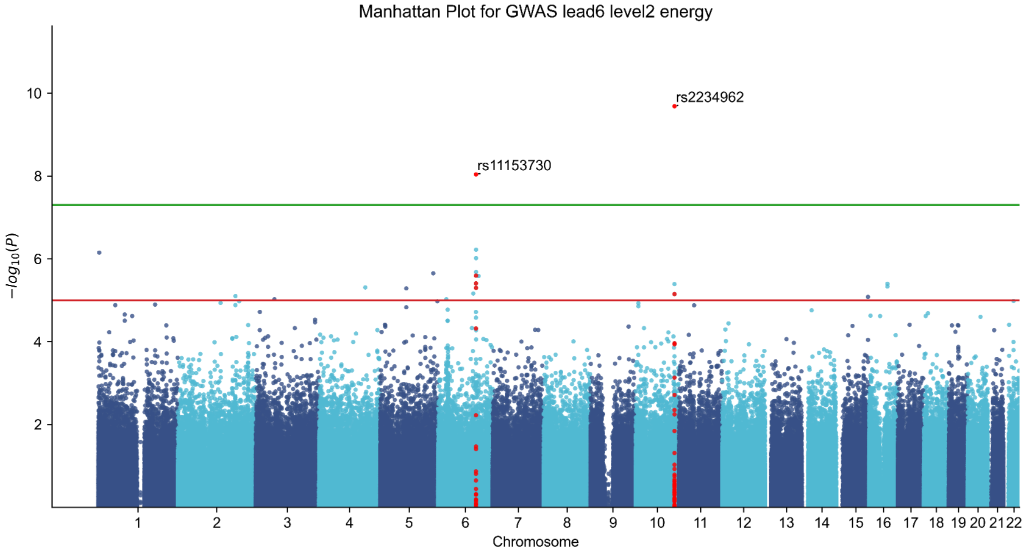

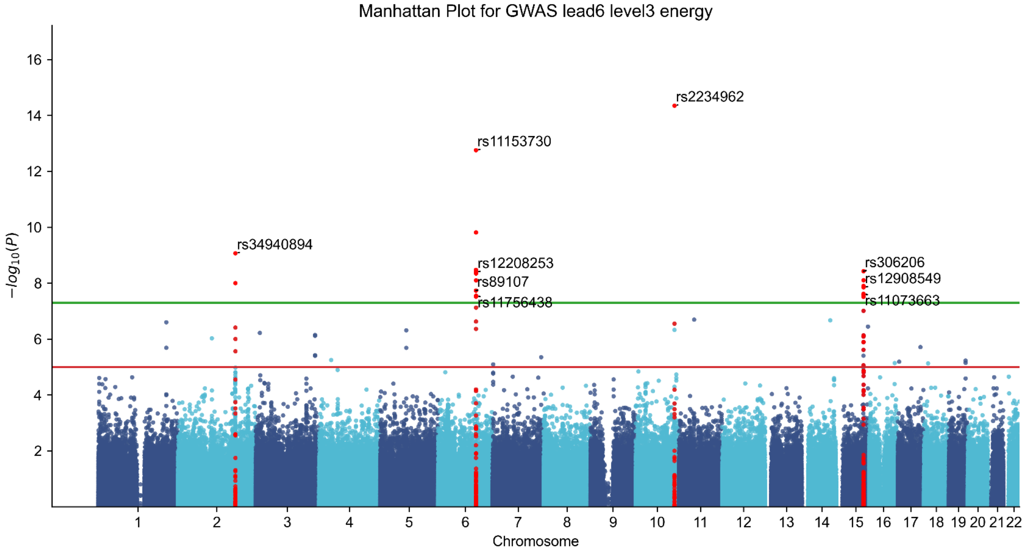

medRxiv preprint doi: https://doi.org/10.64898/2026.05.20.26353725; this version posted June 7, 2026. The copyright holder for this preprint (which was not certified by peer review) is the author/funder, who has granted medRxiv a license to display the preprint in perpetuity.
It is made available under a CC-BY 4.0 International license .

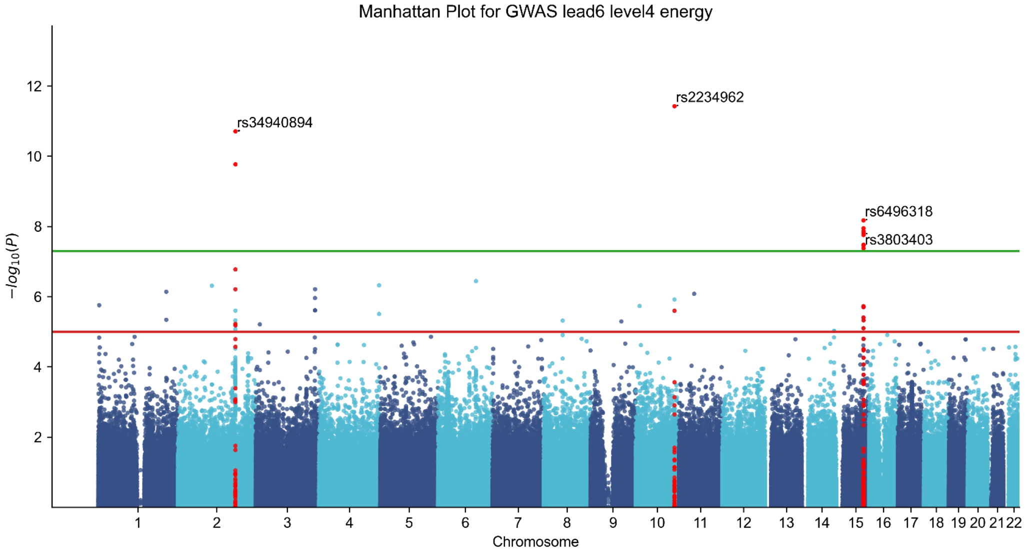

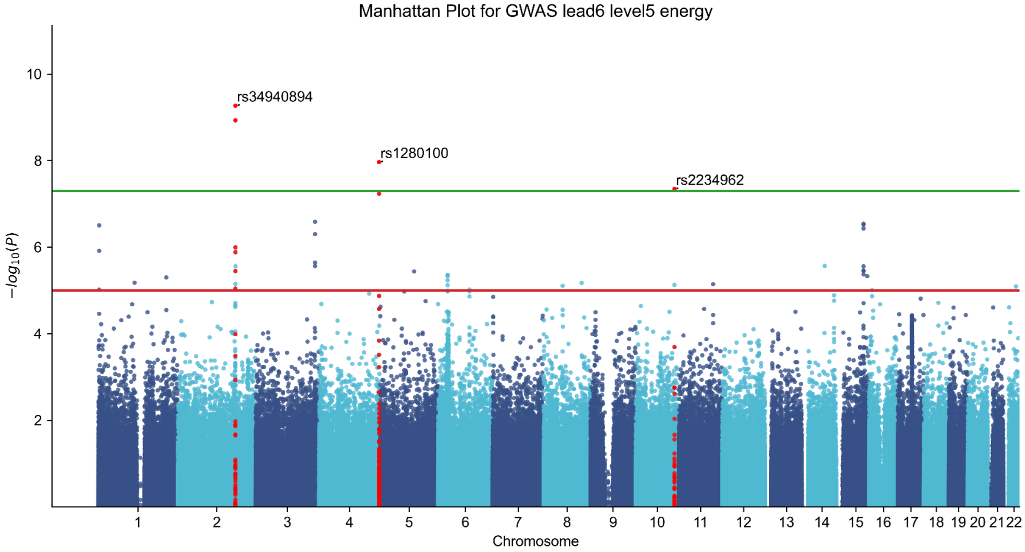

medRxiv preprint doi: https://doi.org/10.64898/2026.05.20.26353725; this version posted June 7, 2026. The copyright holder for this preprint
(which was not certified by peer review) is the author/funder, who has granted medRxiv a license to display the preprint in perpetuity.
It is made available under a CC-BY 4.0 International license .

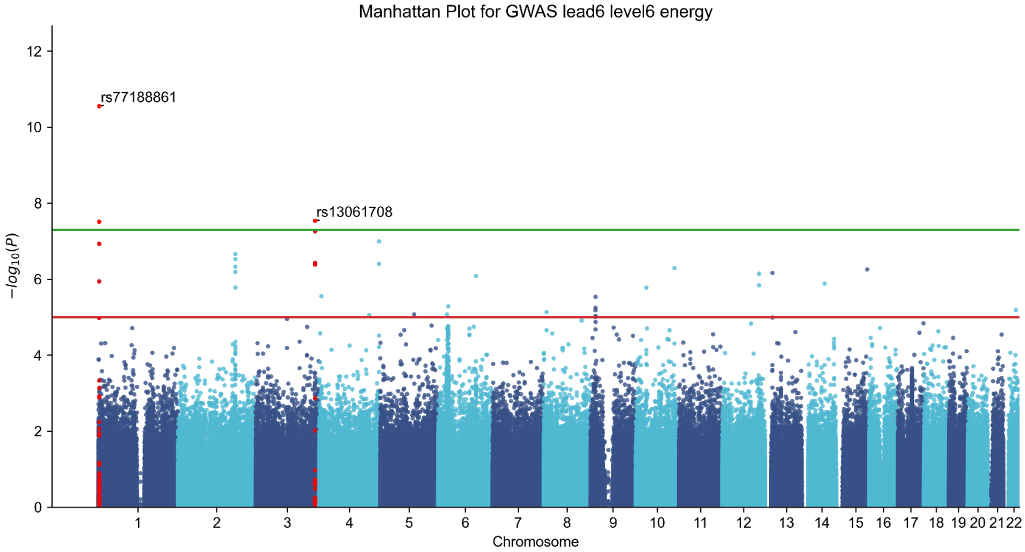

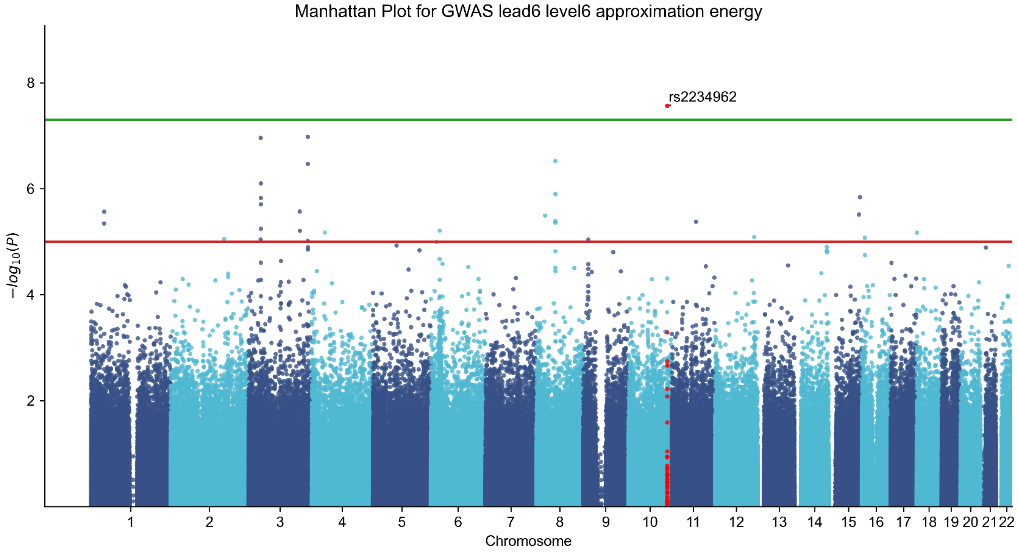

medRxiv preprint doi: https://doi.org/10.64898/2026.05.20.26353725; this version posted June 7, 2026. The copyright holder for this preprint (which was not certified by peer review) is the author/funder, who has granted medRxiv a license to display the preprint in perpetuity.
It is made available under a CC-BY 4.0 International license .

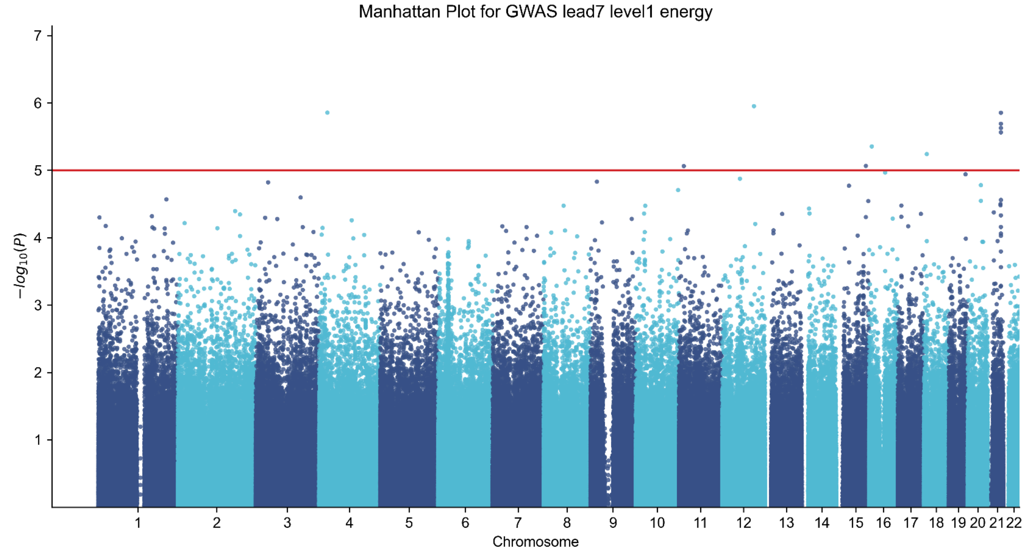

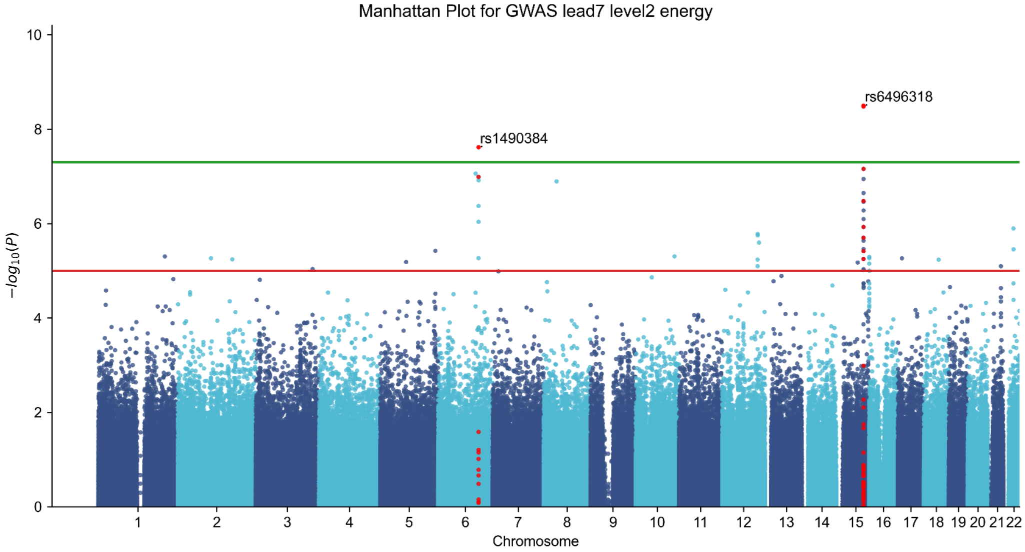

medRxiv preprint doi: https://doi.org/10.64898/2026.05.20.26353725; this version posted June 7, 2026. The copyright holder for this preprint (which was not certified by peer review) is the author/funder, who has granted medRxiv a license to display the preprint in perpetuity.
It is made available under a CC-BY 4.0 International license .

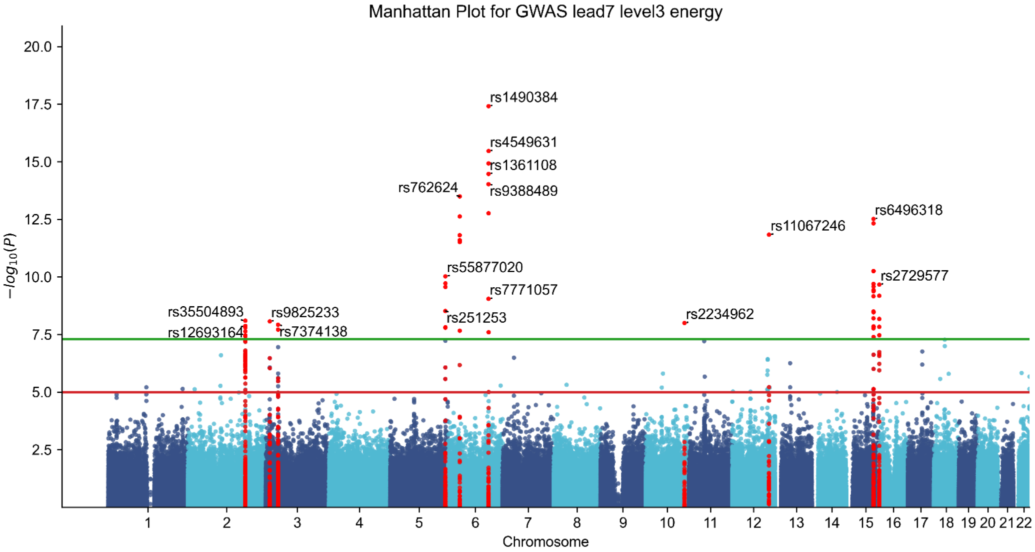

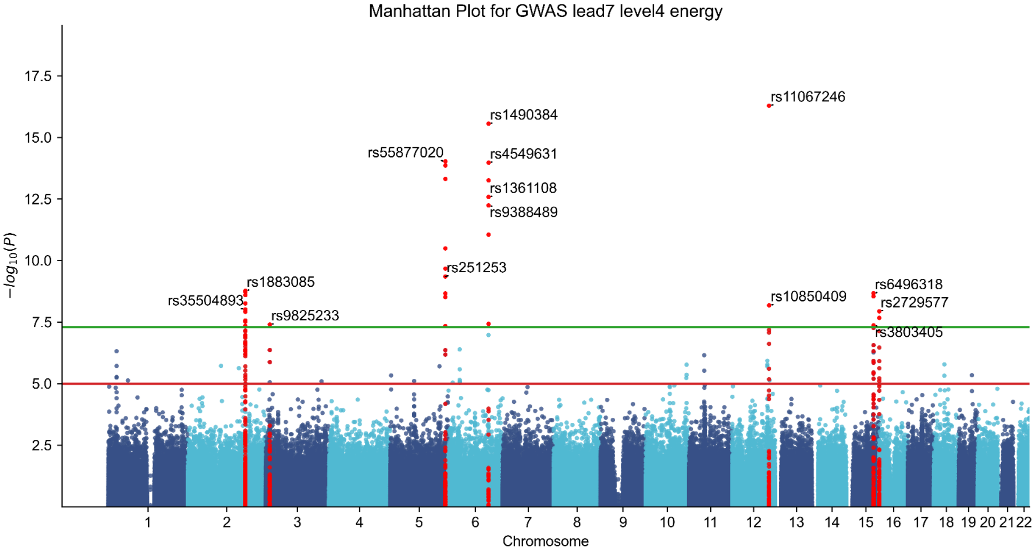

medRxiv preprint doi: https://doi.org/10.64898/2026.05.20.26353725; this version posted June 7, 2026. The copyright holder for this preprint (which was not certified by peer review) is the author/funder, who has granted medRxiv a license to display the preprint in perpetuity.
It is made available under a CC-BY 4.0 International license .

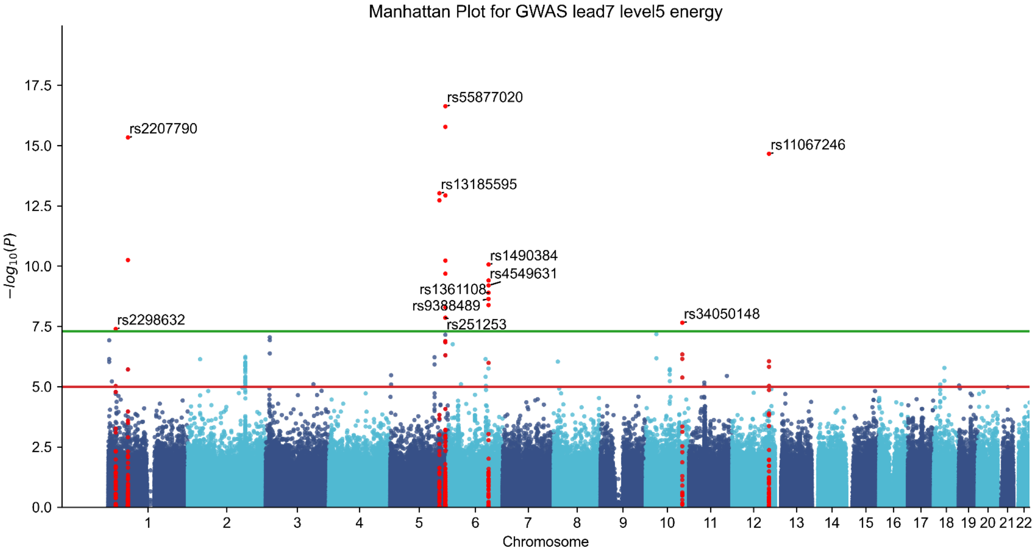

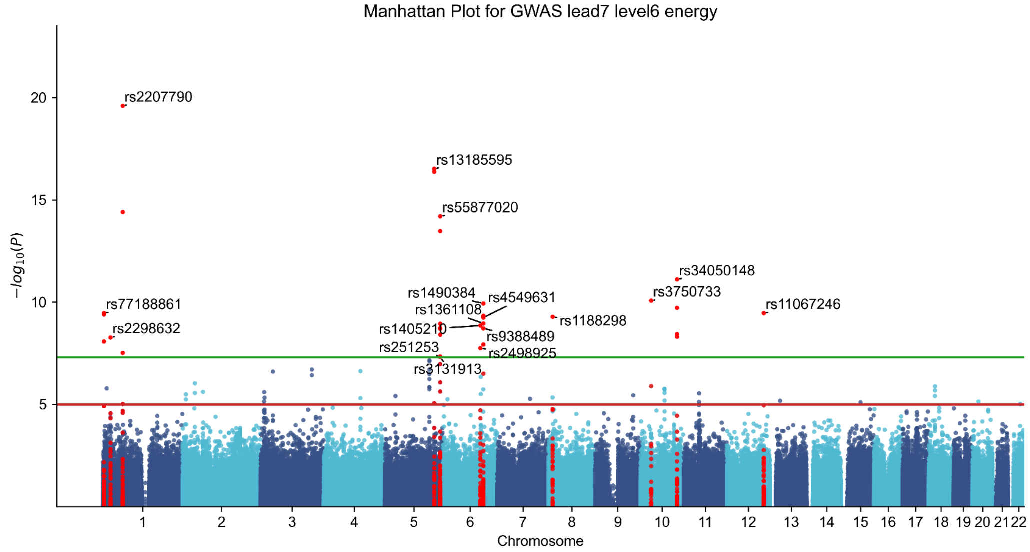

medRxiv preprint doi: https://doi.org/10.64898/2026.05.20.26353725; this version posted June 7, 2026. The copyright holder for this preprint (which was not certified by peer review) is the author/funder, who has granted medRxiv a license to display the preprint in perpetuity.
It is made available under a CC-BY 4.0 International license .

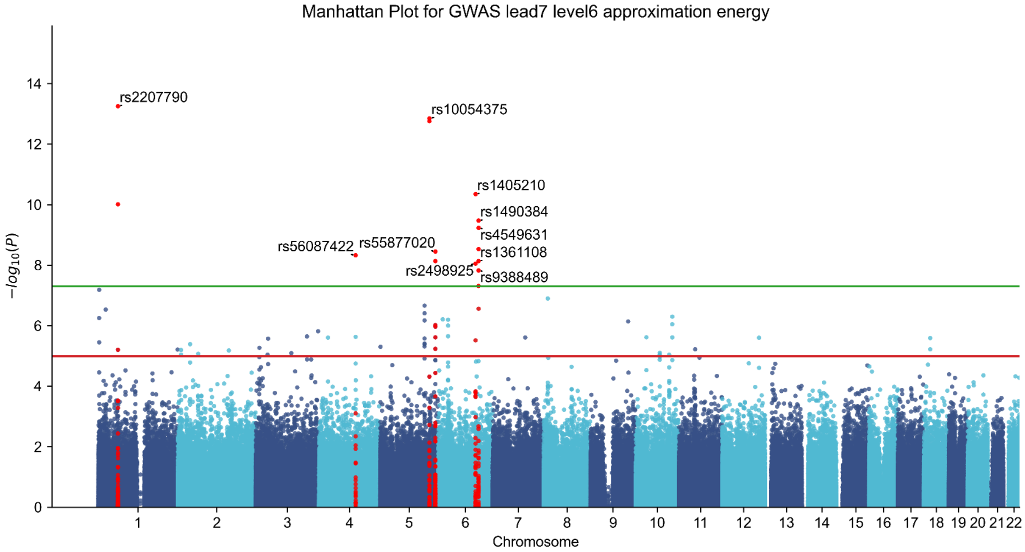

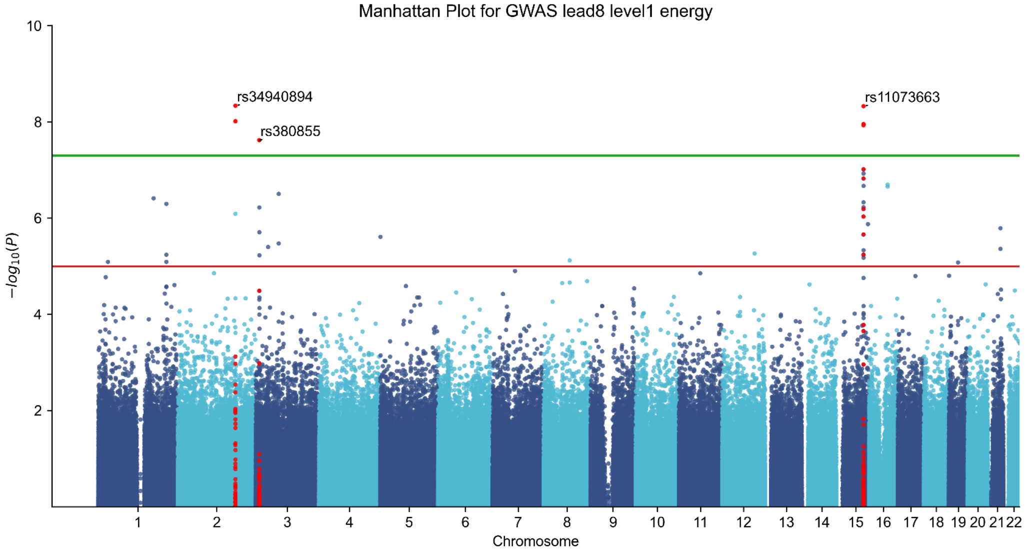

medRxiv preprint doi: https://doi.org/10.64898/2026.05.20.26353725; this version posted June 7, 2026. The copyright holder for this preprint (which was not certified by peer review) is the author/funder, who has granted medRxiv a license to display the preprint in perpetuity.
It is made available under a CC-BY 4.0 International license .

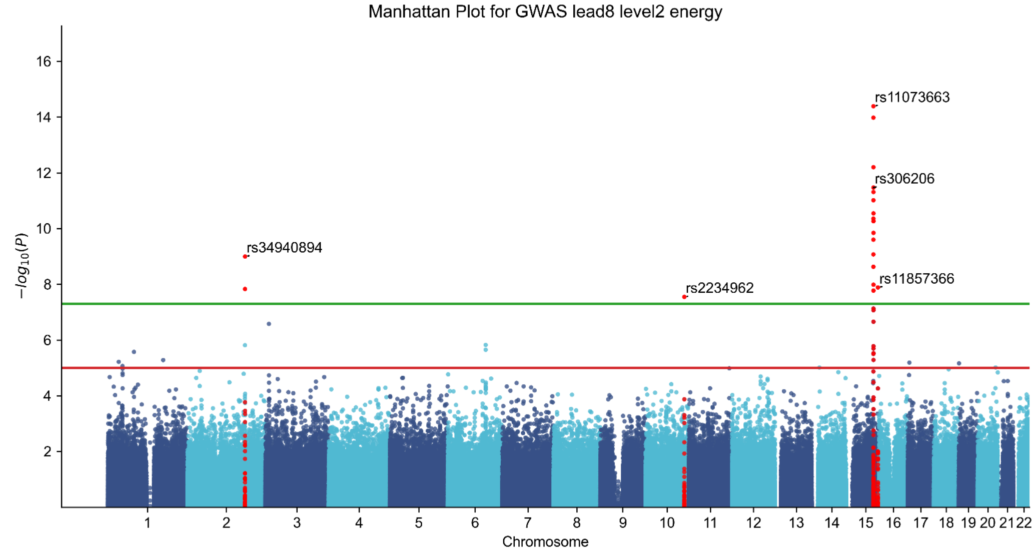

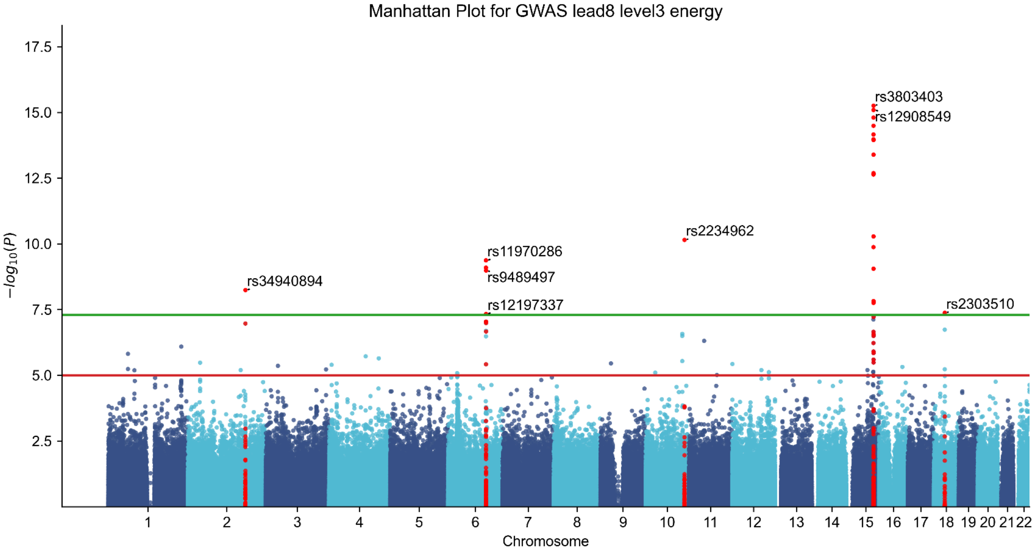

medRxiv preprint doi: https://doi.org/10.64898/2026.05.20.26353725; this version posted June 7, 2026. The copyright holder for this preprint (which was not certified by peer review) is the author/funder, who has granted medRxiv a license to display the preprint in perpetuity.
It is made available under a CC-BY 4.0 International license .

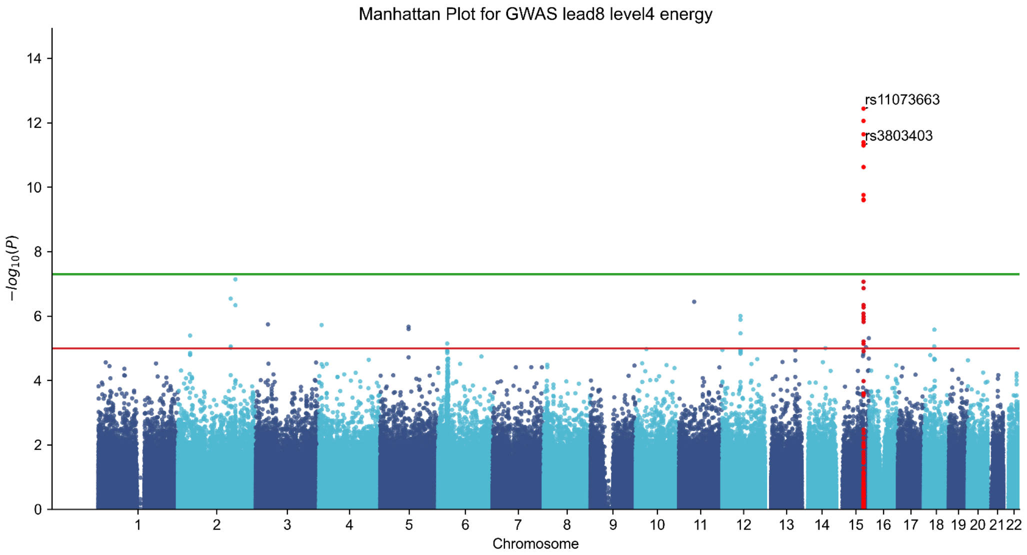

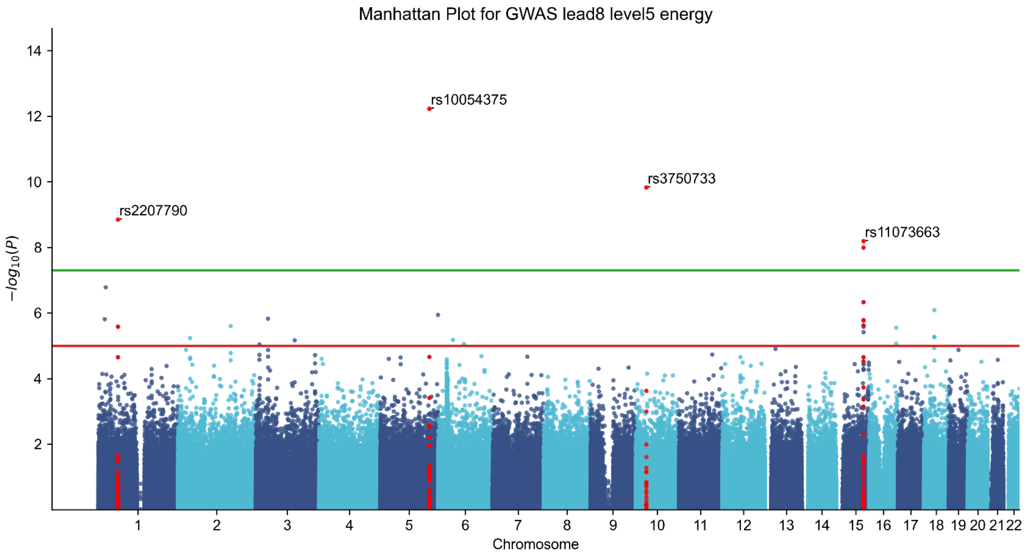

medRxiv preprint doi: https://doi.org/10.64898/2026.05.20.26353725; this version posted June 7, 2026. The copyright holder for this preprint (which was not certified by peer review) is the author/funder, who has granted medRxiv a license to display the preprint in perpetuity.
It is made available under a CC-BY 4.0 International license .

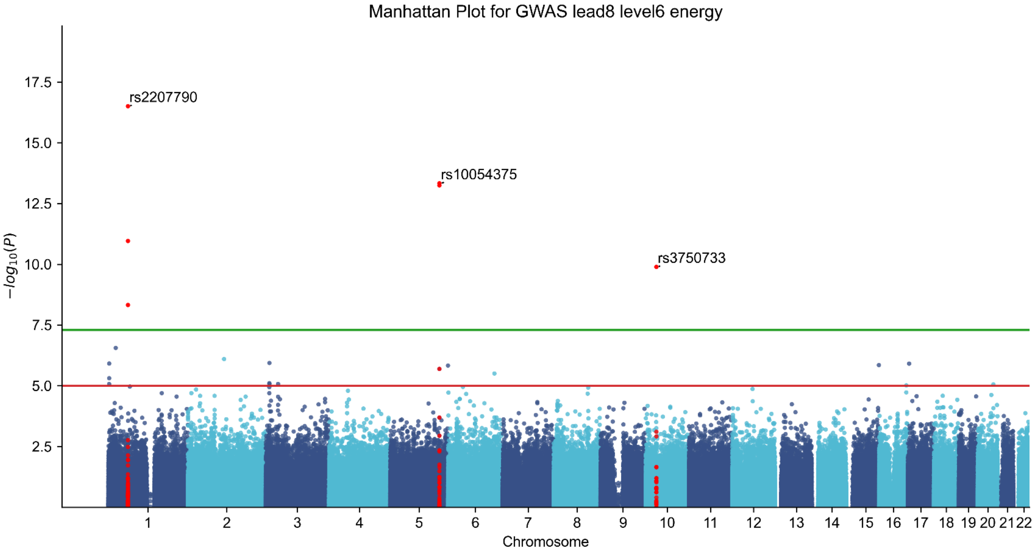

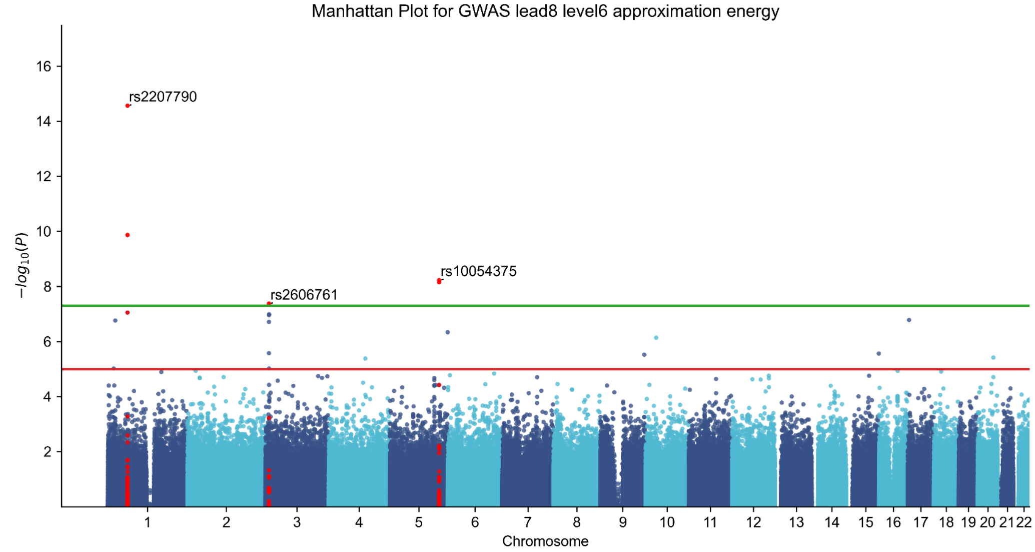

medRxiv preprint doi: https://doi.org/10.64898/2026.05.20.26353725; this version posted June 7, 2026. The copyright holder for this preprint (which was not certified by peer review) is the author/funder, who has granted medRxiv a license to display the preprint in perpetuity.
It is made available under a CC-BY 4.0 International license .

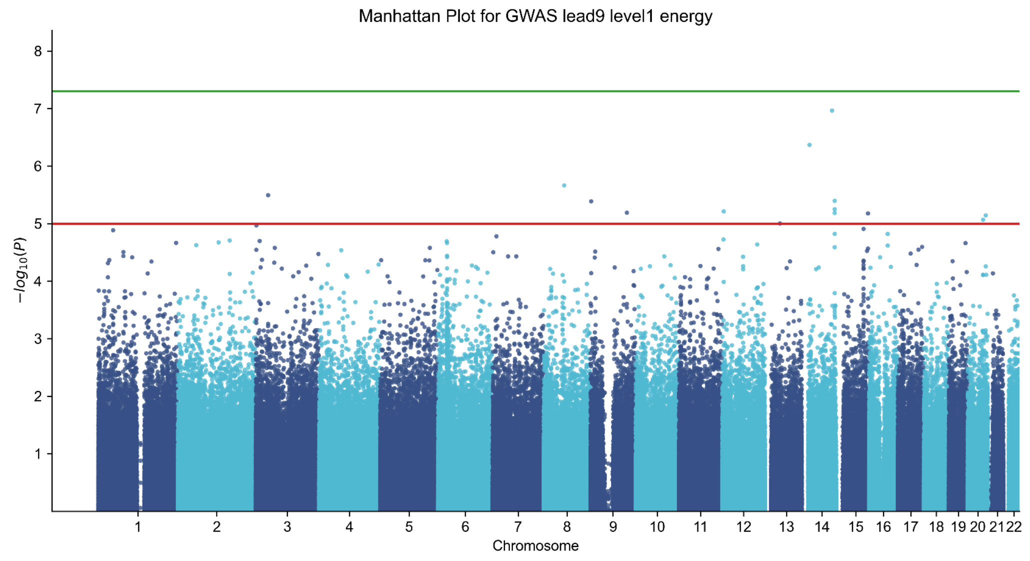

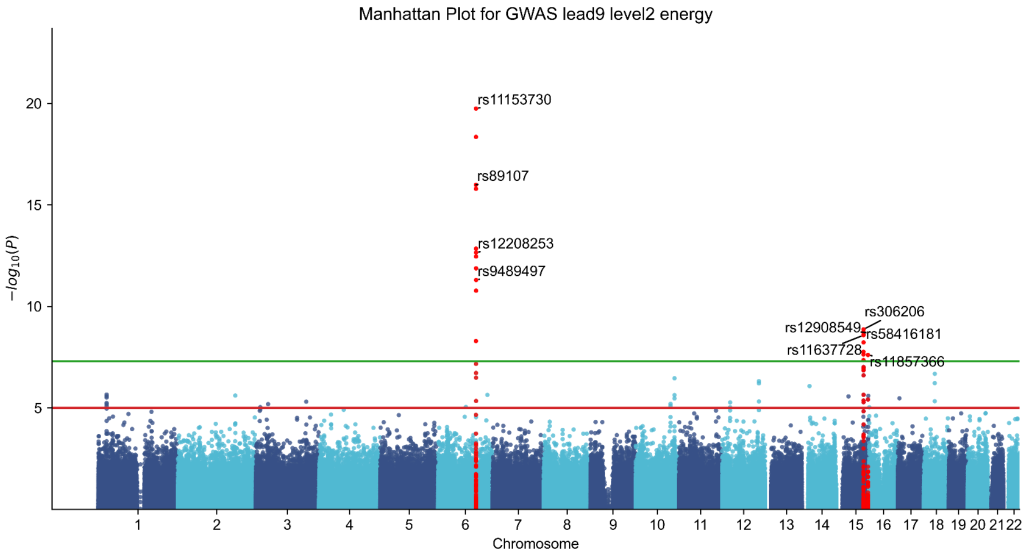

medRxiv preprint doi: https://doi.org/10.64898/2026.05.20.26353725; this version posted June 7, 2026. The copyright holder for this preprint (which was not certified by peer review) is the author/funder, who has granted medRxiv a license to display the preprint in perpetuity.
It is made available under a CC-BY 4.0 International license .

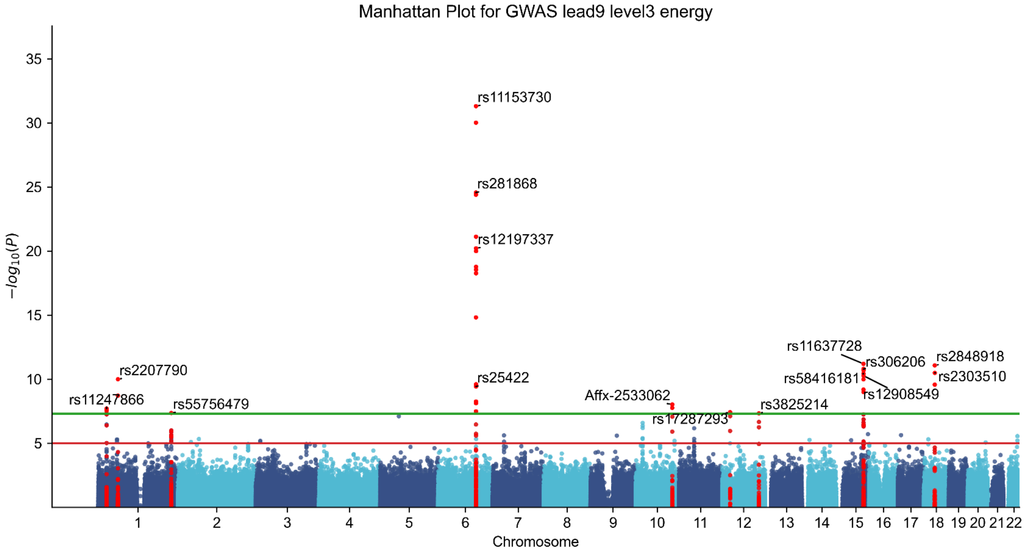

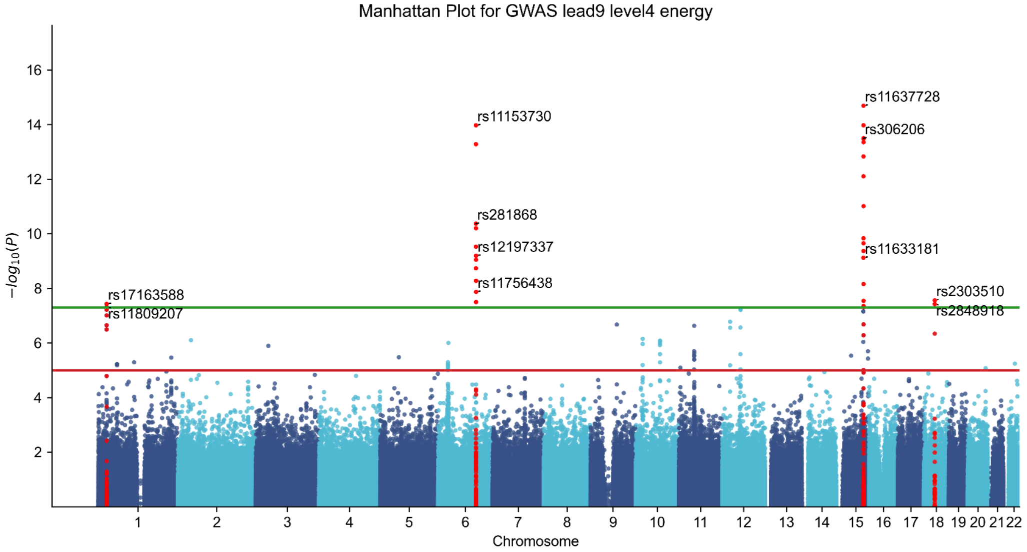

medRxiv preprint doi: https://doi.org/10.64898/2026.05.20.26353725; this version posted June 7, 2026. The copyright holder for this preprint (which was not certified by peer review) is the author/funder, who has granted medRxiv a license to display the preprint in perpetuity.
It is made available under a CC-BY 4.0 International license .

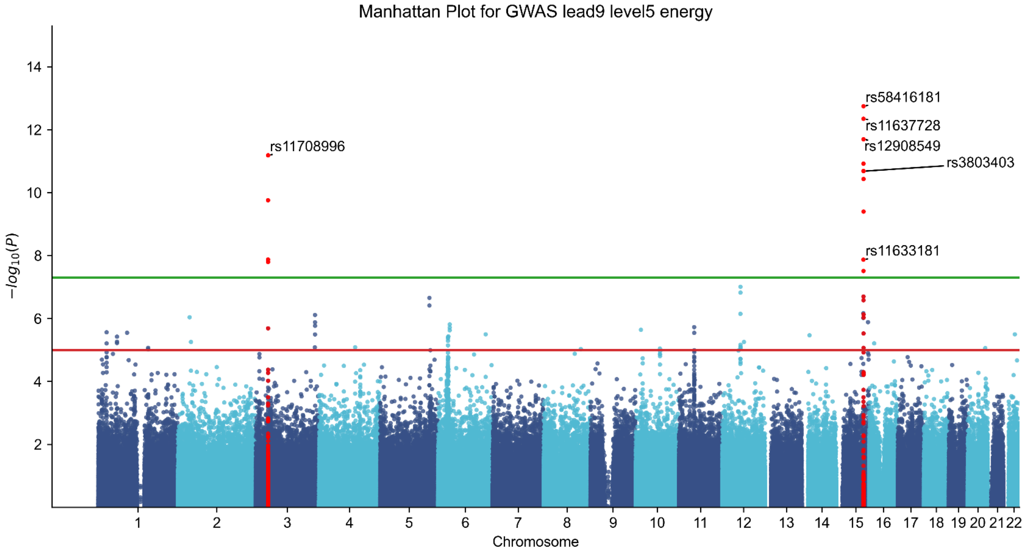

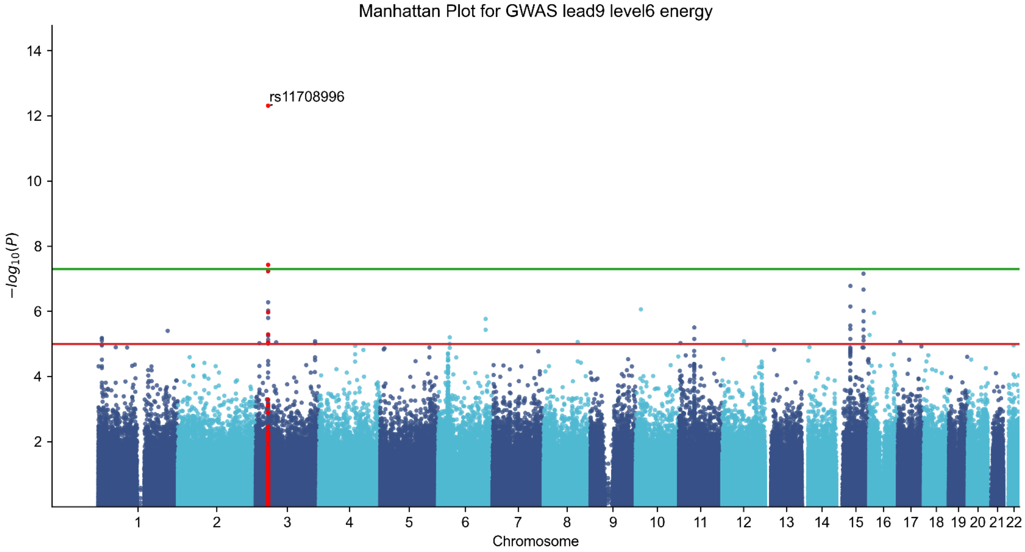

medRxiv preprint doi: https://doi.org/10.64898/2026.05.20.26353725; this version posted June 7, 2026. The copyright holder for this preprint (which was not certified by peer review) is the author/funder, who has granted medRxiv a license to display the preprint in perpetuity.
It is made available under a CC-BY 4.0 International license .

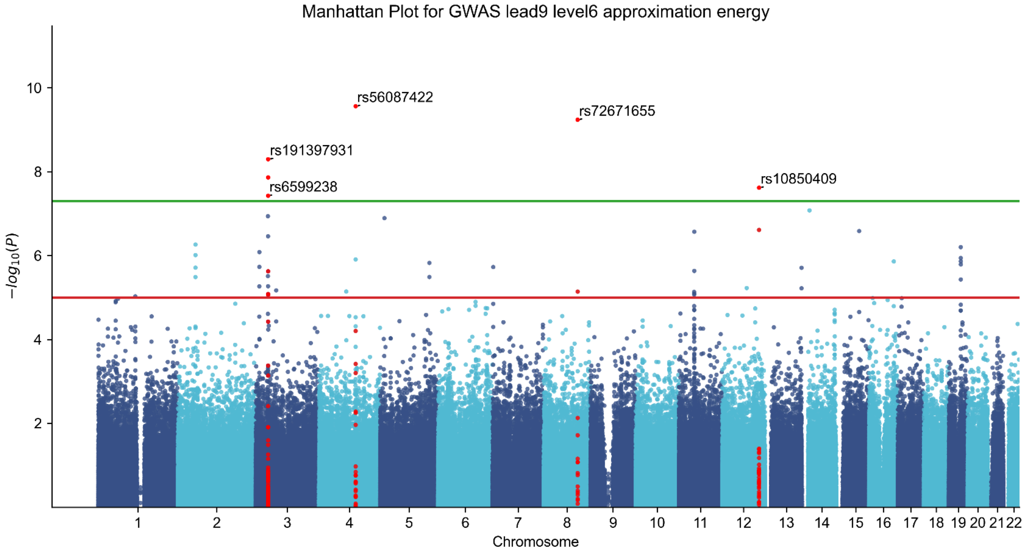

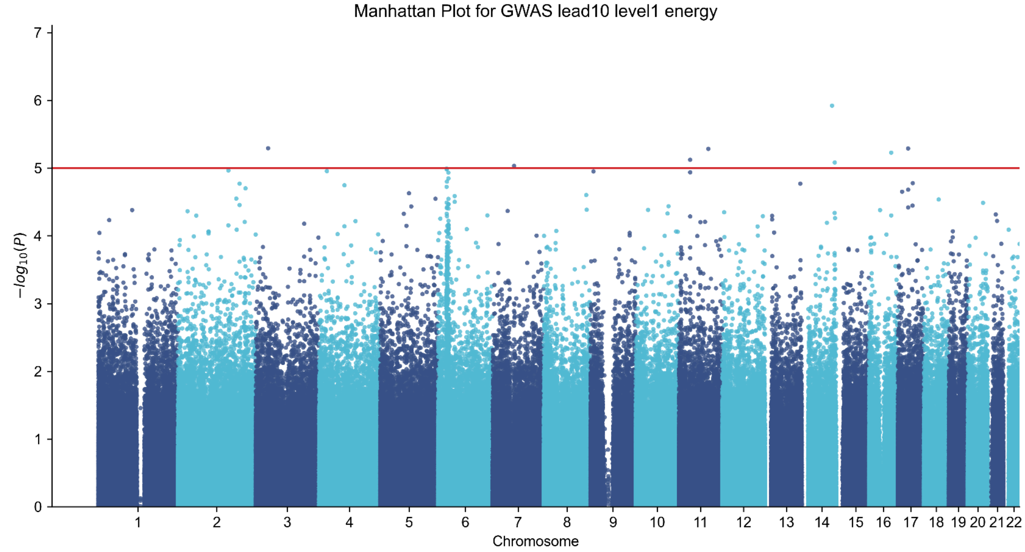

medRxiv preprint doi: https://doi.org/10.64898/2026.05.20.26353725; this version posted June 7, 2026. The copyright holder for this preprint (which was not certified by peer review) is the author/funder, who has granted medRxiv a license to display the preprint in perpetuity.
It is made available under a CC-BY 4.0 International license .

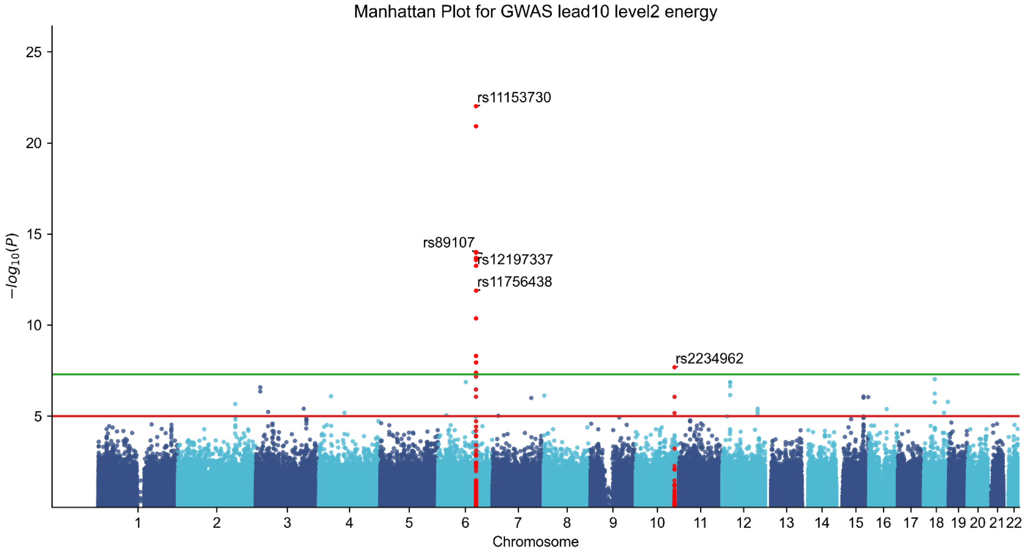

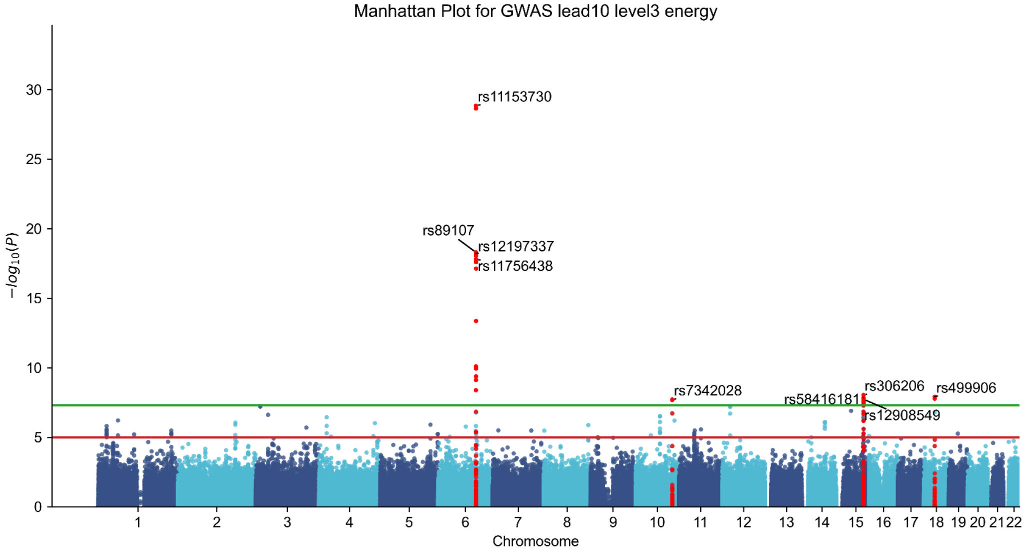

medRxiv preprint doi: https://doi.org/10.64898/2026.05.20.26353725; this version posted June 7, 2026. The copyright holder for this preprint
(which was not certified by peer review) is the author/funder, who has granted medRxiv a license to display the preprint in perpetuity.
It is made available under a CC-BY 4.0 International license .

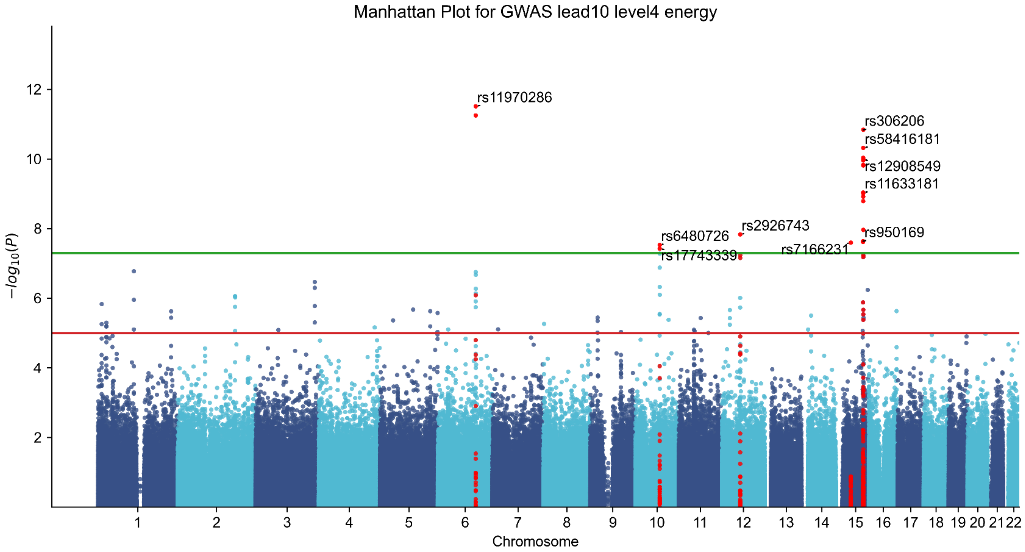

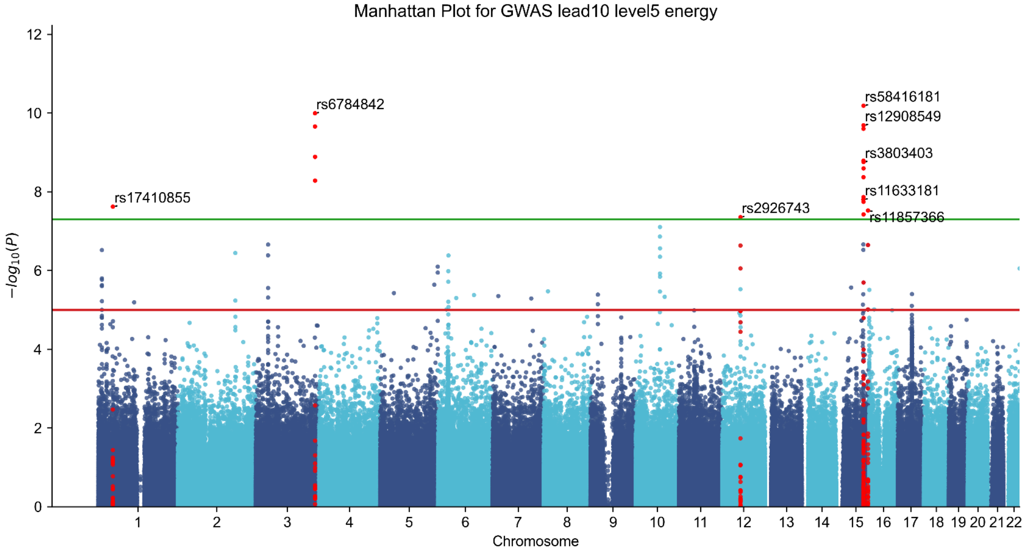

medRxiv preprint doi: https://doi.org/10.64898/2026.05.20.26353725; this version posted June 7, 2026. The copyright holder for this preprint (which was not certified by peer review) is the author/funder, who has granted medRxiv a license to display the preprint in perpetuity.
It is made available under a CC-BY 4.0 International license .

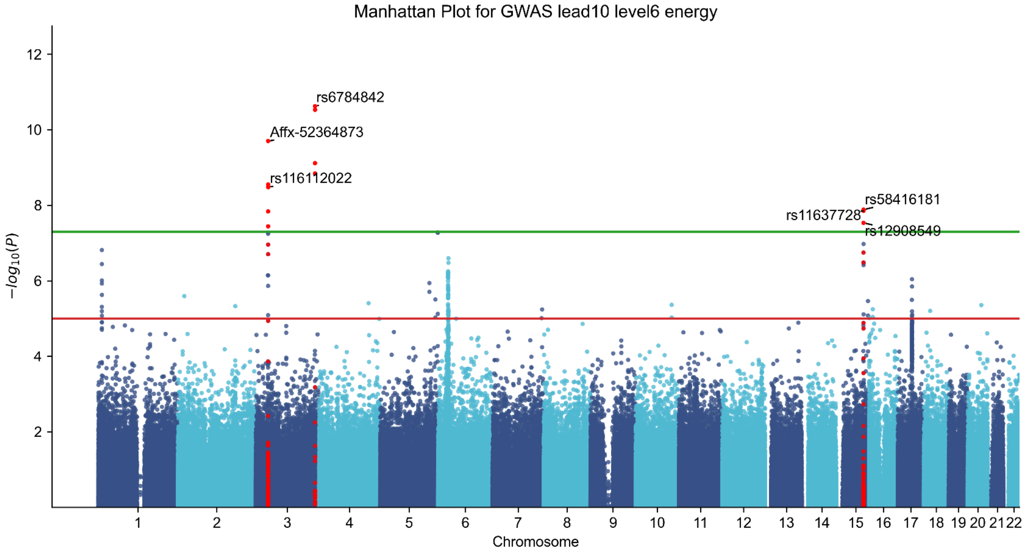

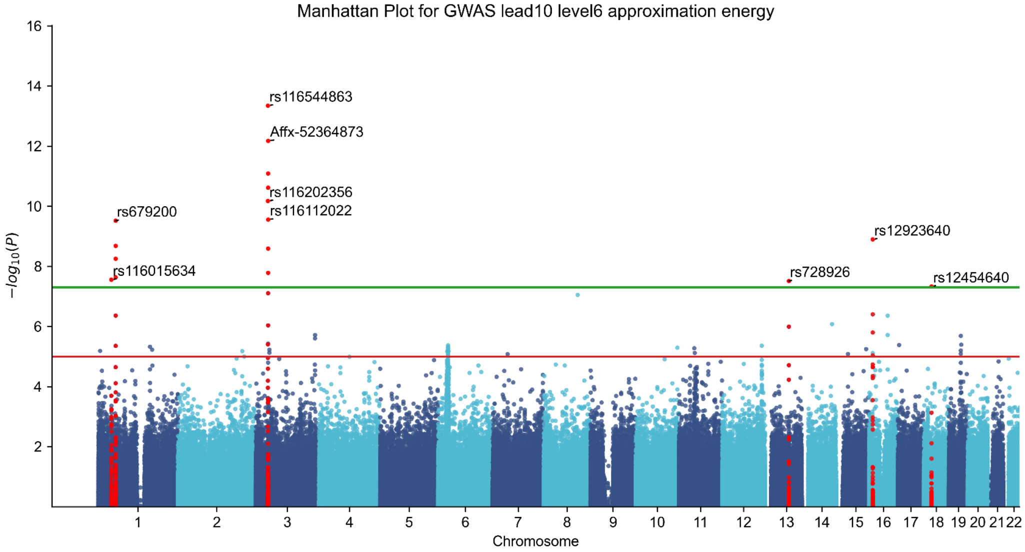

medRxiv preprint doi: https://doi.org/10.64898/2026.05.20.26353725; this version posted June 7, 2026. The copyright holder for this preprint (which was not certified by peer review) is the author/funder, who has granted medRxiv a license to display the preprint in perpetuity.
It is made available under a CC-BY 4.0 International license .

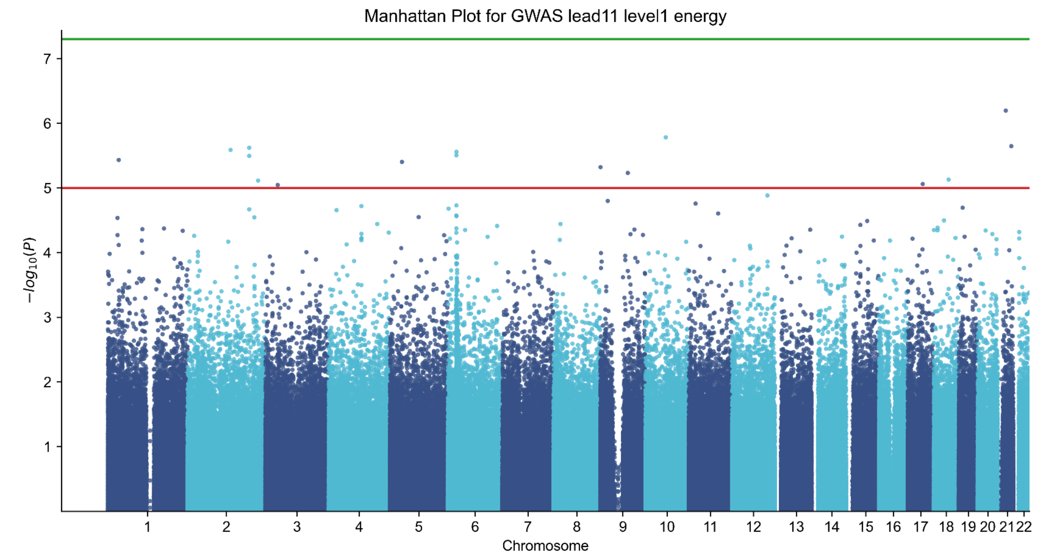

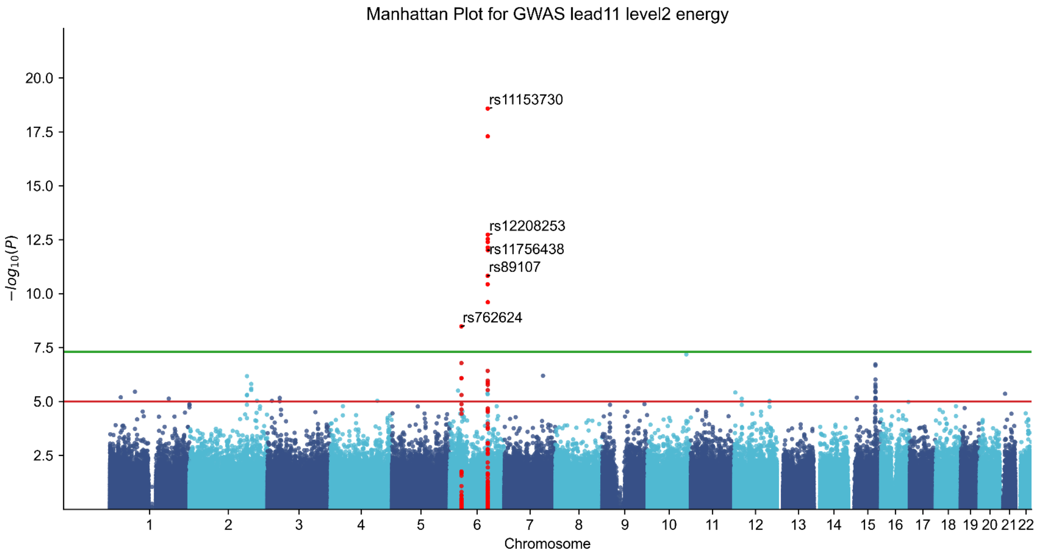

medRxiv preprint doi: https://doi.org/10.64898/2026.05.20.26353725; this version posted June 7, 2026. The copyright holder for this preprint
(which was not certified by peer review) is the author/funder, who has granted medRxiv a license to display the preprint in perpetuity.
It is made available under a CC-BY 4.0 International license .

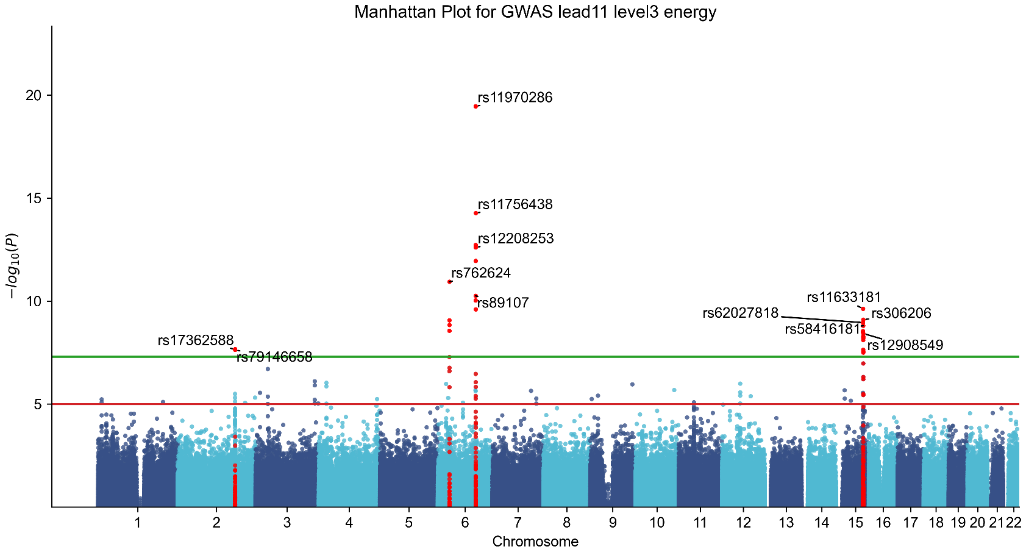

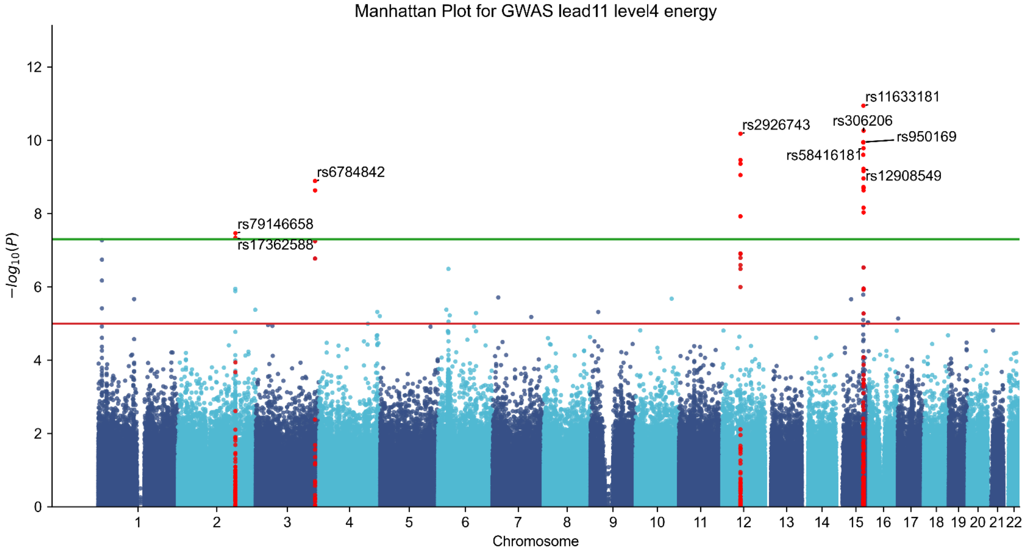

medRxiv preprint doi: https://doi.org/10.64898/2026.05.20.26353725; this version posted June 7, 2026. The copyright holder for this preprint (which was not certified by peer review) is the author/funder, who has granted medRxiv a license to display the preprint in perpetuity.
It is made available under a CC-BY 4.0 International license .

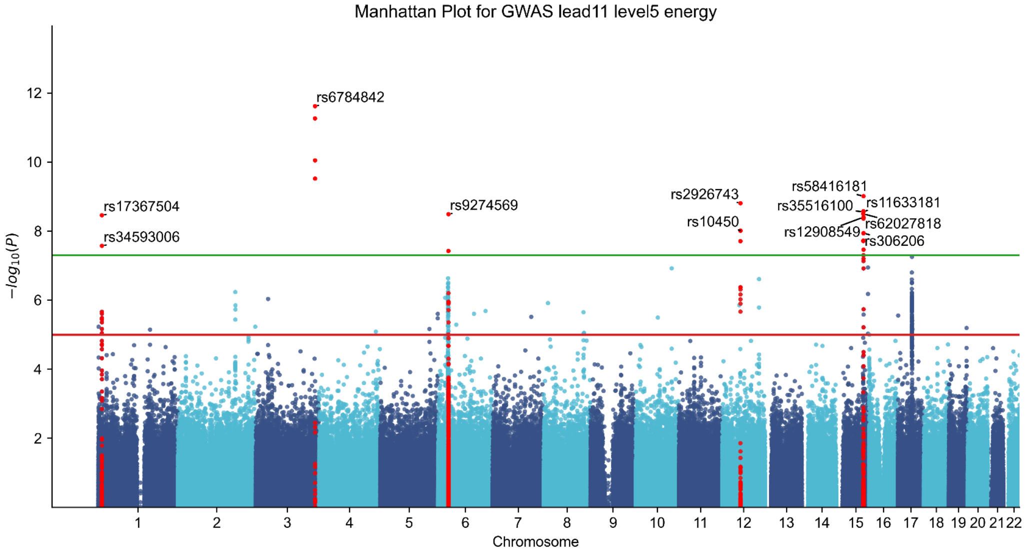

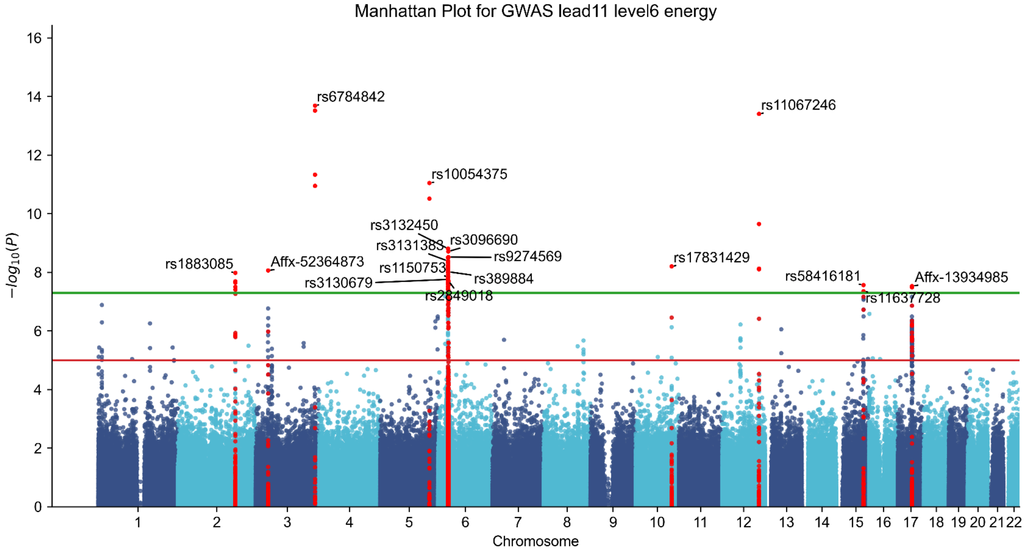

medRxiv preprint doi: https://doi.org/10.64898/2026.05.20.26353725; this version posted June 7, 2026. The copyright holder for this preprint (which was not certified by peer review) is the author/funder, who has granted medRxiv a license to display the preprint in perpetuity.
It is made available under a CC-BY 4.0 International license .

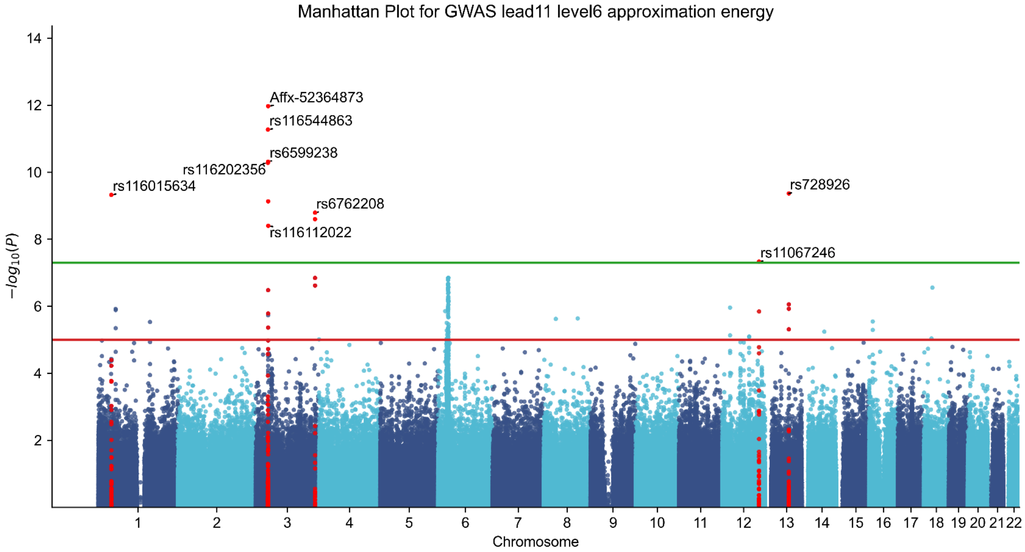

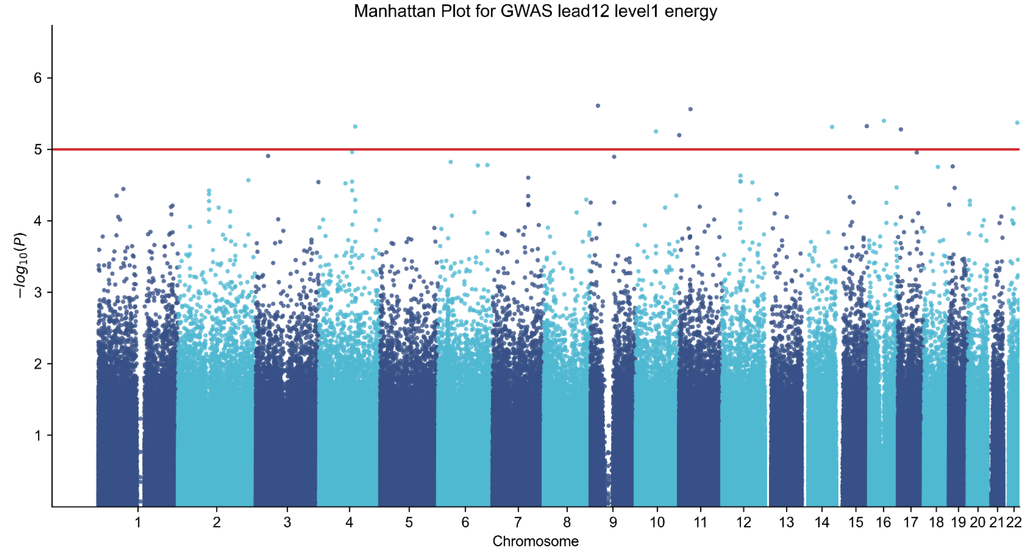

medRxiv preprint doi: https://doi.org/10.64898/2026.05.20.26353725; this version posted June 7, 2026. The copyright holder for this preprint
(which was not certified by peer review) is the author/funder, who has granted medRxiv a license to display the preprint in perpetuity.
It is made available under a CC-BY 4.0 International license .

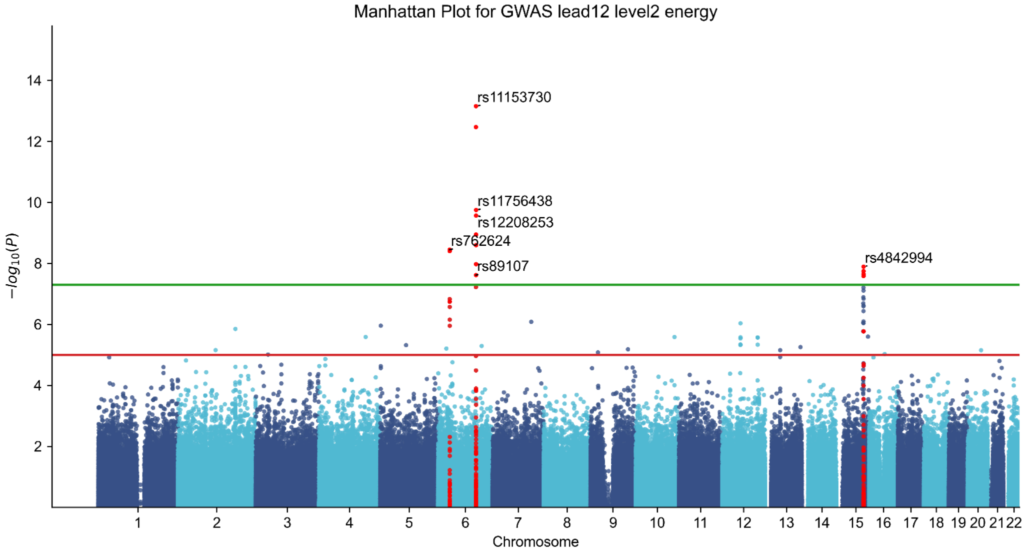

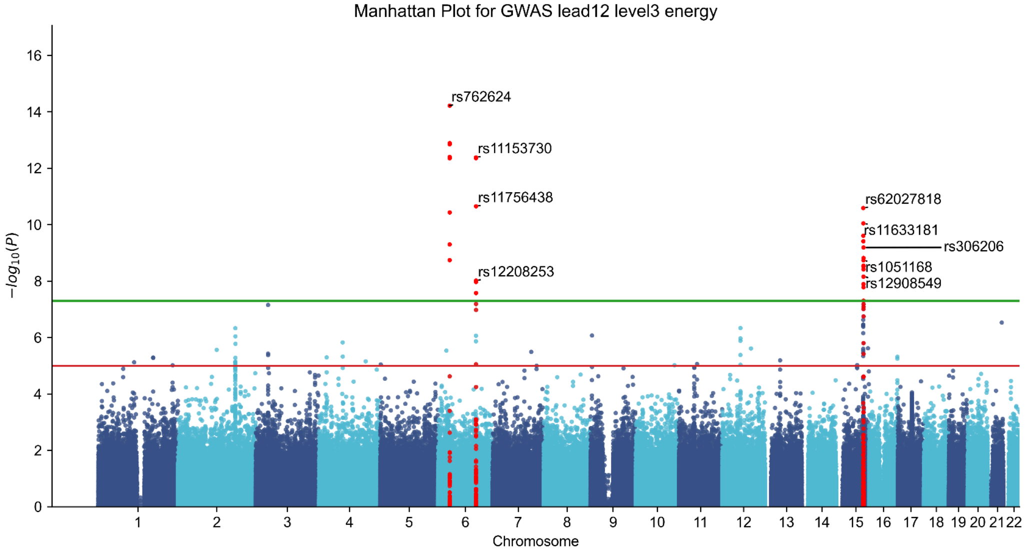

medRxiv preprint doi: https://doi.org/10.64898/2026.05.20.26353725; this version posted June 7, 2026. The copyright holder for this preprint (which was not certified by peer review) is the author/funder, who has granted medRxiv a license to display the preprint in perpetuity.
It is made available under a CC-BY 4.0 International license .

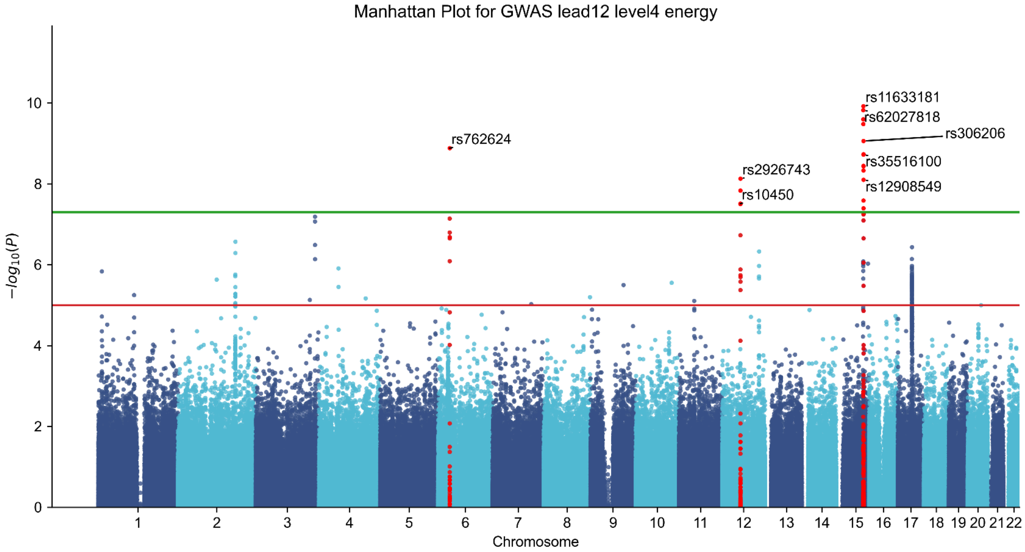

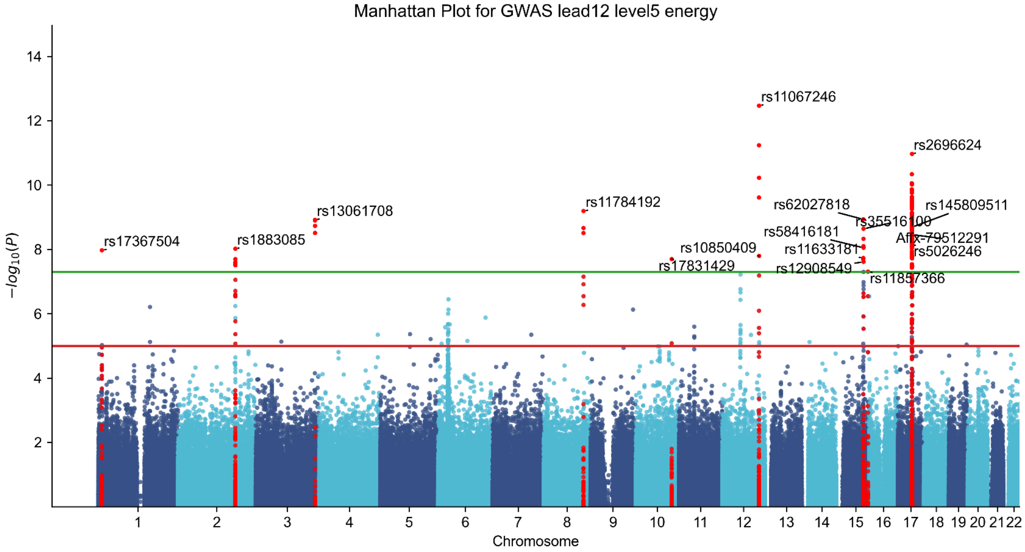

medRxiv preprint doi: https://doi.org/10.64898/2026.05.20.26353725; this version posted June 7, 2026. The copyright holder for this preprint (which was not certified by peer review) is the author/funder, who has granted medRxiv a license to display the preprint in perpetuity.
It is made available under a CC-BY 4.0 International license .

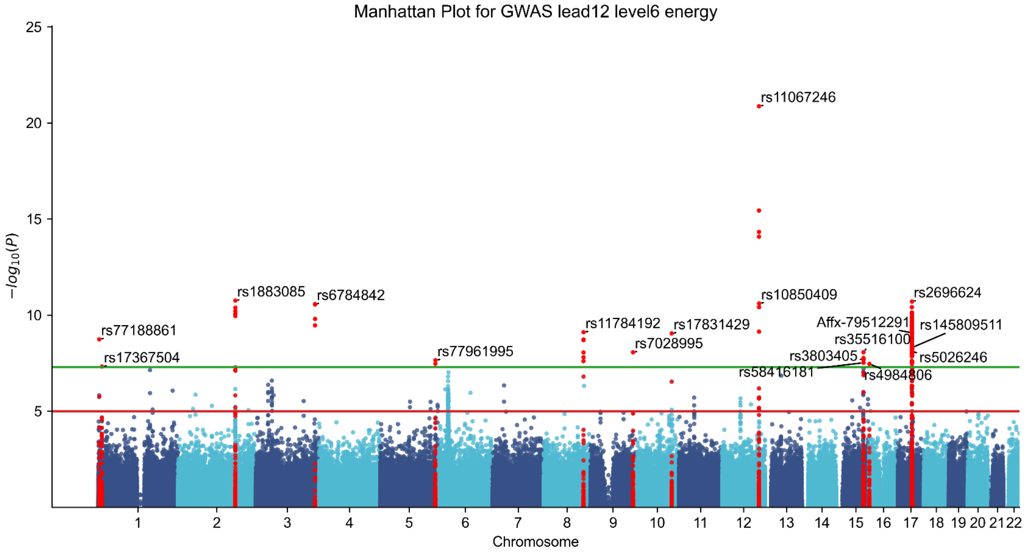

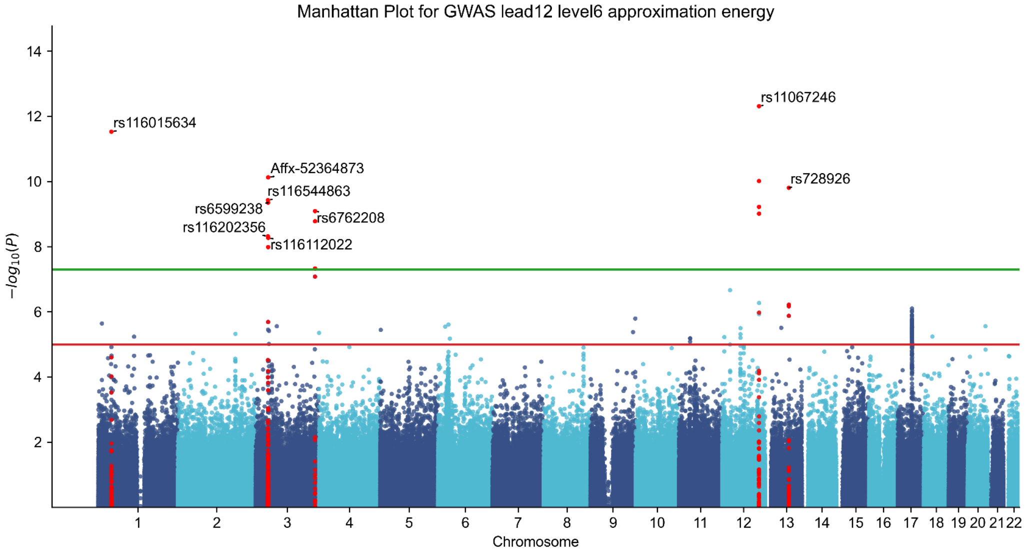

medRxiv preprint doi: https://doi.org/10.64898/2026.05.20.26353725; this version posted June 7, 2026. The copyright holder for this preprint (which was not certified by peer review) is the author/funder, who has granted medRxiv a license to display the preprint in perpetuity.
It is made available under a CC-BY 4.0 International license .

Supplementary Figure 2

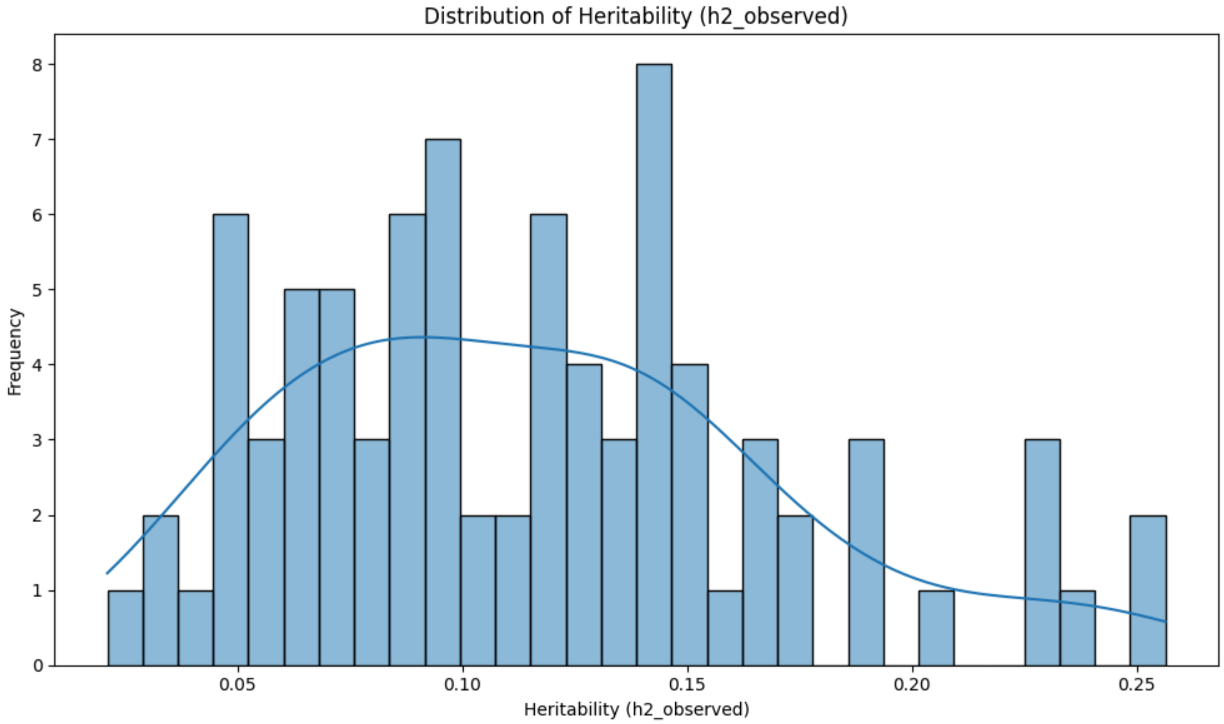

Supplementary Figure 2. Distribution of the heritability of ECG-derived energy features. The strength of the heritability is denoted on the x-axis, and the frequency is denoted on the y-axis.

The distribution of observed heritability (h^2) estimates across the 84 wavelet-derived energy features is depicted in the histogram, revealing a unimodal and right-skewed pattern with values ranging from below 0.03 to a maximum of 0.25. The majority of estimates cluster in the moderate range of 0.05–0.20, with a peak frequency around 0.12–0.15. These h^2 values align with GWAS-based heritability estimates for standard ECG traits, such as QRS duration (~0.16–0.17) and QT interval (~0.17). The right-skewed tail towards higher h^2 (up to 0.25) suggests a subset of features, particularly those in mid-detail levels (D6–D4), captures stronger heritable signals, thereby validating the wavelet decomposition approach as a tool for dissecting nuanced genetic influences on cardiac traits beyond traditional metrics.

medRxiv preprint doi: https://doi.org/10.64898/2026.05.20.26353725; this version posted June 7, 2026. The copyright holder for this preprint (which was not certified by peer review) is the author/funder, who has granted medRxiv a license to display the preprint in perpetuity.
It is made available under a CC-BY 4.0 International license .

Supplementary Figures 3

Genetic correlation estimates across leads and levels.

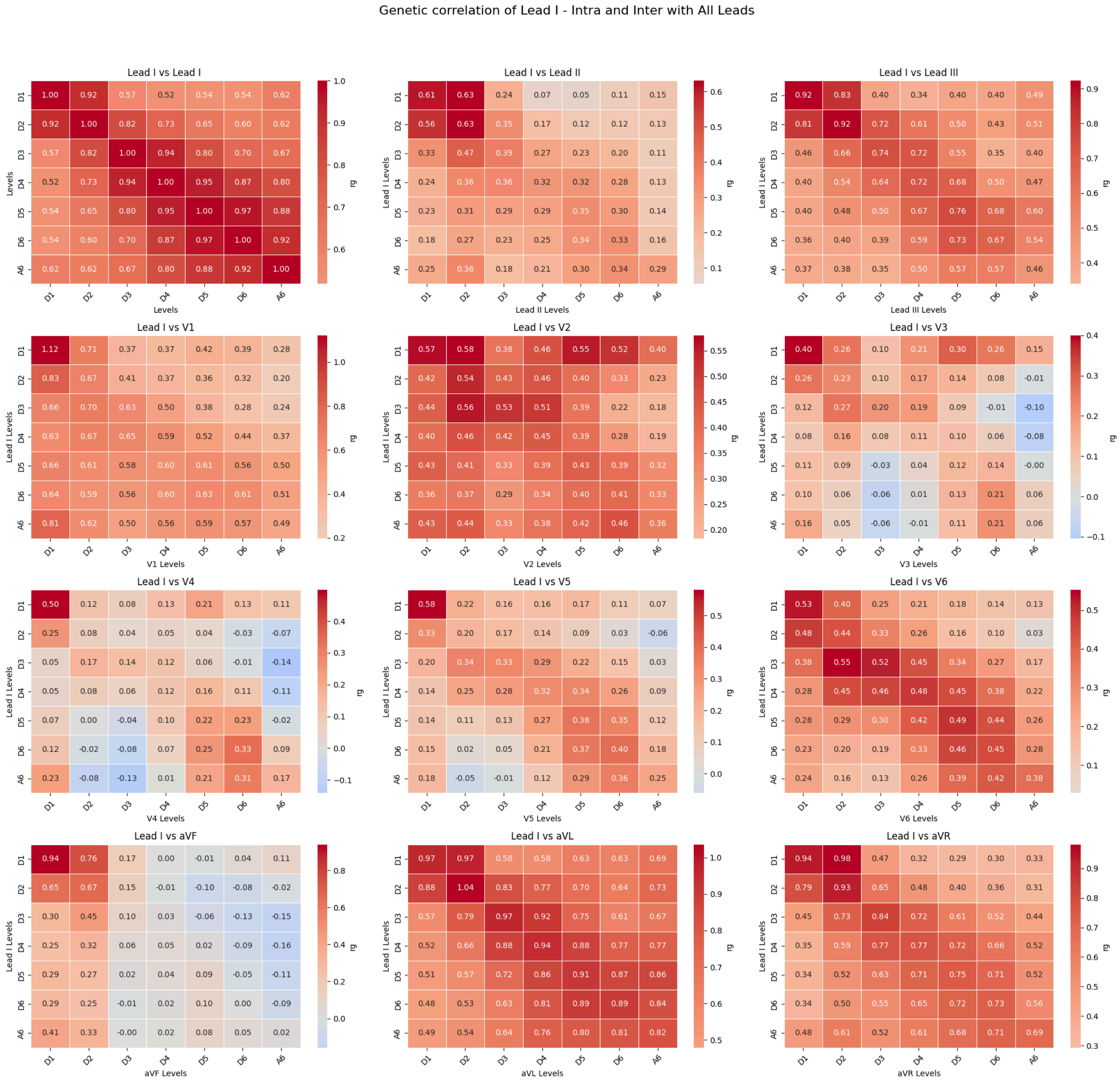

medRxiv preprint doi: https://doi.org/10.64898/2026.05.20.26353725; this version posted June 7, 2026. The copyright holder for this preprint (which was not certified by peer review) is the author/funder, who has granted medRxiv a license to display the preprint in perpetuity.
It is made available under a CC-BY 4.0 International license .

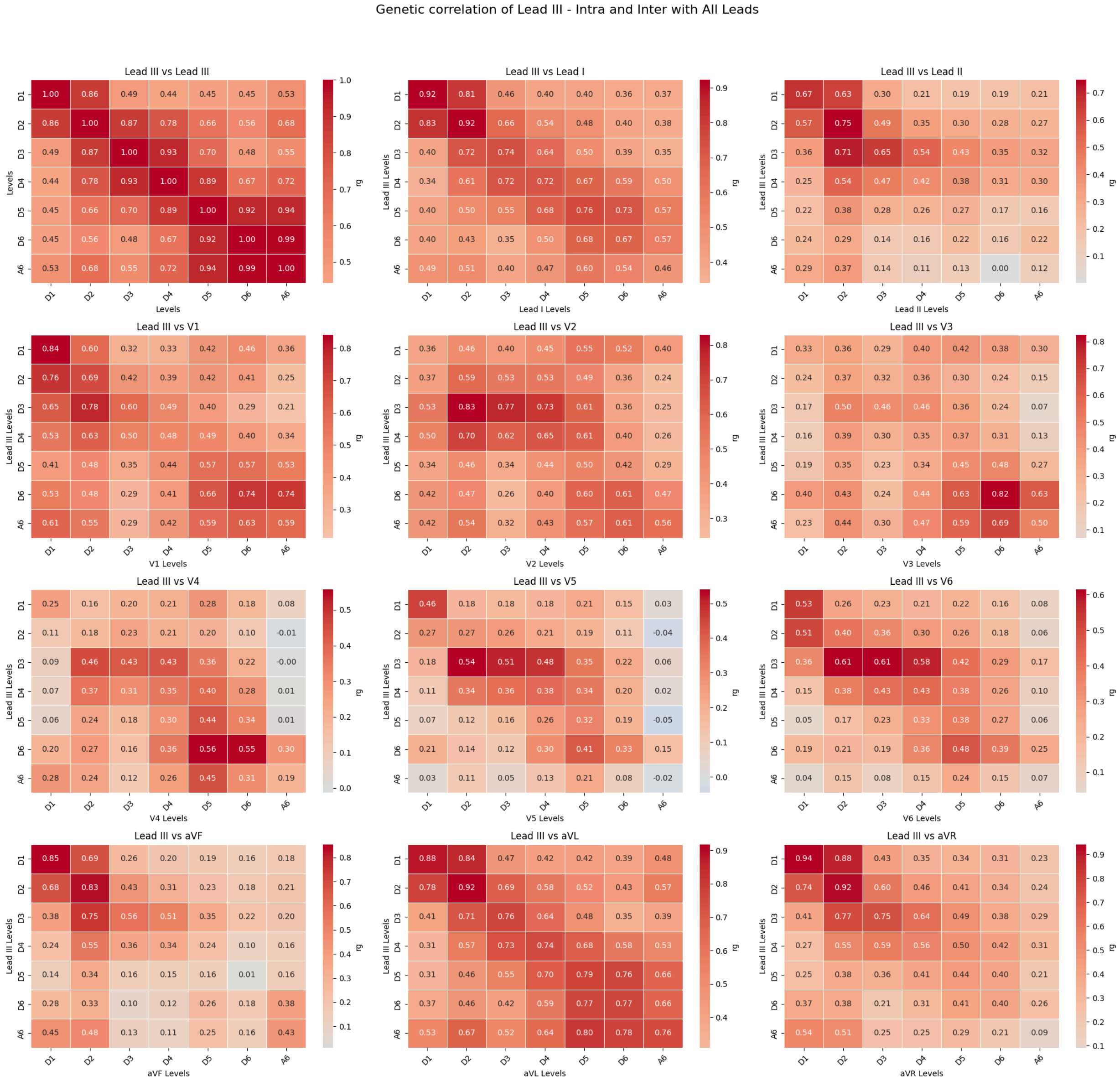

medRxiv preprint doi: https://doi.org/10.64898/2026.05.20.26353725; this version posted June 7, 2026. The copyright holder for this preprint (which was not certified by peer review) is the author/funder, who has granted medRxiv a license to display the preprint in perpetuity.
It is made available under a CC-BY 4.0 International license .

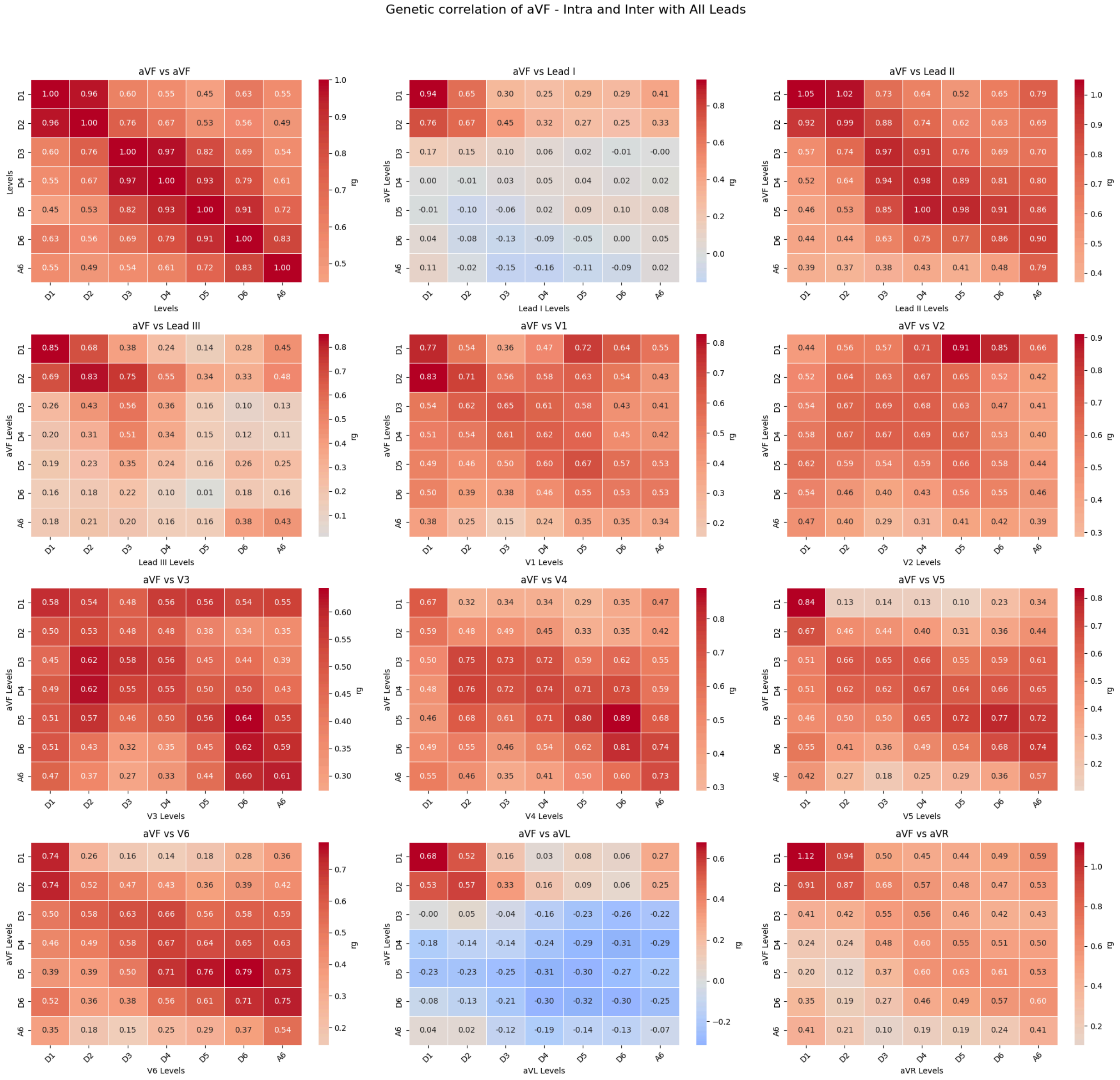

medRxiv preprint doi: https://doi.org/10.64898/2026.05.20.26353725; this version posted June 7, 2026. The copyright holder for this preprint (which was not certified by peer review) is the author/funder, who has granted medRxiv a license to display the preprint in perpetuity.
It is made available under a CC-BY 4.0 International license .

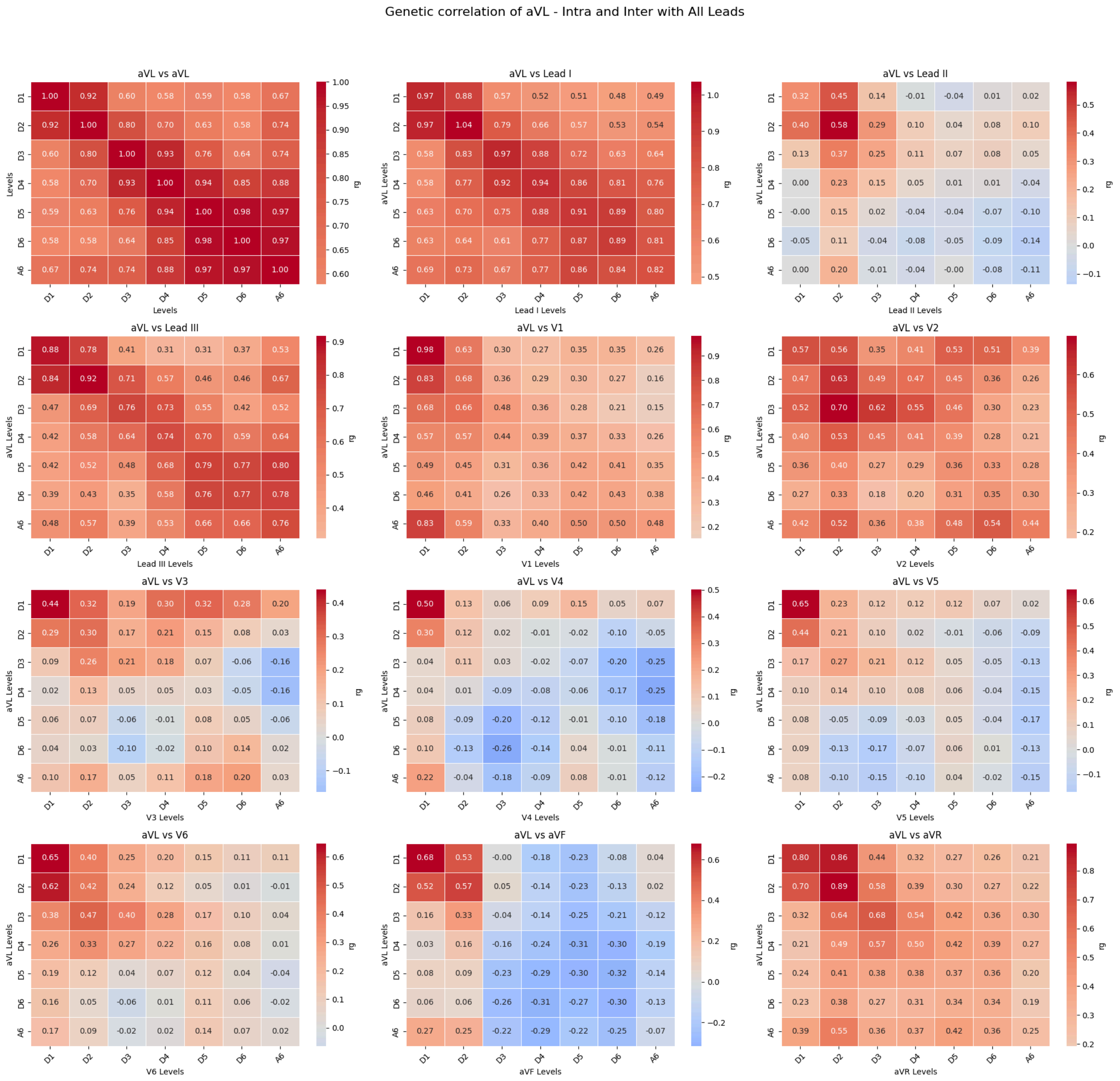

medRxiv preprint doi: https://doi.org/10.64898/2026.05.20.26353725; this version posted June 7, 2026. The copyright holder for this preprint (which was not certified by peer review) is the author/funder, who has granted medRxiv a license to display the preprint in perpetuity.
It is made available under a CC-BY 4.0 International license .

Genetic correlation of aVR - Intra and Inter with All Leads

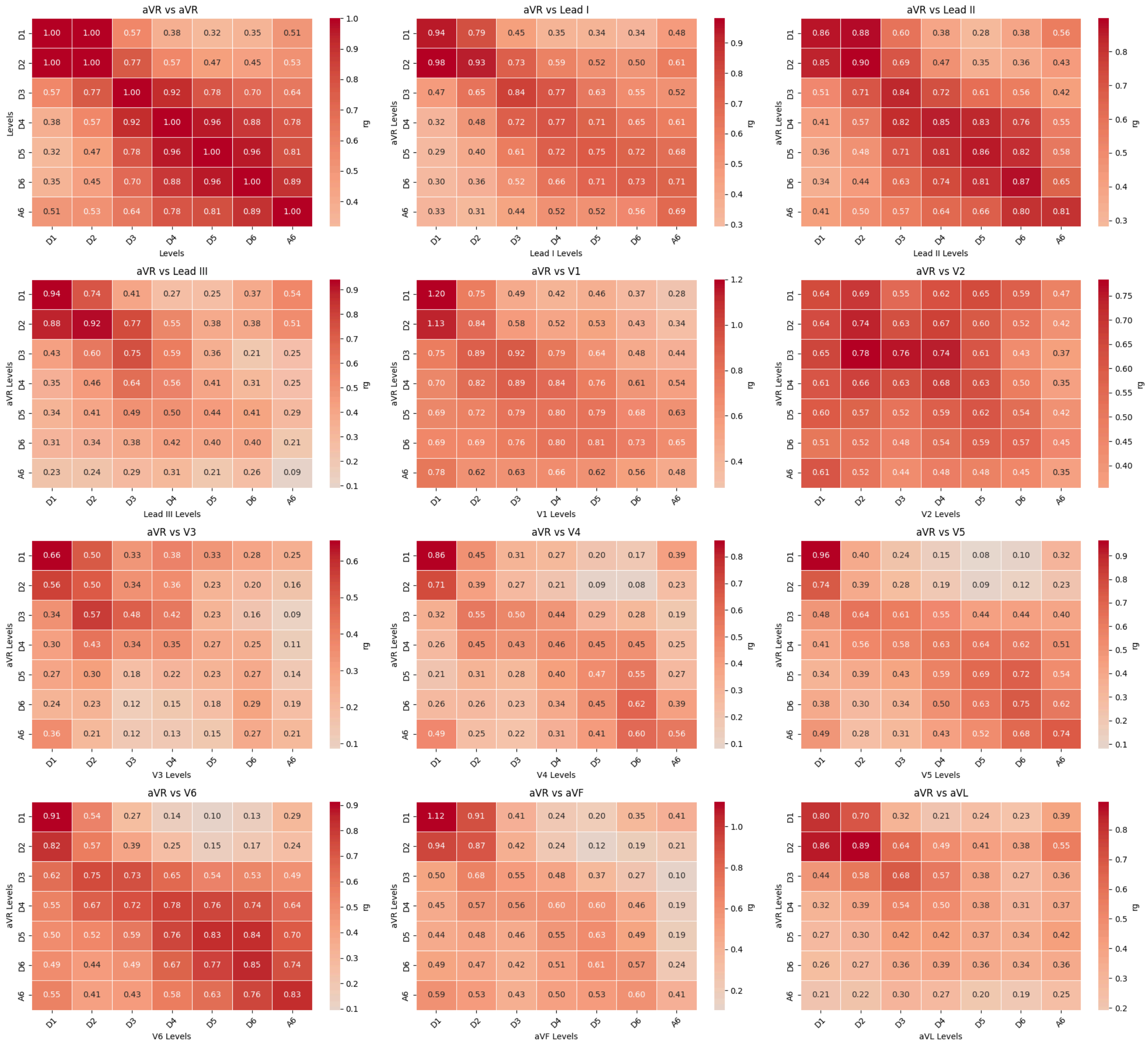

medRxiv preprint doi: https://doi.org/10.64898/2026.05.20.26353725; this version posted June 7, 2026. The copyright holder for this preprint (which was not certified by peer review) is the author/funder, who has granted medRxiv a license to display the preprint in perpetuity.
It is made available under a CC-BY 4.0 International license .

Genetic correlation of V1 - Intra and Inter with All Leads

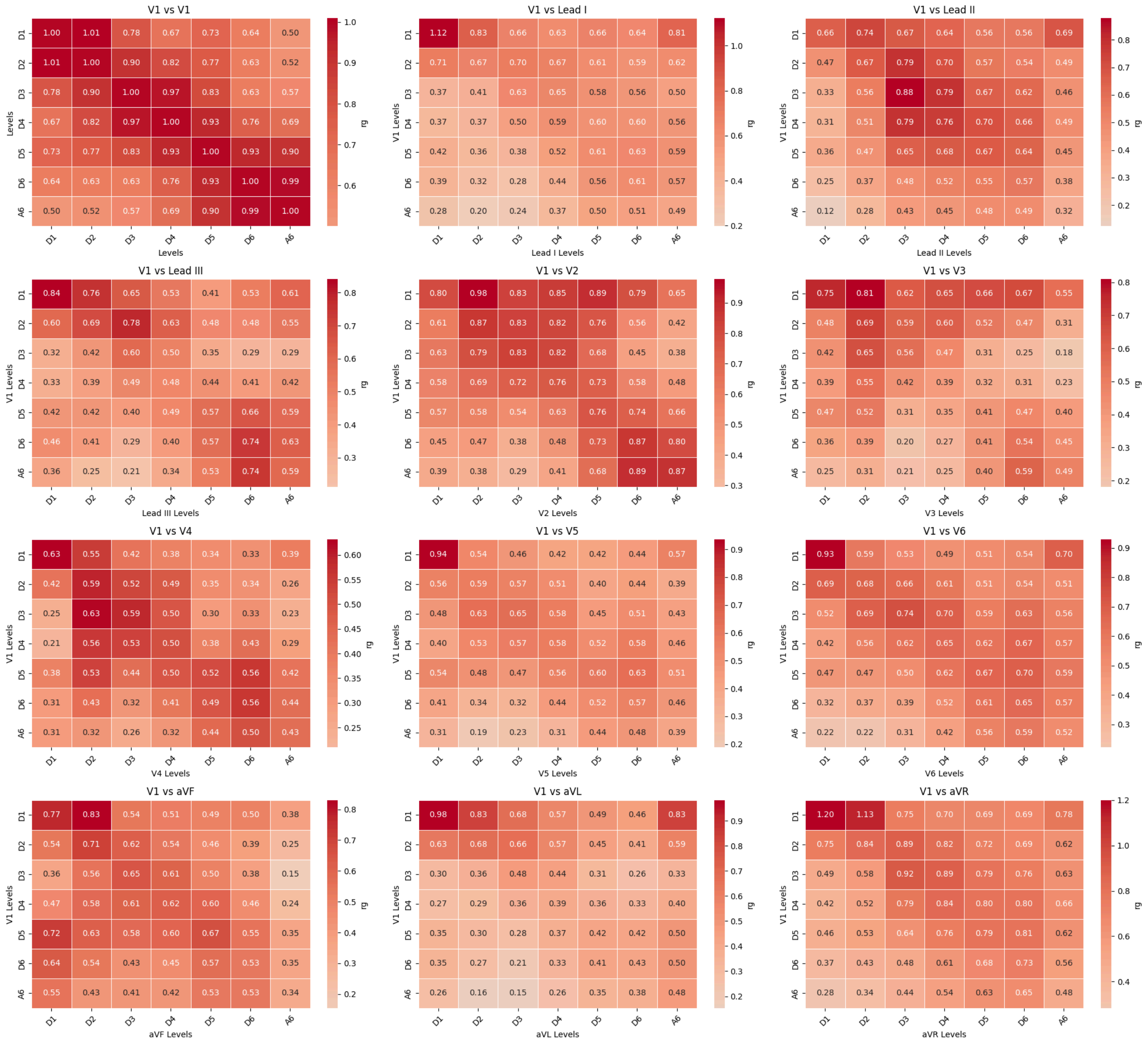

medRxiv preprint doi: https://doi.org/10.64898/2026.05.20.26353725; this version posted June 7, 2026. The copyright holder for this preprint (which was not certified by peer review) is the author/funder, who has granted medRxiv a license to display the preprint in perpetuity.
It is made available under a CC-BY 4.0 International license .

Genetic correlation of V2 - Intra and Inter with All Leads

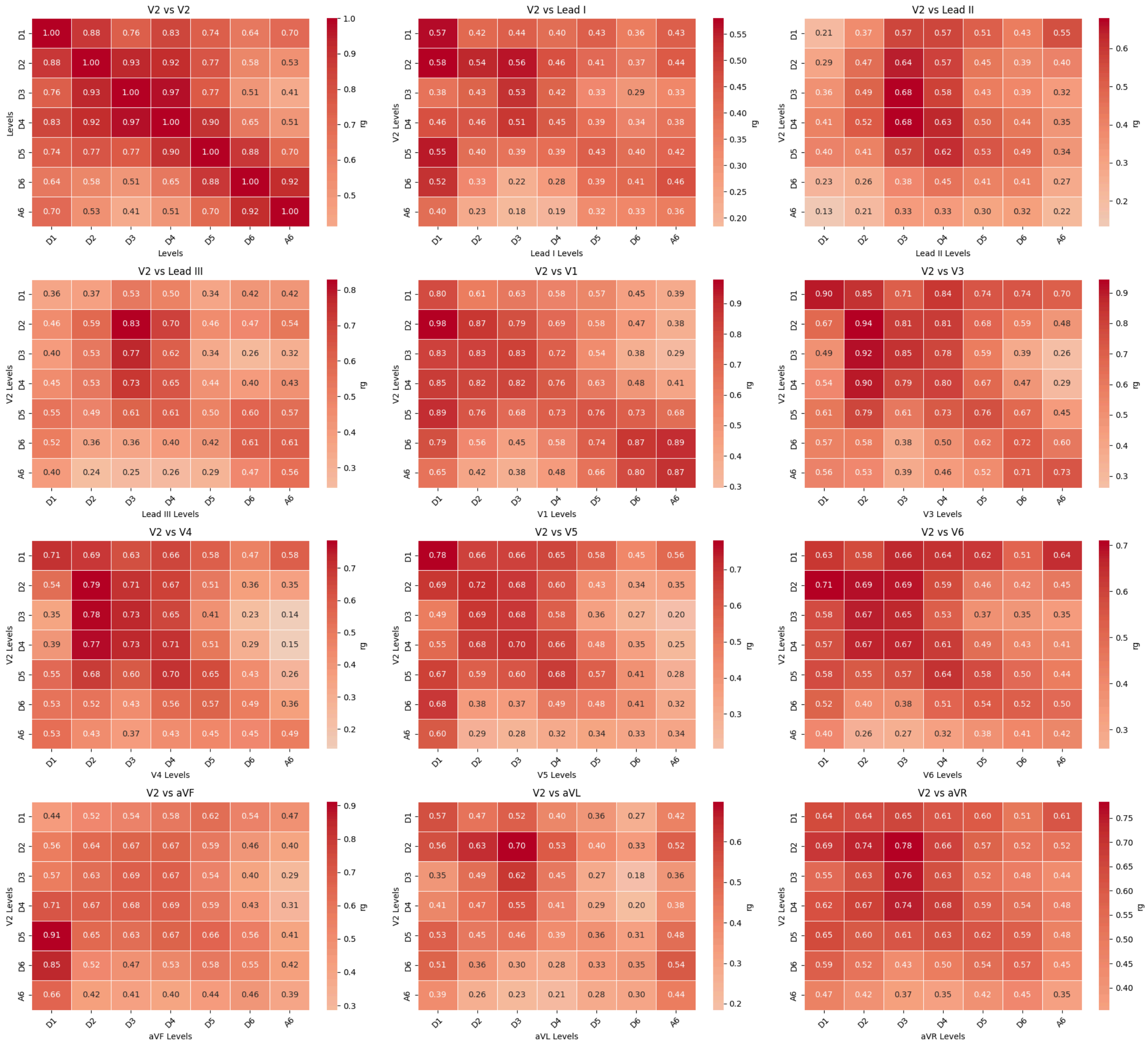

medRxiv preprint doi: https://doi.org/10.64898/2026.05.20.26353725; this version posted June 7, 2026. The copyright holder for this preprint (which was not certified by peer review) is the author/funder, who has granted medRxiv a license to display the preprint in perpetuity.
It is made available under a CC-BY 4.0 International license .

Genetic correlation of V3 - Intra and Inter with All Leads

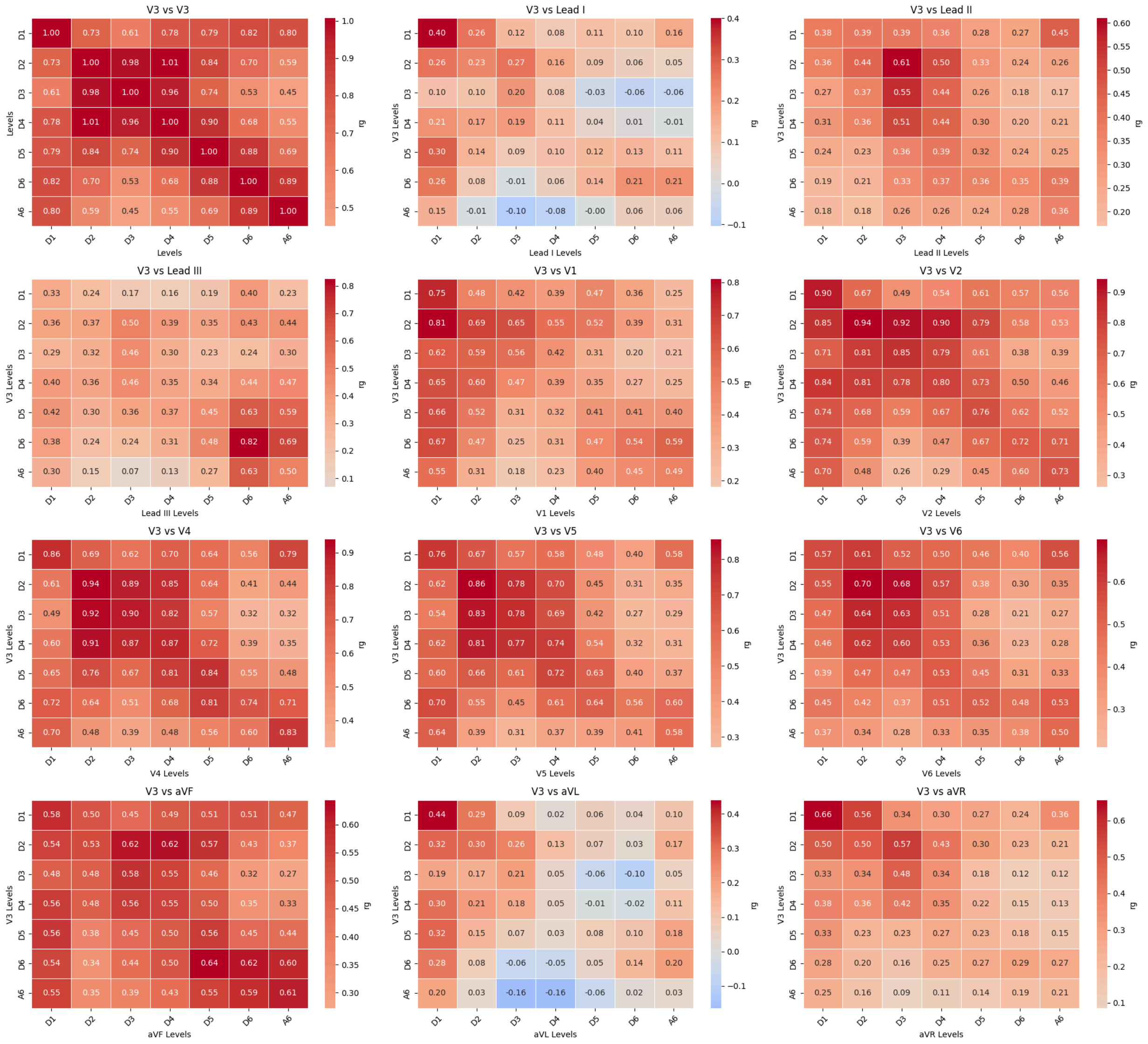

medRxiv preprint doi: https://doi.org/10.64898/2026.05.20.26353725; this version posted June 7, 2026. The copyright holder for this preprint (which was not certified by peer review) is the author/funder, who has granted medRxiv a license to display the preprint in perpetuity.
It is made available under a CC-BY 4.0 International license .

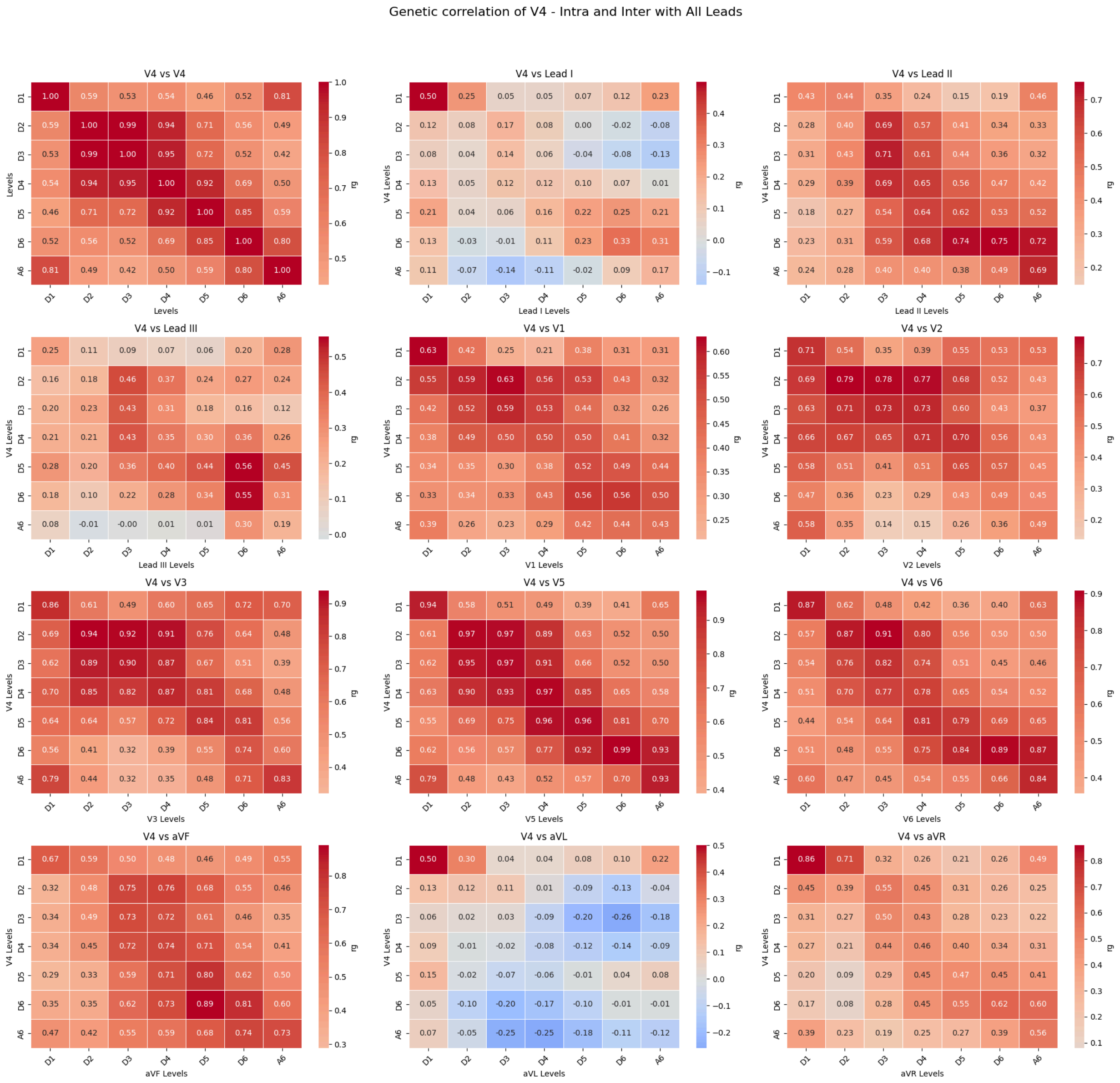

medRxiv preprint doi: https://doi.org/10.64898/2026.05.20.26353725; this version posted June 7, 2026. The copyright holder for this preprint (which was not certified by peer review) is the author/funder, who has granted medRxiv a license to display the preprint in perpetuity.
It is made available under a CC-BY 4.0 International license .

Genetic correlation of V5 - Intra and Inter with All Leads

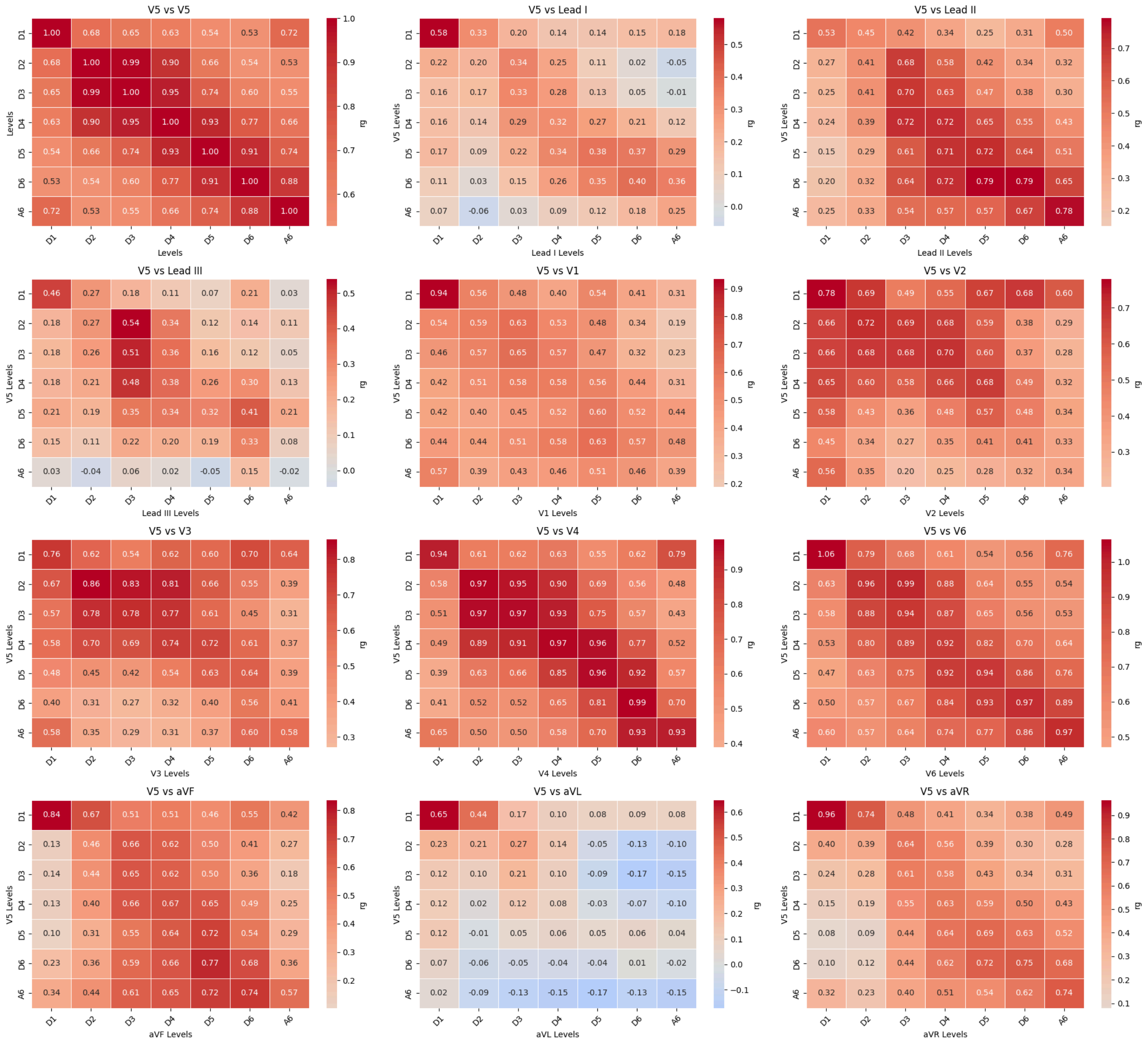

medRxiv preprint doi: https://doi.org/10.64898/2026.05.20.26353725; this version posted June 7, 2026. The copyright holder for this preprint (which was not certified by peer review) is the author/funder, who has granted medRxiv a license to display the preprint in perpetuity.
It is made available under a CC-BY 4.0 International license .

Genetic correlation of V6 - Intra and Inter with All Leads

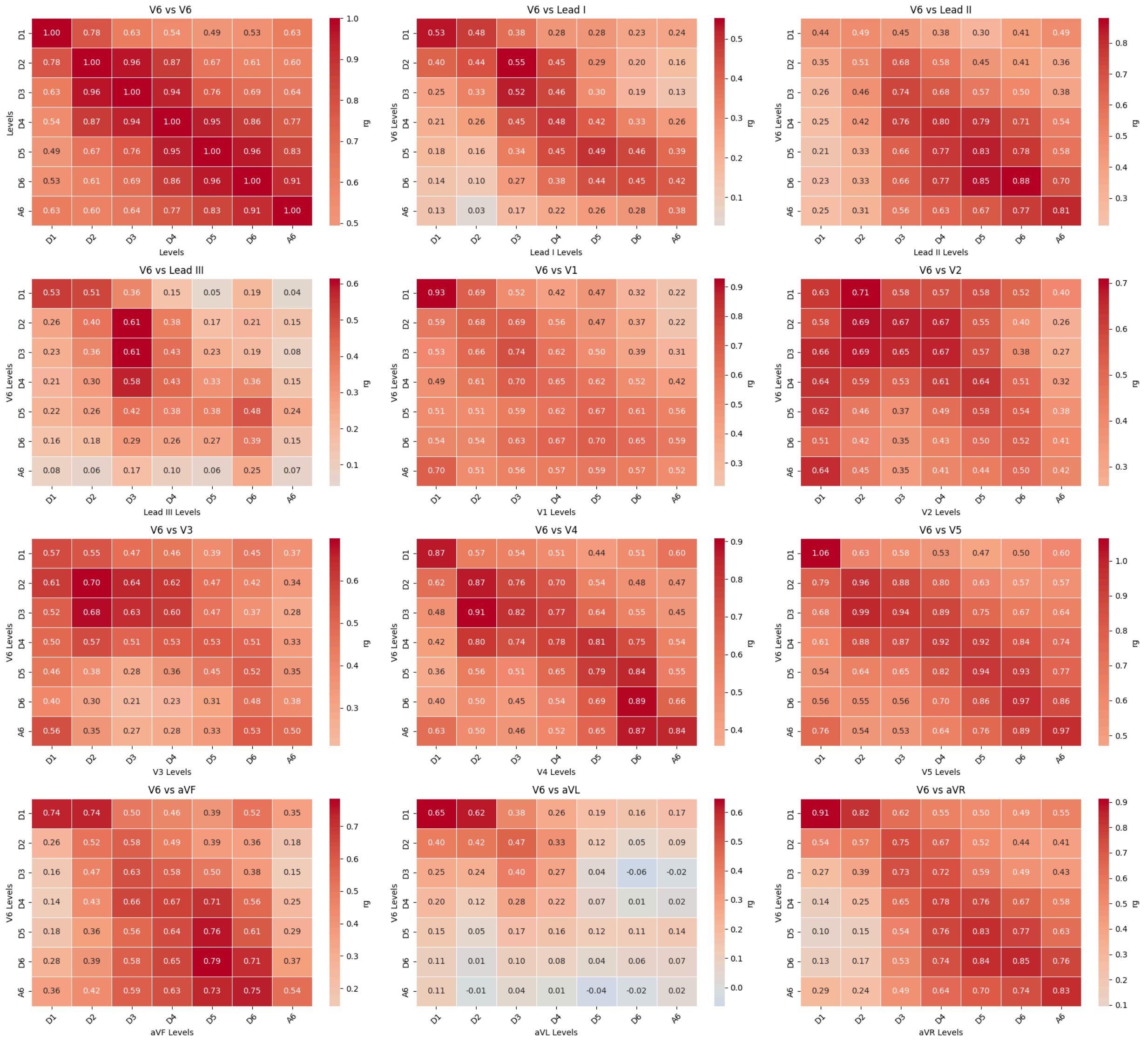

medRxiv preprint doi: https://doi.org/10.64898/2026.05.20.26353725; this version posted June 7, 2026. The copyright holder for this preprint (which was not certified by peer review) is the author/funder, who has granted medRxiv a license to display the preprint in perpetuity.
It is made available under a CC-BY 4.0 International license .

Supplementary Table 1 : Significant genetic correlations between ECG energy features and cardiovascular phenotypes (p < .005 shown).

Cardiovascular phenotypes	Energy features	rg	p
Heart failure and coronary heart disease	Lead I D1	0.34	0.0013
Atherosclerosis (excluding cerebral and coronary sclerosis)	Lead I D1	0.3138	0.0016
Heart failure and coronary heart disease	Lead I D2	0.2558	0.0036
Atherosclerosis (excluding cerebral and coronary sclerosis)	Lead I D2	0.3234	0.0004
Heart failure and coronary heart disease	Lead I D3	0.2065	0.0002
Coronary heart disease wide definition	Lead I D4	0.1406	0.0034
Heart failure and coronary heart disease	Lead I D4	0.2215	9.41E-05
Ischaemic heart disease (wide definition)	Lead I D4	0.1252	0.0031
Ischaemic heart disease (wide definition)	Lead I D5	0.1284	0.0015
Coronary heart disease wide definition	Lead I D5	0.138	0.0032
Coronary atherosclerosis	Lead I D5	0.1213	0.0041
Heart failure and coronary heart disease	Lead I D5	0.2124	0.0001
Coronary heart disease wide definition	Lead I D6	0.1367	0.0041
Heart failure and coronary heart disease	Lead I D6	0.2185	0.0002
Heart failure and coronary heart disease	Lead I A6	0.2391	0.0006
Coronary atherosclerosis	Lead II D1	0.2512	0.004
Coronary atherosclerosis	aVR D1	0.2952	0.0031
Atherosclerosis (excluding cerebral and coronary sclerosis)	aVR D1	0.3483	0.0032
Atherosclerosis (excluding cerebral and coronary sclerosis)	aVR D2	0.3682	0.0008
Heart failure and coronary heart disease	aVR D2	0.3238	0.0044
Coronary atherosclerosis	aVR D2	0.2469	0.003
Coronary atherosclerosis	aVR D4	0.1302	0.0041
Heart failure and coronary heart disease	aVR D4	0.1845	0.0033
Heart failure and coronary heart disease	aVR D5	0.1819	0.0016

medRxiv preprint doi: https://doi.org/10.64898/2026.05.20.26353725; this version posted June 7, 2026. The copyright holder for this preprint (which was not certified by peer review) is the author/funder, who has granted medRxiv a license to display the preprint in perpetuity.
It is made available under a CC-BY 4.0 International license .

Coronary atherosclerosis	aVR D5	0.1463	0.0004
Ischaemic heart disease (wide definition)	aVR D5	0.1463	0.0004
Coronary heart disease wide definition	aVR D5	0.1344	0.0029
Heart failure and coronary heart disease	aVR D6	0.1854	0.0019
Heart failure and coronary heart disease	aVL D1	0.3363	0.001
Atherosclerosis (excluding cerebral and coronary sclerosis)	aVL D1	0.2671	0.0048
Atherosclerosis (excluding cerebral and coronary sclerosis)	aVL D2	0.3062	0.0012
Heart failure and coronary heart disease	aVL D3	0.2051	0.0028
Heart failure and coronary heart disease	aVL D4	0.2343	0.0019
Heart failure and coronary heart disease	aVL D5	0.24	0.0027
Left bundle branch block	aVL A6	0.4233	0.0026
Heart failure and coronary heart disease	aVL D6	0.2377	0.0039
Left bundle branch block	aVL D6	0.3744	0.0021
Heart failure and coronary heart disease	aVL A6	0.2954	0.0049
Heart failure and coronary heart disease	V1 D1	0.5591	0.0039
Coronary atherosclerosis	V1 D1	0.3894	0.0027
Ischaemic heart disease (wide definition)	V1 D1	0.4187	0.003
Ischaemic heart disease (wide definition)	V1 D2	0.2082	0.0011
Coronary heart disease wide definition	V1 D2	0.2245	0.0004
Coronary atherosclerosis	V1 D2	0.2292	0.0003
Heart failure and coronary heart disease	V1 D2	0.3353	0.0004
Heart failure and coronary heart disease	V1 D3	0.225	0.0013
Coronary atherosclerosis	V1 D4	0.1479	0.0033
Heart failure and coronary heart disease	V1 D4	0.2735	5.04E-05
Coronary heart disease wide definition	V1 D4	0.1617	0.0026
Coronary atherosclerosis	V1 D5	0.2178	0.0001
Heart failure and coronary heart disease	V1 D5	0.3322	3.79E-05
Ischaemic heart disease (wide definition)	V1 D5	0.2102	0.0002
Coronary heart disease wide definition	V1 D5	0.2237	0.0002
Coronary heart disease wide definition	V1 D6	0.2253	0.0003

medRxiv preprint doi: https://doi.org/10.64898/2026.05.20.26353725; this version posted June 7, 2026. The copyright holder for this preprint (which was not certified by peer review) is the author/funder, who has granted medRxiv a license to display the preprint in perpetuity.
It is made available under a CC-BY 4.0 International license .

Coronary heart disease wide definition	V1 A6	0.3073	4.41E-05
Heart failure and coronary heart disease	V1 A6	0.376	0.0001
Heart failure and coronary heart disease	V1 D6	0.3164	0.0002
Ischaemic heart disease (wide definition)	V1 A6	0.2731	0.0001
Ischaemic heart disease (wide definition)	V1 D6	0.1969	0.0007
Coronary atherosclerosis	V1 A6	0.2967	3.97E-05
Coronary atherosclerosis	V1 D6	0.2097	0.0004
Heart failure and coronary heart disease	V2 D2	0.2784	0.0013
Coronary heart disease wide definition	V2 D2	0.2311	0.0006
Heart failure and coronary heart disease	V2 D3	0.2253	0.0037
Coronary heart disease wide definition	V2 D3	0.2061	0.0012
Ischaemic heart disease (wide definition)	V2 D4	0.1922	0.004
Heart failure and coronary heart disease	V2 D4	0.2479	0.0049
Coronary heart disease wide definition	V2 D4	0.2265	0.001
Ischaemic heart disease (wide definition)	V2 D5	0.2541	0.0012
Coronary heart disease wide definition	V2 D5	0.2559	0.0019
Coronary atherosclerosis	V2 D5	0.2311	0.0031
Ischaemic heart disease (wide definition)	V2 A6	0.2676	0.0012
Heart failure and coronary heart disease	V2 A6	0.2867	0.0045
Coronary heart disease wide definition	V2 D6	0.276	0.0021
Coronary atherosclerosis	V2 D6	0.2795	0.0015
Coronary heart disease wide definition	V2 A6	0.264	0.0017
Coronary atherosclerosis	V2 A6	0.259	0.0015
Ischaemic heart disease (wide definition)	V2 D6	0.2883	0.001
Valvular heart disease	V5 D6	0.2248	0.0016
Coronary heart disease wide definition	V6 D1	0.3458	0.0017
Ischaemic heart disease (wide definition)	V6 D1	0.3709	0.0004
Coronary atherosclerosis	V6 D1	0.3778	0.0004
Heart failure and coronary heart disease	V6 D1	0.419	0.0021
Heart failure and coronary heart disease	V6 D2	0.2372	0.0025
Coronary atherosclerosis	V6 D2	0.1971	0.0017
Ischaemic heart disease (wide definition)	V6 D2	0.1964	0.0016

medRxiv preprint doi: https://doi.org/10.64898/2026.05.20.26353725; this version posted June 7, 2026. The copyright holder for this preprint (which was not certified by peer review) is the author/funder, who has granted medRxiv a license to display the preprint in perpetuity.
It is made available under a CC-BY 4.0 International license .

Supplementary Table 2 : List of 110 genes mapped from clumped leading SNPs via the FUMA snps2genes tool

Gene	Gene	Gene	Gene	Gene
ATP5B	RP11-127H5.1	TMEM198	C1orf167	ARHGAP27
PTGES3	ZFPM2	OBSL1	MTHFR	PLEKHM1
NACA	KLHL38	INHA	CLCN6	CRHR1
PRIM1	PLEC	HRH1	NPPA	SPPL2C
HSD17B6	SPATC1	TMEM40	C1orf234	MAPT
LRCH1	OPLAH	CAND2	KDM1A	STH
DLEU1	EXOSC4	DLEC1	LUZP1	KANSL1
KLF12	CYC1	ACAA1	TCEA3	ARL17B
AC004817.1	SHARPIN	MYD88	NFIA	LRRC37A
SIPA1L1	MAF1	OXSR1	NOS1AP	LRRC37A2
SH3GL3	KIAA1875	SCN5A	OBSCN	ARL17A
ADAMTSL3	EGR2	SCN10A	TRIM11	NSF
GOLGA6L4	CTNNA3	SCN11A	TRIM17	MAPRE2
ZSCAN2	VCL	WDR48	HIST3H3	FHOD3
WDR73	AP3M1	SENP2	C2orf91	
NMB	ADK	IGF2BP2	PRKRA	
SEC11A	ANKRD1	PPARGC1A	DFNB59	
ZNF592	VTI1A	NKX2-5	FKBP7	
ALPK3	KCNQ1	DSP	PLEKHA3	
LMF1	ERBB3	CDKN1A	TTN	
AC009041.2	RP11-603J24.9	SLC35F1	CCDC141	
SOX8	PA2G4	CEP85L	ERBB4	
UBE2I	RBMS2	PLN	ASIC4	
BAIAP3	BAZ2A	CENPW	CHPF	

medRxiv preprint doi: https://doi.org/10.64898/2026.05.20.26353725; this version posted June 7, 2026. The copyright holder for this preprint (which was not certified by peer review) is the author/funder, who has granted medRxiv a license to display the preprint in perpetuity.
It is made available under a CC-BY 4.0 International license .

Supplementary Table 3: Cell type enrichment analysis results from Enrichr

Name	P-value	
Cardiomyocyte Heart Mouse	0.0001471	7.32
Cardiomyocyte Heart Human	0.0003838	19
Sertoli Cell Fetal Gonad Human	0.0008394	27.9
Oligodendrocyte Brain Human	0.003193	0.83
GABAergic Neuron Brain Mouse	0.007645	1.51
Enterocyte Small Intestine Mouse	0.008817	5.52
Rheaume Et al. Nat Commun. 2018 Retina Mouse	0.01105	1.38
Interneuron Embryonic Prefrontal Cortex Human	0.01256	5.51
Oligodendrocyte Progenitor Cell Embryonic Prefrontal Cortex Human	0.02285	9.59
Type IB Spiral Ganglion Neuron Brain Mouse	0.02651	30.32

medRxiv preprint doi: https://doi.org/10.64898/2026.05.20.26353725; this version posted June 7, 2026. The copyright holder for this preprint (which was not certified by peer review) is the author/funder, who has granted medRxiv a license to display the preprint in perpetuity.
It is made available under a CC-BY 4.0 International license .

Supplementary Table 4: Sensitivity analysis for reverse causation in D1 genetic correlations

Phenotype	Lead I	Lead II	Lead III	aVR	aVL	aVF	V1	V2	V3	V4	V5	V6
Heart failure & CHD	0.32**	0.21	0.12	0.30*	0.24*	0.21	0.38**	0.26*	0.06	0.14	0.60	0.52
Coronary atherosclerosis	0.27**	0.29**	0.03	0.32**	0.14	0.17	0.31**	0.14	0.07	0.24*	0.68	0.57*
Coronary heart disease	0.24**	0.26**	0.05	0.26**	0.15*	0.21	0.26**	0.12	0.08	0.23*	0.57	0.46*
Ischaemic heart disease	0.24**	0.23*	-0.01	0.29**	0.12	0.14	0.31**	0.17*	0.07	0.21	0.66	0.56*
Atherosclerosis	0.30**	0.14	0.11	0.27*	0.22*	0.07	0.13	0.12	-0.02	0.07	0.36	0.40
Atrial fibrillation	0.13	0.07	-0.02	0.10	0.06	-0.01	0.10	0.02	-0.03	0.01	0.41	0.32
Cardiomyopathy	0.14	-0.02	0.17	0.06	0.13	0.10	0.10	0.14	0.03	0.05	0.36	0.27
Conduction disorder	0.16	-0.26	0.00	-0.06	0.10	-0.36*	0.00	-0.07	-0.02	-0.13	0.08	-0.16
AV block	0.15	-0.13	0.03	0.04	0.09	-0.22	-0.02	-0.07	0.00	-0.06	0.29	0.02
Left bundle branch block	-0.02	-0.31	-0.14	-0.26	0.01	-0.29	-0.15	0.05	-0.17	-0.27	-0.31	-0.29
Paroxysmal tachycardia	0.13	0.18	0.01	0.19	0.00	0.16	0.04	0.07	0.16	0.10	0.53	0.58
Valvular heart disease	0.06	-0.08	-0.09	-0.04	-0.04	-0.11	-0.12	0.07	0.19	0.11	0.28	0.13

* p<0.05 ** p<0.005 *** p<0.0005

Supplementary Table 5

Supplementary Analysis 1: Predictive Modeling With Wavelet-Derived ECG Features

We tested if wavelet energy features capture heart-specific electrical patterns by training LASSO logistic regression models for three UK Biobank outcomes: atrial fibrillation, acute myocardial infarction and myocardial infarction defined by diagnostic codes. We included physician-diagnosed asthma as a negative-control phenotype. Models used all 84 wavelet-derived energy features together with age, sex

medRxiv preprint doi: https://doi.org/10.64898/2026.05.20.26353725; this version posted June 7, 2026. The copyright holder for this preprint (which was not certified by peer review) is the author/funder, who has granted medRxiv a license to display the preprint in perpetuity.
It is made available under a CC-BY 4.0 International license .

and four genetic principal components as covariates. To provide an orthogonal view of feature–phenotype relationships, we also performed univariate association testing on covariate-residualized energies (Energy ~ age + sex +4PCs), enabling direct visualization of lead- and band-specific patterns.

For atrial fibrillation (AUC = 0.75), predictive signal concentrated in mid-to-high frequency detail bands (D5–D1), most prominently in V1 and adjacent precordial leads, consistent with the loss of organized atrial activity and increased fragmentation of atrial electrical dynamics; conversely, low-frequency components in Lead II and aVR carried negative weights, aligning with preserved, structured atrial activity under sinus rhythm.

For myocardial infarction (AUC = 0.78), selected coefficients were enriched in mid-frequency bands (D6–D4) across anterior–lateral precordial leads (V3–V6), implicating altered QRS morphology and early repolarization dynamics in the predictive signal, with smaller contributions from intermediate bands (D3–D2) suggestive of localized changes in waveform complexity. Notably, multivariable selections were concordant with the univariate lead–band association maps, indicating that sparse models recover coherent, physiologically interpretable patterns rather than diffuse correlates.

In contrast, the asthma negative control showed near-null discrimination (AUC = 0.564) with low-magnitude, spatially unstructured coefficients and weak univariate signals, supporting the specificity of the cardiovascular patterns to electrophysiological remodeling rather than generic confounding.

medRxiv preprint doi: https://doi.org/10.64898/2026.05.20.26353725; this version posted June 7, 2026. The copyright holder for this preprint
(which was not certified by peer review) is the author/funder, who has granted medRxiv a license to display the preprint in perpetuity.
It is made available under a CC-BY 4.0 International license .

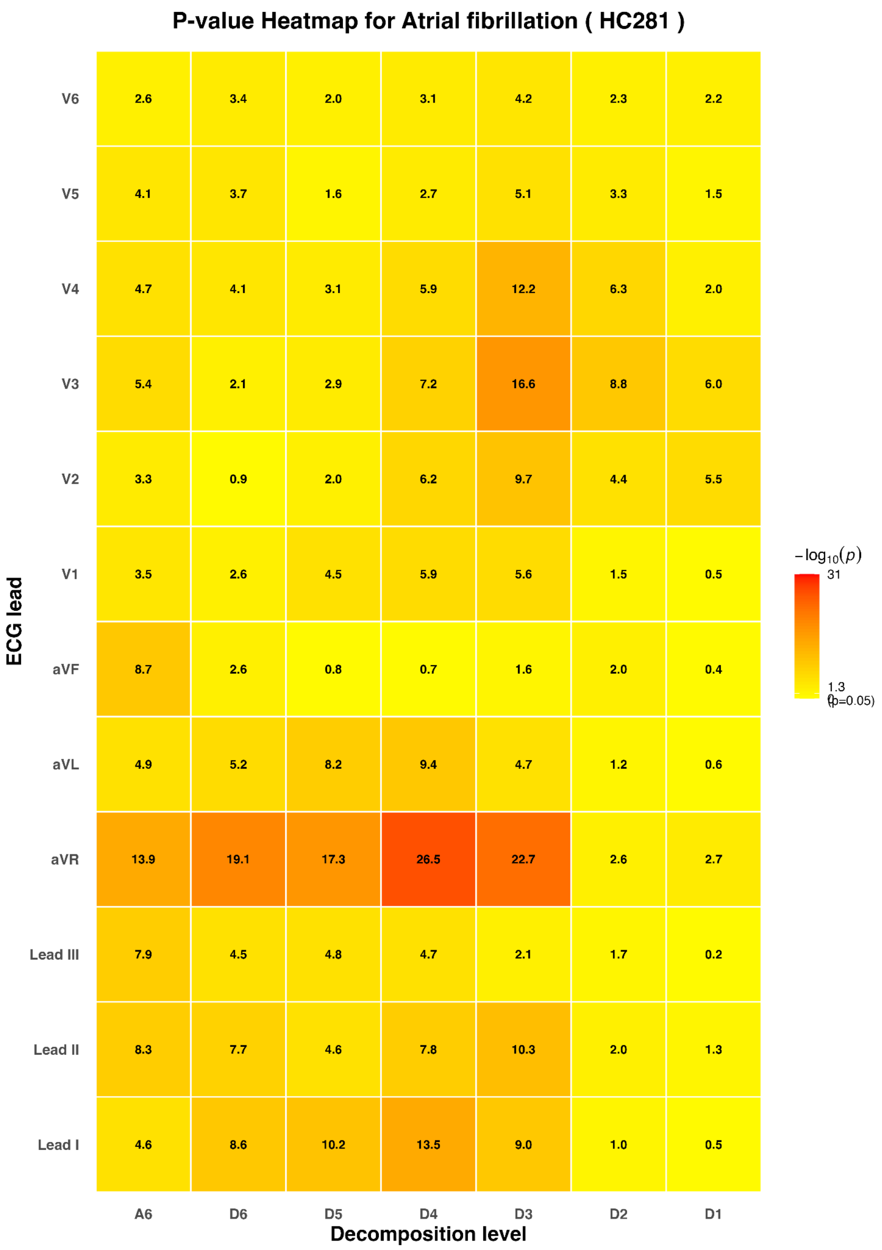

medRxiv preprint doi: https://doi.org/10.64898/2026.05.20.26353725; this version posted June 7, 2026. The copyright holder for this preprint (which was not certified by peer review) is the author/funder, who has granted medRxiv a license to display the preprint in perpetuity.
It is made available under a CC-BY 4.0 International license .

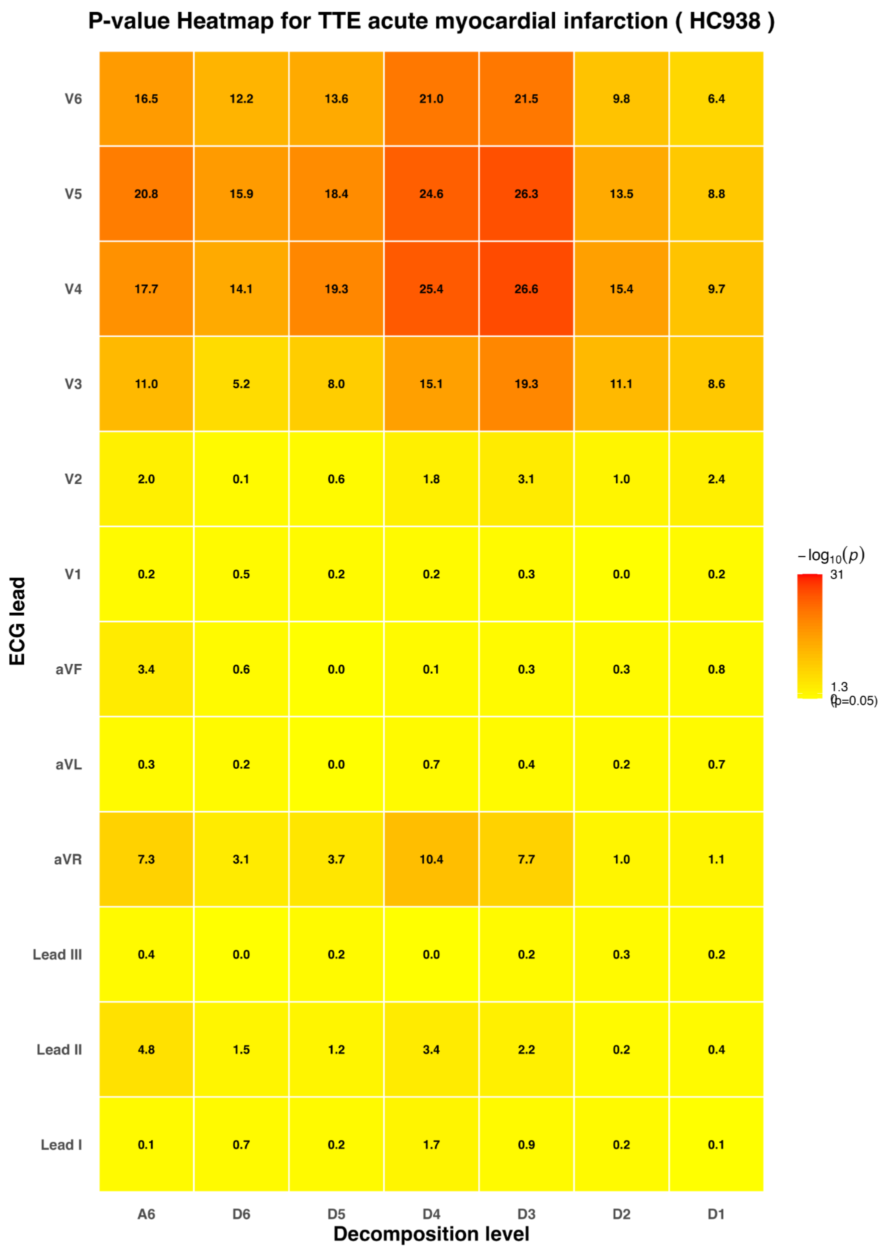

medRxiv preprint doi: https://doi.org/10.64898/2026.05.20.26353725; this version posted June 7, 2026. The copyright holder for this preprint (which was not certified by peer review) is the author/funder, who has granted medRxiv a license to display the preprint in perpetuity.
It is made available under a CC-BY 4.0 International license .

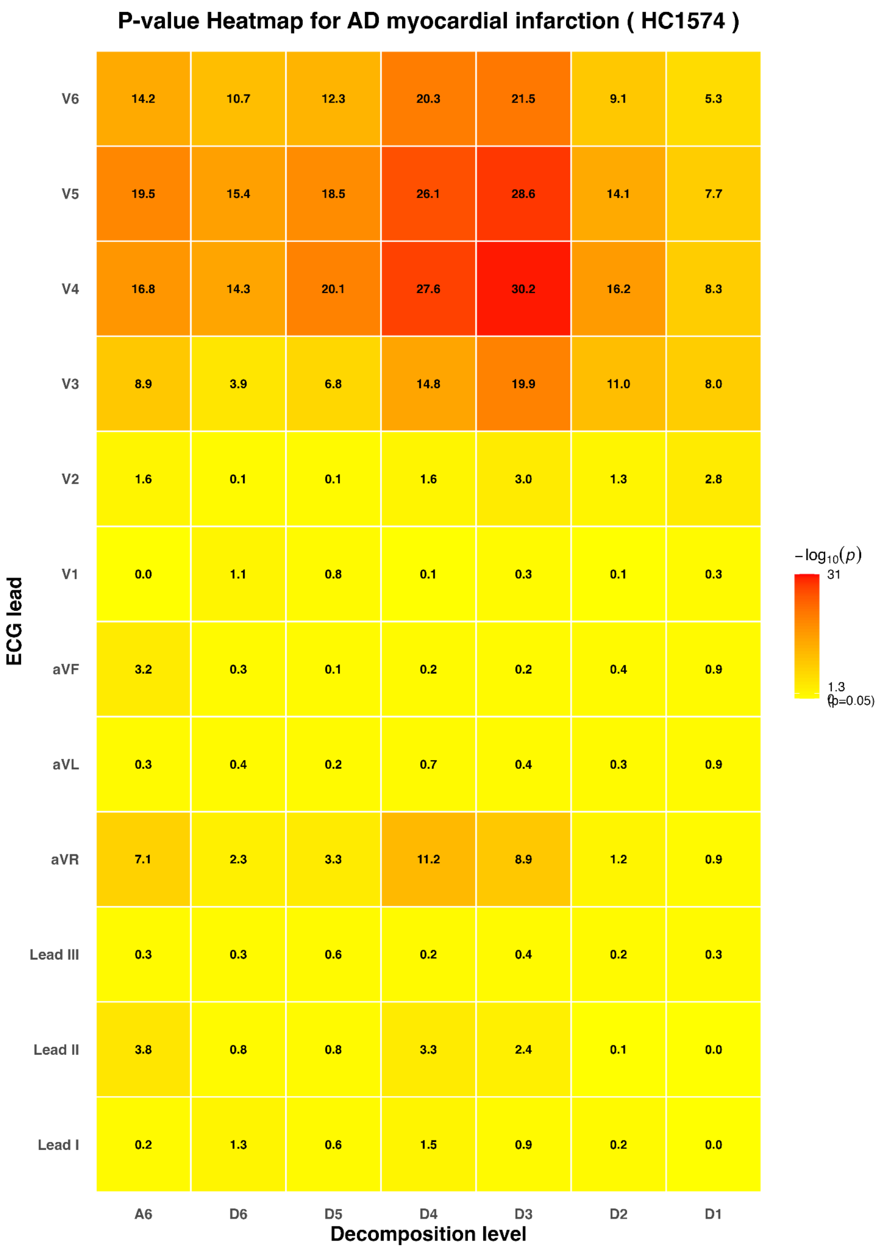

medRxiv preprint doi: https://doi.org/10.64898/2026.05.20.26353725; this version posted June 7, 2026. The copyright holder for this preprint (which was not certified by peer review) is the author/funder, who has granted medRxiv a license to display the preprint in perpetuity.
It is made available under a CC-BY 4.0 International license .

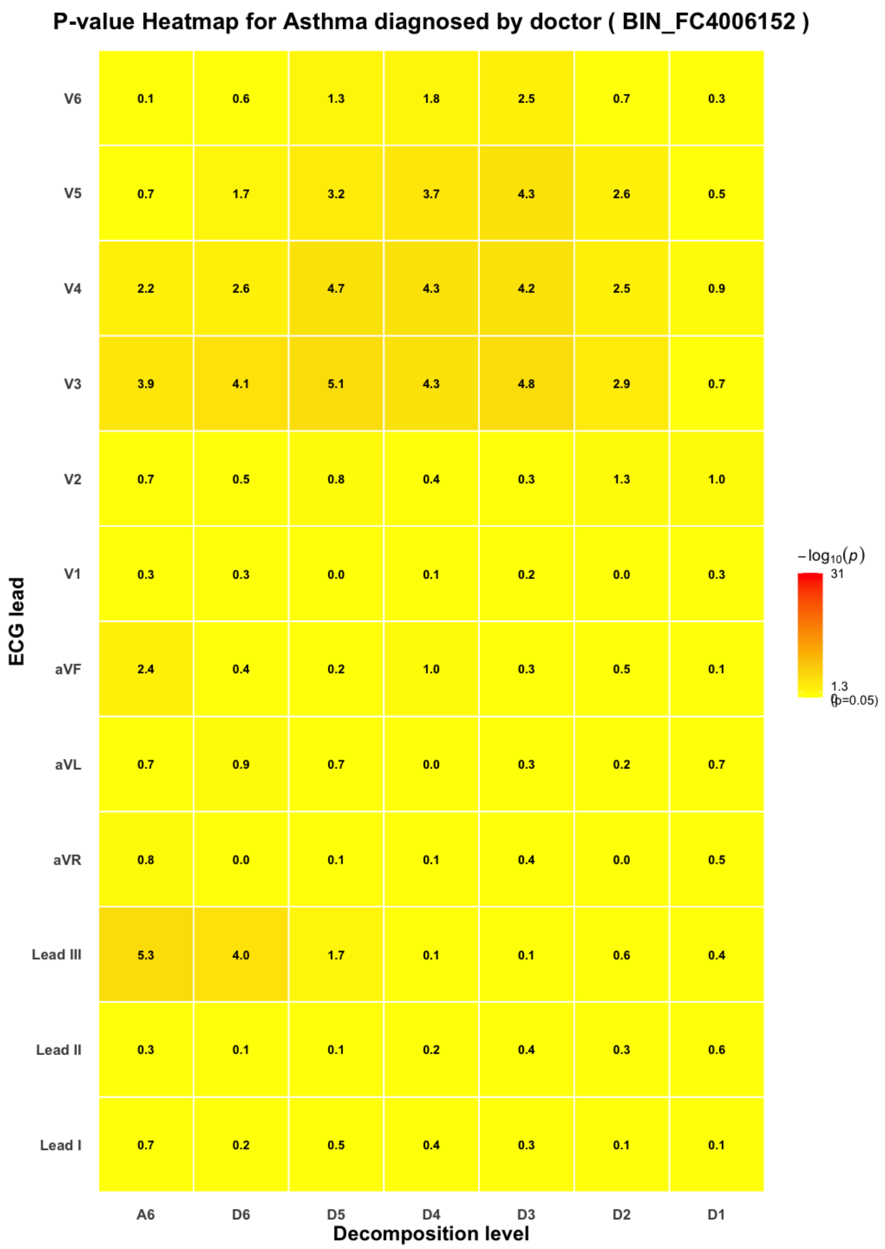

medRxiv preprint doi: https://doi.org/10.64898/2026.05.20.26353725; this version posted June 7, 2026. The copyright holder for this preprint
(which was not certified by peer review) is the author/funder, who has granted medRxiv a license to display the preprint in perpetuity.
It is made available under a CC-BY 4.0 International license .

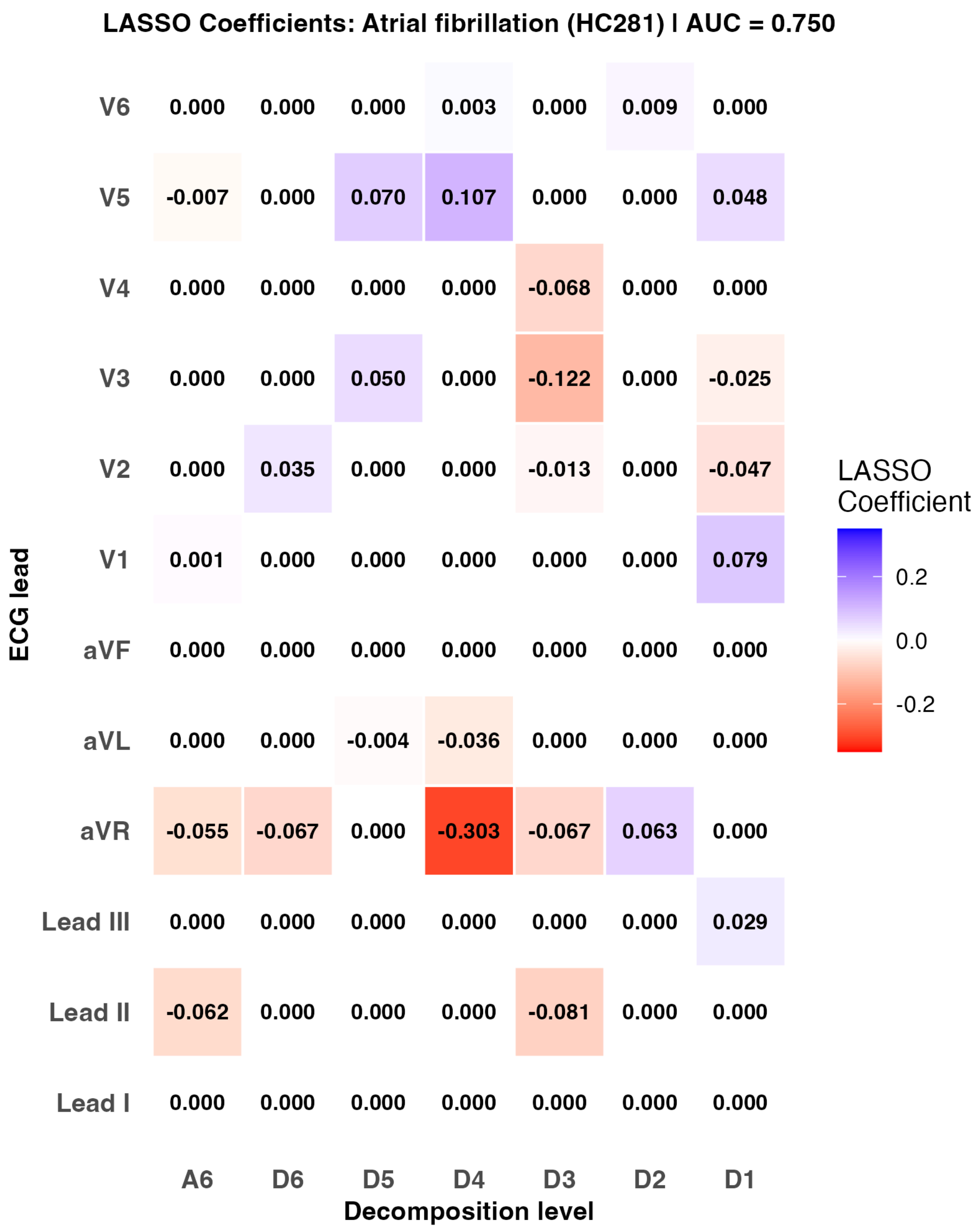

medRxiv preprint doi: https://doi.org/10.64898/2026.05.20.26353725; this version posted June 7, 2026. The copyright holder for this preprint (which was not certified by peer review) is the author/funder, who has granted medRxiv a license to display the preprint in perpetuity.
It is made available under a CC-BY 4.0 International license .

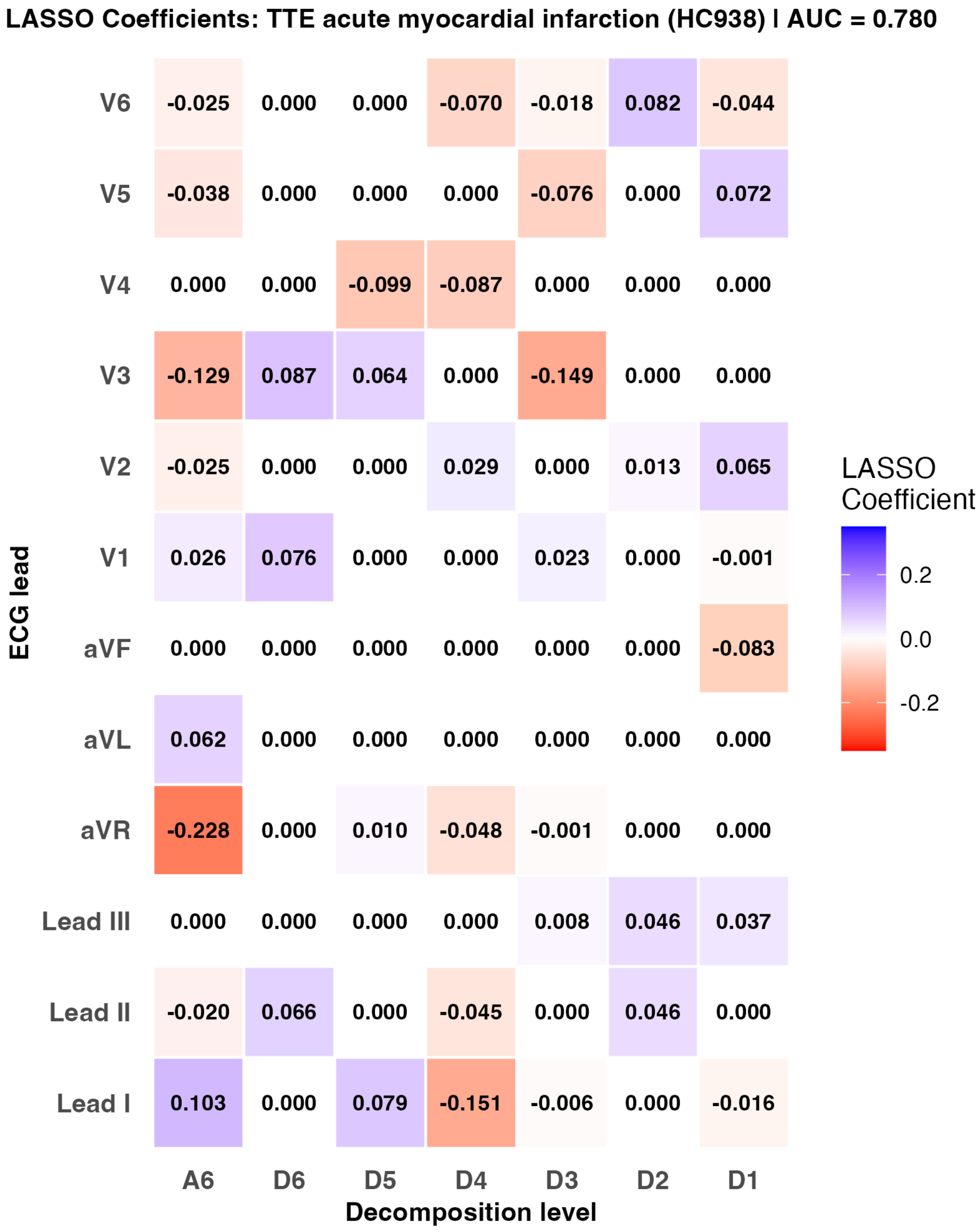

medRxiv preprint doi: https://doi.org/10.64898/2026.05.20.26353725; this version posted June 7, 2026. The copyright holder for this preprint (which was not certified by peer review) is the author/funder, who has granted medRxiv a license to display the preprint in perpetuity.
It is made available under a CC-BY 4.0 International license .

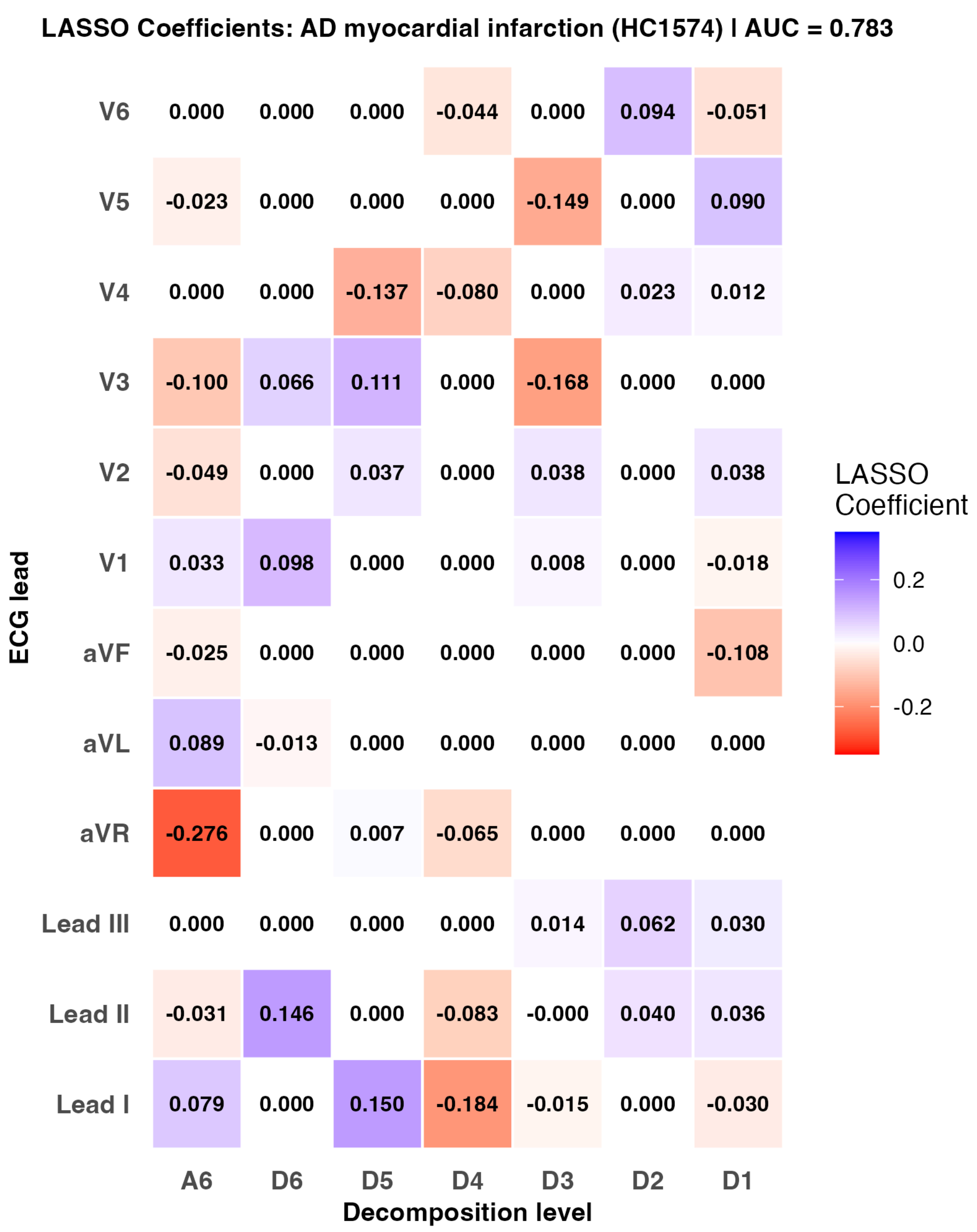

medRxiv preprint doi: https://doi.org/10.64898/2026.05.20.26353725; this version posted June 7, 2026. The copyright holder for this preprint (which was not certified by peer review) is the author/funder, who has granted medRxiv a license to display the preprint in perpetuity.
It is made available under a CC-BY 4.0 International license .

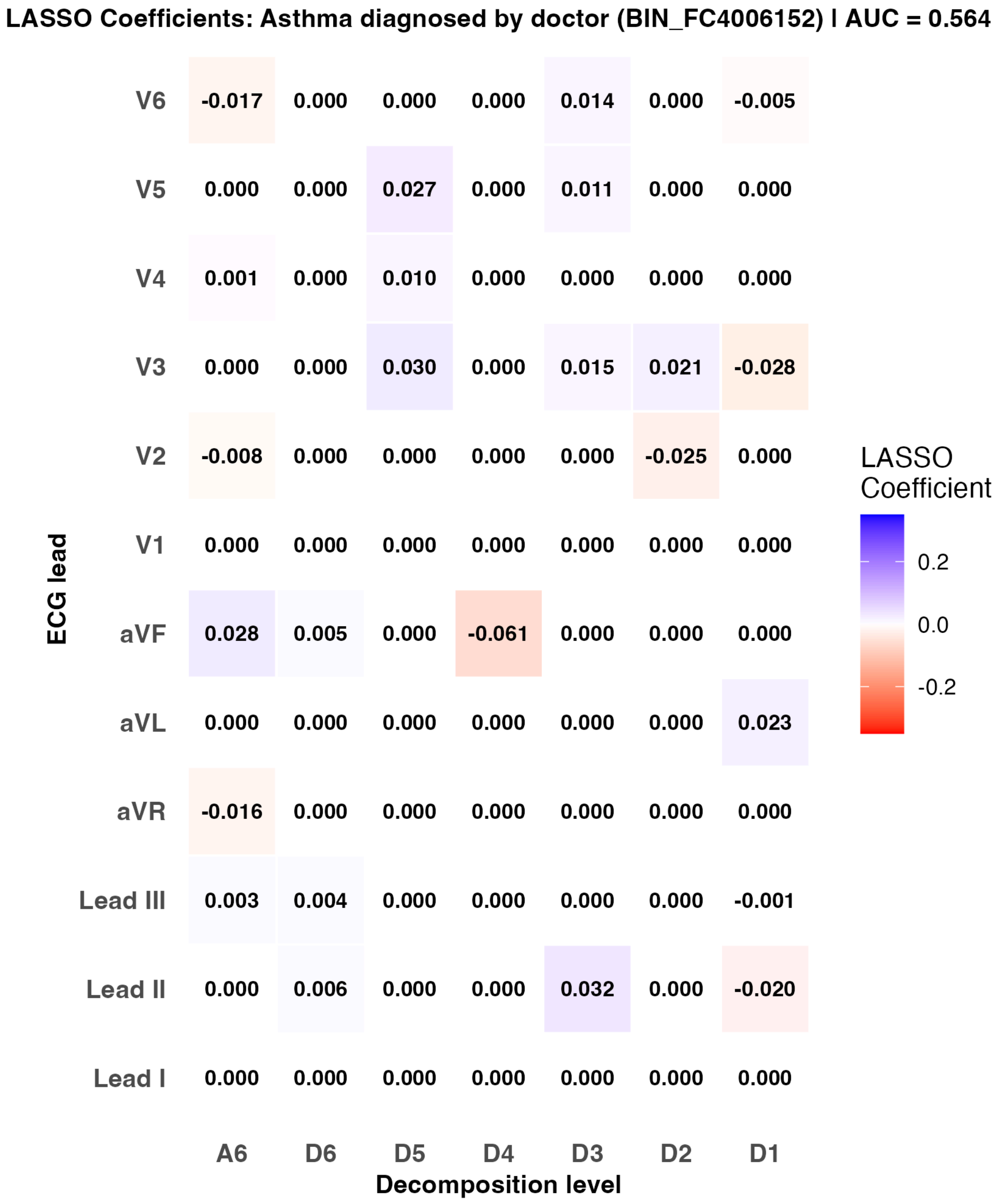

medRxiv preprint doi: https://doi.org/10.64898/2026.05.20.26353725; this version posted June 7, 2026. The copyright holder for this preprint (which was not certified by peer review) is the author/funder, who has granted medRxiv a license to display the preprint in perpetuity.
It is made available under a CC-BY 4.0 International license .

Supplementary Analysis 2: Independence of D1 cardiac signal from BMI genetics

To determine whether the genetic associations identified in the D1 frequency band (125–250 Hz) reflect cardiac biology rather than adiposity-related artifact, we conducted three complementary analyses.

Partial genetic correlation. We computed the partial genetic correlation between D1 energy and heart failure (HF) conditioning on BMI for all 12 leads, using the trivariate formula

$$r_{g,\text{partial}} = \frac{r_g(\text{D1, HF}) - r_g(\text{D1, BMI}) \cdot r_g(\text{BMI, HF})}{\sqrt{(1 - r_g^2(\text{D1, BMI}))(1 - r_g^2(\text{BMI, HF}))}}$$

where rg(BMI, HF) = 0.354 (SE = 0.041, $p = 1.2\times10^{-17}$, estimated by LDSC against FinnGen R12). The partial rg remained substantial across all leads with significant D1–HF associations. For V1 — the lead with the strongest association — the partial rg was 0.548 vs total rg = 0.559, a reduction of 2%. For leads V5 and V6, the partial rg exceeded the total rg (V5: 0.487→0.547; V6: 0.419→0.463), indicating that BMI acts as a suppressor for precordial leads rather than a confounder. Results for all 12 leads are shown in **Figure SA2a**.

BMI-conditioned GWAS. We repeated all 12 D1 GWAS with BMI as an additional covariate (Model B: energy_D1 ~ SNP + age + sex + PC1–10 + BMI) and compared effect sizes at the top loci against the primary GWAS (Model A). The lead SNP in the SCN5A region (Affx-89018181, chr3p22.2), encoding the cardiac sodium channel Nav1.5, showed beta attenuation of −1.9% to +1.6% across all 12 leads — negligible across the board. The only genome-wide significant TTN variant (rs34940894, V2 D1, $p = 4.6\times10^{-9}$) showed attenuation of −2.7%, with the effect slightly strengthened after conditioning, consistent with BMI acting as noise at this locus. All points fall within the ±10% band **(Figure SA2b)**.

Genetic correlations after BMI conditioning. LDSC genetic correlations between BMI-adjusted D1 summary statistics and FinnGen R12 HF were re-estimated for all 12 leads. V1 retained a significant association (rg = 0.363, SE = 0.128, p = 0.0045). Lead I (rg = 0.237, p = 0.033) and V6 (rg = 0.541, p = 0.037) also showed nominal significance. Results are shown in **Figure SA2a** .

Together these analyses demonstrate that D1 energy associations with cardiovascular disease are not explained by shared adiposity genetics, and that the top D1 GWAS loci encode cardiac rather than metabolic biology.

medRxiv preprint doi: https://doi.org/10.64898/2026.05.20.26353725; this version posted June 7, 2026. The copyright holder for this preprint (which was not certified by peer review) is the author/funder, who has granted medRxiv a license to display the preprint in perpetuity.
It is made available under a CC-BY 4.0 International license .

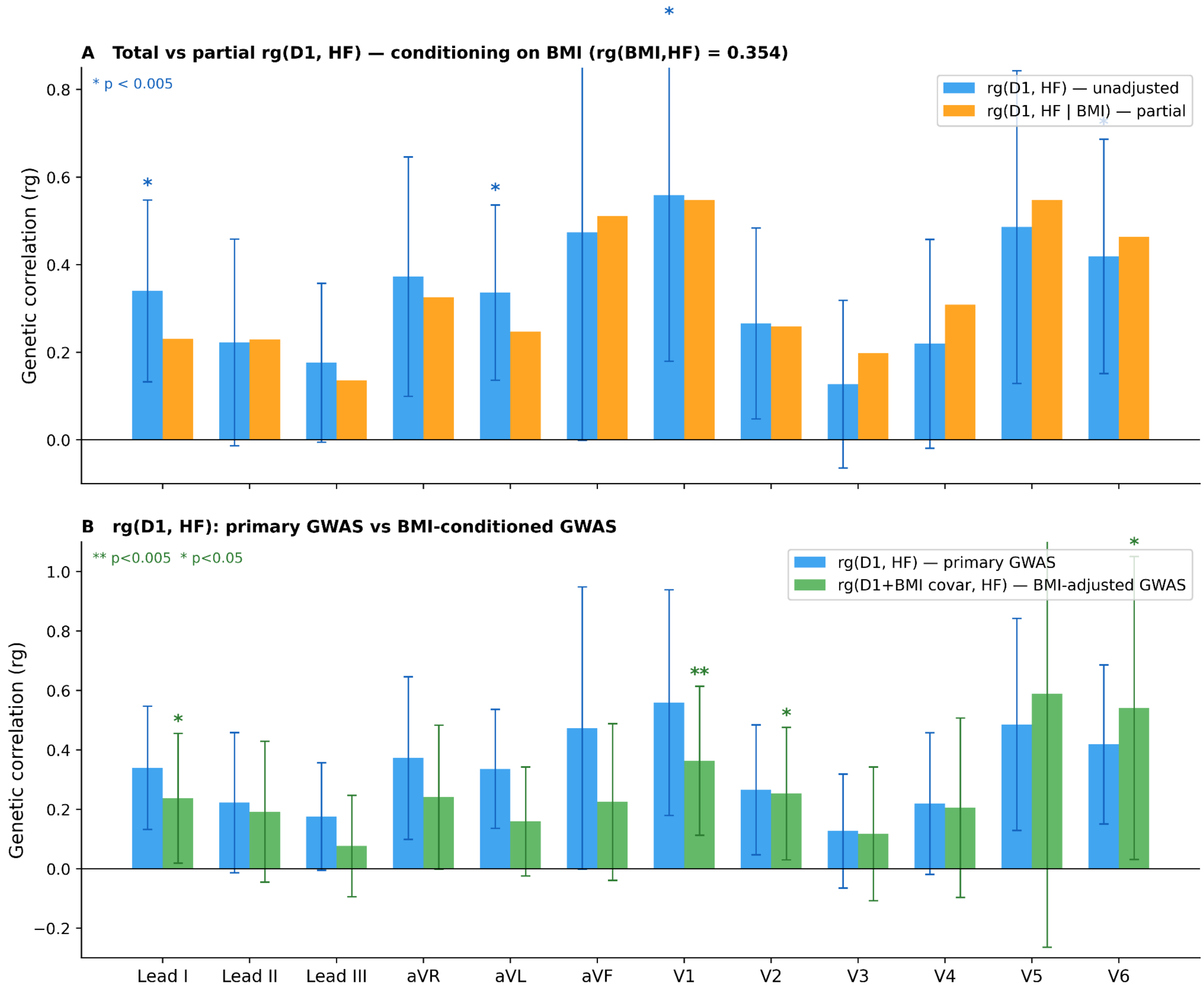

Figure SA2a. Two-panel bar plot comparing genetic correlations between D1 energy features and heart failure across 12 ECG leads. Each bar group shows three estimates: the primary genetic correlation rg(D1, HF) estimated from the original GWAS (blue), the partial genetic correlation rg(D1, HF | BMI) conditioning on BMI using the trivariate LDSC formula with rg(BMI, HF) = 0.354 (orange), and the genetic correlation estimated from BMI-conditioned GWAS summary statistics rg(D1+BMI, HF) (green). Error bars represent 95% confidence intervals (1.96 × SE). Significance markers indicate $p < 0.05$ (*) and $p < 0.005$ (**). Panel A shows the total vs partial rg, demonstrating that conditioning on BMI genetics produces negligible attenuation for most leads, with V1 retaining the strongest association (rg partial = 0.548 vs rg total = 0.559). Panel B shows the primary vs BMI-conditioned GWAS rg, with V1 remaining the only lead significant at $p < 0.005$ after BMI conditioning (rg = 0.363, p = 0.0045). Together, these panels demonstrate that the D1–heart failure genetic overlap is not mediated through shared adiposity genetics.

medRxiv preprint doi: https://doi.org/10.64898/2026.05.20.26353725; this version posted June 7, 2026. The copyright holder for this preprint (which was not certified by peer review) is the author/funder, who has granted medRxiv a license to display the preprint in perpetuity.
It is made available under a CC-BY 4.0 International license .

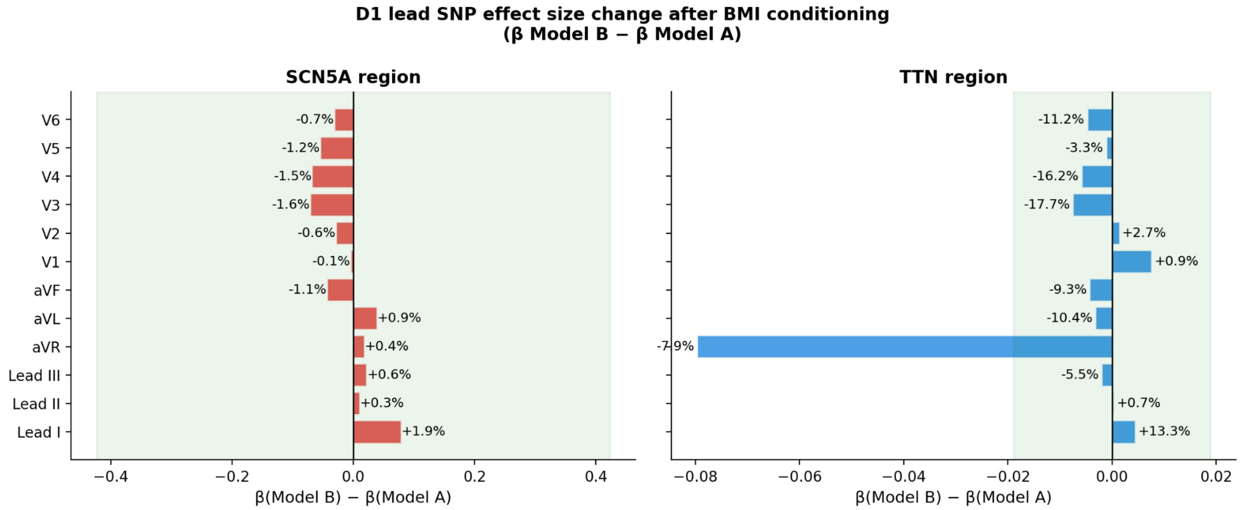

Figure SA2b. Horizontal bar plots showing the change in lead SNP effect size (Δβ = β Model B − β Model A) after adding BMI as a covariate, at the SCN5A region (chr3p22.2, left panel) and TTN region (chr2q31.2, right panel), across all 12 D1 leads. Model A: energy_D1 ~ SNP + age + sex + PC1–10; Model B: energy_D1 ~ SNP + age + sex + PC1–10 + BMI. Each bar represents one ECG lead. The green band indicates the ±10% attenuation zone. For SCN5A (Affx-89018181), all 12 leads show Δβ within ±2% (median attenuation 0.3%), confirming that the cardiac sodium channel locus is unaffected by BMI conditioning. For TTN, the median attenuation is 6.7%, with all leads remaining within the ±10% band. Bars near zero indicate that BMI does not explain the D1 genetic associations at these cardiac loci.
